# The phylogenetic significance of colour patterns in marine teleost larvae

**DOI:** 10.1111/zoj.12033

**Published:** 2013-07-08

**Authors:** Carole C Baldwin

**Affiliations:** Division of Fishes, National Museum of Natural History, Smithsonian InstitutionWashington, DC, 20560, USA

**Keywords:** chromatophore, erythrophore, fish larvae, iridophore, western Caribbean, xanthophore

## Abstract

Ichthyologists, natural-history artists, and tropical-fish aquarists have described, illustrated, or photographed colour patterns in adult marine fishes for centuries, but colour patterns in marine fish larvae have largely been neglected. Yet the pelagic larval stages of many marine fishes exhibit subtle to striking, ephemeral patterns of chromatophores that warrant investigation into their potential taxonomic and phylogenetic significance. Colour patterns in larvae of over 200 species of marine teleosts, primarily from the western Caribbean, were examined from digital colour photographs, and their potential utility in elucidating evolutionary relationships at various taxonomic levels was assessed. Larvae of relatively few basal marine teleosts exhibit erythrophores, xanthophores, or iridophores (i.e. nonmelanistic chromatophores), but one or more of those types of chromatophores are visible in larvae of many basal marine neoteleosts and nearly all marine percomorphs. Whether or not the presence of nonmelanistic chromatophores in pelagic marine larvae diagnoses any major teleost taxonomic group cannot be determined based on the preliminary survey conducted, but there is a trend toward increased colour from elopomorphs to percomorphs. Within percomorphs, patterns of nonmelanistic chromatophores may help resolve or contribute evidence to existing hypotheses of relationships at multiple levels of classification. Mugilid and some beloniform larvae share a unique ontogenetic transformation of colour pattern that lends support to the hypothesis of a close relationship between them. Larvae of some tetraodontiforms and lophiiforms are strikingly similar in having the trunk enclosed in an inflated sac covered with xanthophores, a character that may help resolve the relationships of these enigmatic taxa. Colour patterns in percomorph larvae also appear to diagnose certain groups at the interfamilial, familial, intergeneric, and generic levels. Slight differences in generic colour patterns, including whether the pattern comprises xanthophores or erythrophores, often distinguish species. The homology, ontogeny, and possible functional significance of colour patterns in larvae are discussed. Considerably more investigation of larval colour patterns in marine teleosts is needed to assess fully their value in phylogenetic reconstruction.

## Introduction

The pelagic larval stages of most marine fishes inhabit an evolutionary arena distinct from that of adults, and morphological specializations that presumably enhance survival in the planktonic realm have evolved in numerous teleost groups (Moser, 1981; Moser *et al*., 1984). In a few cases, marine fish larvae and adults are so different morphologically that they were initially classified as separate genera or families (Cohen, 1984; Johnson *et al*., 2009). Distinctive pigment patterns are among the transient features that characterize the pelagic larval phase of marine fishes (Moser *et al*., 1984), and Kendall, Ahlstrom & Moser (1984) included pigment patterns in their list of characters of early life-history stages commonly utilized in taxonomic and systematic studies of fishes. Although pigment patterns were noted by Kendall *et al*. (1984) to be among the most useful larval characters at specific and generic levels, only melanophores, not other types of chromatophores, were discussed. Chromatophores of poikilothermic vertebrates are dermal pigment units that comprise light-absorbing erythrophores (red/orange pigment), xanthophores (yellow pigment), and melanophores (dark brown/black pigment), as well as light-reflecting iridophores (structural colour, often silver/blue but many colours possible) – e.g. Bagnara & Hadley (1973), Grether, Kolluru & Nersissian (2004). Colour patterns in adult marine fishes are well documented, particularly those of tropical reef fishes, but pigment other than melanin in marine fish larvae has received little attention. This is not surprising because erythrophores and xanthophores fade upon conventional preservation (melanophores generally do not), and plankton samples are typically preserved soon after capture. Only when larvae have been observed, illustrated, or photographed prior to preservation has colour in marine teleost larvae been reported in the scientific literature (e.g. Brownell, 1979; Baldwin & Smith, 2003; Yasir & Qin, 2007; Baldwin *et al*., 2009a; 2011; Miller, 2009; Wittenrich, Baldwin & Turingan, 2010).

The transient colour patterns in marine fish larvae generally bear little resemblance to those of adults and may result from different ontogenetic (larval and adult) populations of chromatophores (Nakamura *et al*., 2010). The ontogeny of pigment patterns in marine fishes is poorly understood relative to that of many freshwater fishes, especially zebrafishes (*Danio* spp.), which have been studied extensively (e.g. Johnson *et al*., 1995; Parichy *et al*., 2000; Parichy, 2003, 2006; Kelsh, 1984; Budi, Patterson & Parichy, 2011). In *Danio*, the colour pattern of the recently hatched fish transforms directly into the adult colour pattern through incorporation of embryonic chromatophores and differentiation of new chromatophores from stem cells at metamorphosis (Parichy, 2003, 2006). There is no pelagic larval stage in *Danio* and most other freshwater fishes comparable to that in most marine fishes, and there is no accompanying distinctive pigment phase between the recently hatched and adult stages (Bagenal & Nellen, 1980; Kendall *et al*., 1984). Colour variation in this ‘extra’ pigment phase in marine fish larvae was the focus of this study. The presence of a specialized larval stage in certain freshwater fishes that are evolutionarily derived from marine fishes corroborates the distinction between early life stages of most marine and freshwater fishes. For example, larvae of basal freshwater percoids typically lack the head spination characteristic of pelagic larvae of basal marine percoids, but young *Lates* from Lake Tanganyika retain head spination that evolved in their marine, Indo-Pacific ancestors (Kinoshita & Tshibangu, 1997).

Colour patterns in the young of some freshwater fishes are highly conserved and thus of little potential phylogenetic value. For example, Quigley *et al*. (2004) noted that the young of several *Danio* species have virtually indistinguishable pigment patterns, and Kelsh (1984) noted the same for five *Danio* species and *Tanichthys albonubes*. In contrast, Baldwin & Smith (2003) and Baldwin *et al*. (2009a, 2011) highlighted the utility of patterns of erythrophores and xanthophores in species identification of larval Gobiidae and Apogonidae, and Baldwin *et al*. (2011) suggested that chromatophore patterns may be of value in resolving the generic classification of western Atlantic Apogonidae. The potential utility of larval colour patterns at higher taxonomic levels has not been properly investigated. Kendall *et al*. (1984) noted that pigment (melanophore) patterns in fish larvae are of limited use in systematic studies in part because convergence has resulted in the occurrence of strikingly similar patterns in unrelated groups. Convergent evolution can be detected if pigment characters are examined in a phylogenetic context. A phylogenetic tree that includes clades composed primarily of marine teleosts was used herein to examine the distribution of nonmelanistic chromatophores in larvae among major groups of marine teleosts. The exclusion of clades of freshwater fishes renders the tree but a partial view of teleost phylogeny, but in this first attempt to provide comparative information on colour patterns among marine teleost larvae, it is desirable to contemplate the results from both broad and more focused phylogenetic perspectives. Numerous recent hypotheses of phylogenetic relationships among various groups of teleosts were utilized for comparisons at lower taxonomic levels. The purposes of this paper were to describe the distribution of nonmelanistic chromatophores among a broad spectrum of marine teleost larvae and comment on the potential of selected chromatophore patterns to inform phylogeny.

## Material and Methods

This study was based largely on colour photographs of fish larvae collected off Belize, Central America. Larvae were collected in a plankton net of 505 μm mesh fitted onto a 0.5 × 1 m rectangular frame and deployed from a dock at Carrie Bow Cay (16°48.5′N, 88°05′W). Specimens were submerged in a photo tank, photographed with a Nikon D1 or Fuji FinePix S3 digital camera, and then tissue sampled for DNA analysis prior to preservation. Species identification of larvae was accomplished by matching cytochrome oxidase-*c* subunit I (COI) sequences (DNA barcodes) of larvae to those of known adults (Weigt *et al*., 2012). To date this protocol has resulted in the identification of larvae of approximately 170 Caribbean fish species. A few larvae had no species-level matches in the Smithsonian DNA database or the Barcode of Life Database (BOLD; http://www.boldsystems.org/views/login.php) and are identified only to genus in the figure legends. Taxonomic coverage was increased by examining images of marine fish larvae from specimens collected off the east coast of South Africa, Florida Straits, Hawaii, and Cozumel, as well as several Indo-Pacific species reared from aquarium specimens. Connell's (2007) website of early life-history stages of South African marine fishes is impressive in scope, and although few of his images are reproduced here, numerous references to his site and uniform resource locators (URLs) to specific pages are provided herein. All photo editing was carried out by the author. Images of most larvae were cut from their original photographic backgrounds and placed on a uniform background using the MaskPro 4 plug-in for Adobe Photoshop with the aid of a Wacom table and stylus. In many cases, the transparent fins of larvae were difficult to see, and shapes of fins may be approximations. Photo credits are provided in figure legends, and affiliations for contributors other than the author are as follows: Allan Connell, South African Institute of Aquatic Biology; Donald Griswold, formerly Smithsonian Institution; Cedric Guigand, University of Miami, Rosensteil School of Marine Science; Joshua Lambus, J. Lambus Photography, Hawaii; Michael Miller, The University of Tokyo; Julie Mounts, formerly Smithsonian Institution; Christopher Paparo, The Long Island Aquarium; David Smith, Smithsonian Institution; Lee Weigt, Smithsonian Institution; and Matthew Wittenrich, University of Florida. No professional affiliations are available for two additional contributors, Matthew D'Avella and Donald Hughes. Localities from which specimens were collected or photographed are provided in figure legends: BLZ refers to Belize, and it is followed by a four- or five-digit number that represents the Smithsonian DNA number. DNA barcodes of Belizean fish larvae are publicly available on BOLD under the project names APG, BATHY, BZLWA, BZLWB, BZLWC, BZLWD, BZLWE, CORY, PHAE, and RYP. GenBank accession numbers for COI sequences are as listed in Baldwin *et al*. (2009a, 2009b, 2011), Tornabene *et al*. (2010), Baldwin & Weigt (2012), and Weigt *et al*. (2012). Not all images examined were reproduced in this paper. A complete list of larval-fish images examined for colour patterns is given in Appendix, which also includes URLs for some of the supplementary images examined.

In the descriptions of nonmelanistic chromatophore patterns, ‘yellow pigment’ and ‘xanthophores’ have been used interchangeably, as have ‘orange pigment’ (or ‘red pigment’) and ‘erythrophores’. The presence or absence of yellow and orange pigment was equated with the presence or absence of xanthophores and erythrophores, respectively, and the presence or absence of light-reflecting pigment was equated with the presence or absence of iridophores. Assessments of types of nonmelanistic chromatophores were based solely on examination of fresh specimens and colour photographs, not histological examination.

**Figure 1:**
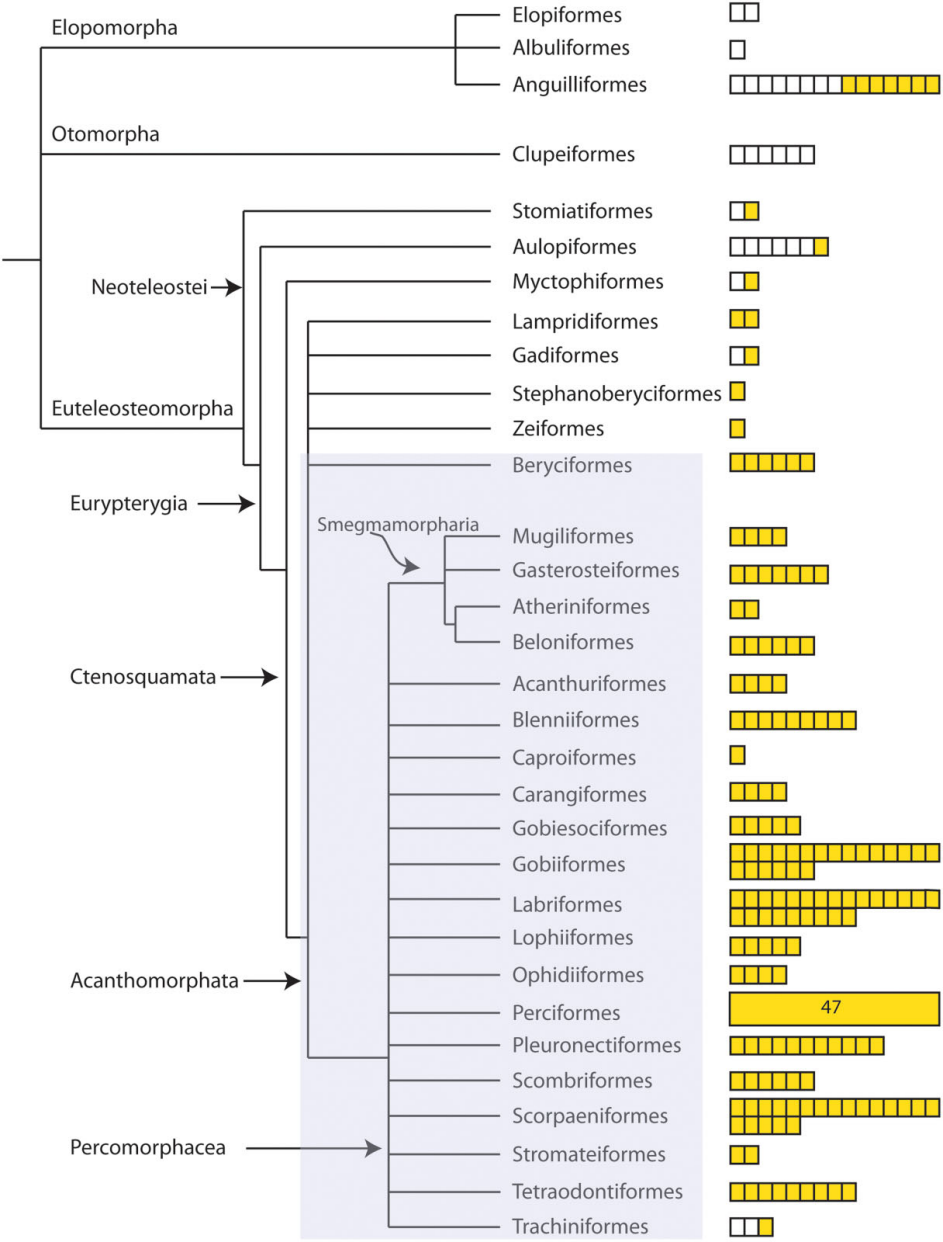
Teleost phylogeny based on the classification of Wiley & Johnson (2009). Only marine taxa for which colour in larvae was examined are included. The number of squares associated with each teleost order is equivalent to the number of species examined. Open squares indicate the absence of xanthophores, erythrophores, and iridophores. Yellow squares indicate the presence of one or more of these types of chromatophores. The shaded rectangle denotes Johnson & Patterson's (1993) Euacanthopterygii.

The classification of Wiley & Johnson (2009) was selected for use in this study. This classification was chosen over other more commonly used fish classifications because it is a Linnaean classification based on monophyletic groups. It was essential in this work to examine the distribution of nonmelanistic chromatophores in fish larvae among major teleost groups from an evolutionary perspective, and constructing a teleost phylogeny from the Wiley & Johnson (2009) synapomorphy-based classification was easily accomplished (Fig. 1).

## Results

### Distribution of nonmelanistic chromatophores in basal marine teleosts and neoteleosts (Fig. 1)

**Figure 2:**
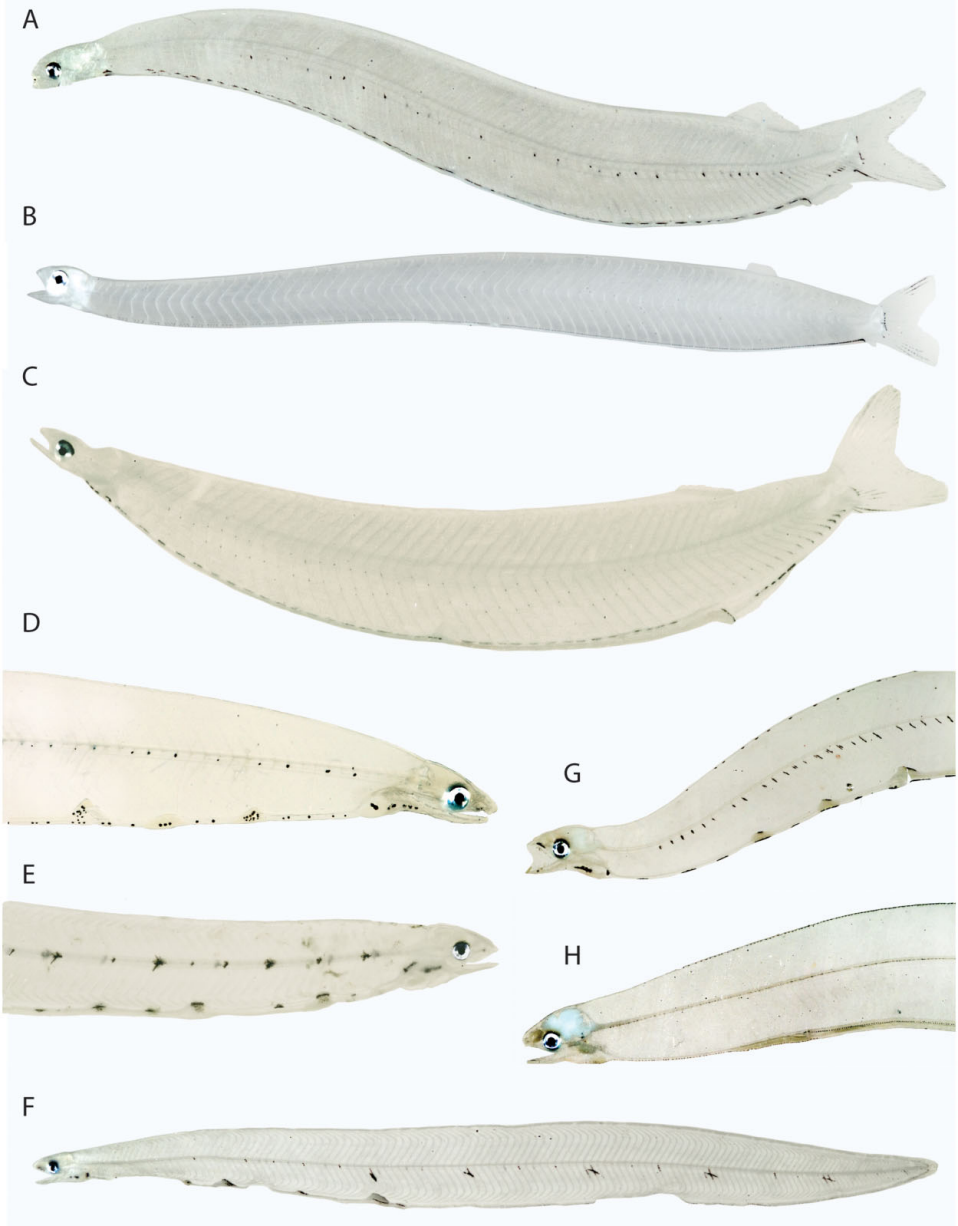
Elopomorpa. A, *Elops saurus*, 31 mm Standard Length (SL), BLZ 8327. B, *Albula vulpes*, 54 mm SL, BLZ 8420. C, *Megalops atlanticus*, 23 mm SL, BLZ 5457. D, *Myrophis punctatus*, 58 mm SL, Belize. E, *Aprognathodon platyventris*, 75 mm SL, BLZ 5322. F, *Myrophis platyrhynchus*, 67 mm SL, BLZ 8392. G, *Ahlia egmontis*, 70 mm TL, BLZ 7174. H, *Gymnothorax moringa*, 71 mm SL, BLZ 8469. Photos A, C, F by Lee Weigt and Carole Baldwin; B, G, H by Julie Mounts and David Smith; D, E by Julie Mounts and Carole Baldwin.

**Figure 3:**
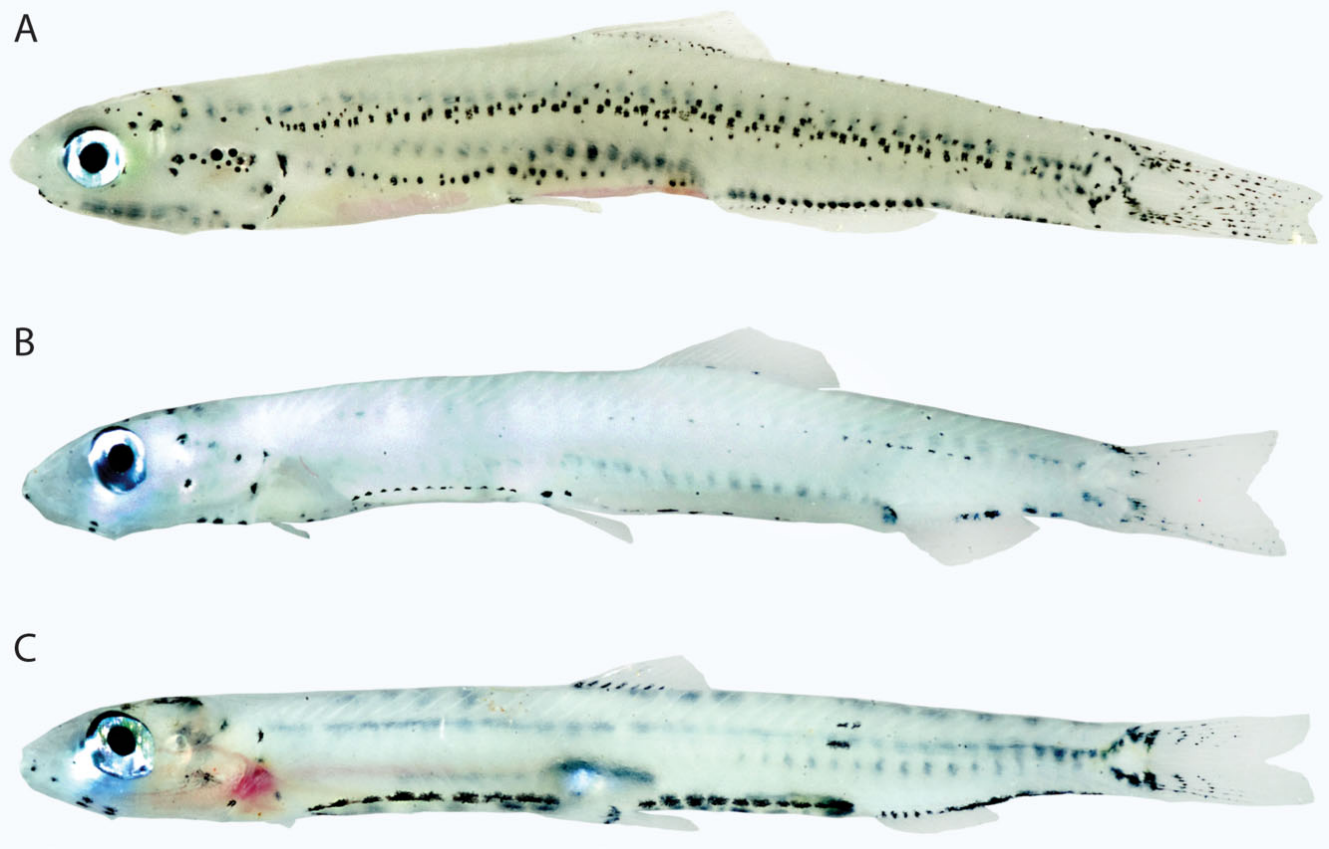
Otomorpha (Clupeiformes). A, *Anchoa* sp., 26 mm Standard Length (SL), BLZ 7162. B, *Harengula clupeola*, 15 mm SL, BLZ 8419. C, *Jenkensia lamprotaenia*, 15 mm SL, BLZ 8417. Note: the red coloration behind the head in *J. lamprotaenia* is associated with the circulatory system, not chromatophores. Photos by Julie Mounts and David Smith.

**Figure 4:**
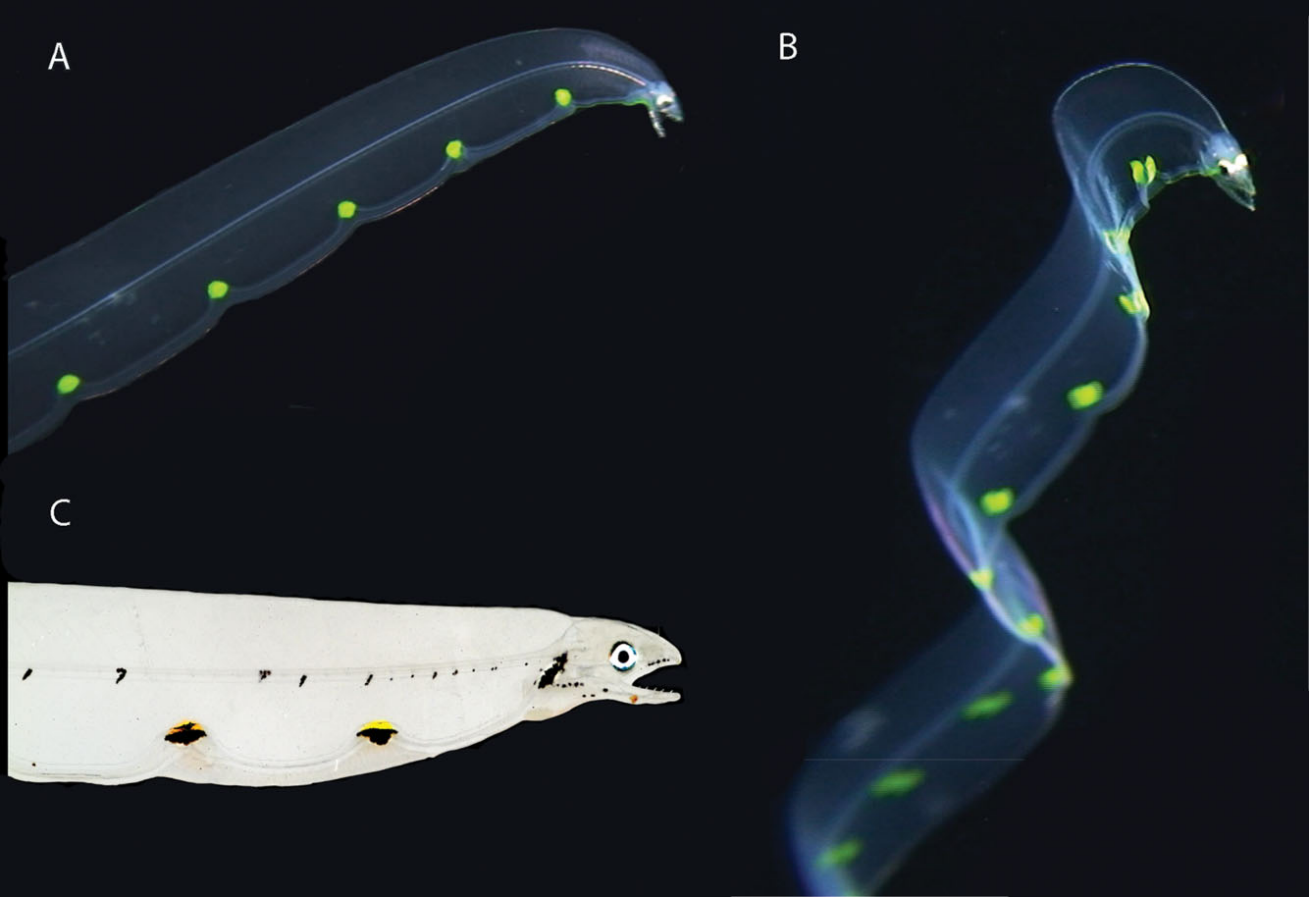
Elopomorpha. A, B, *in situ* images of an ophichthid leptocephalus off Hawaii captured from video by Matthew D'Avella, Kona, Hawaii (B previously published in Miller *et al*., 2010, reproduced here with permission of the copyright holder). C, *Myrichthys breviceps*, 116 mm Standard Length, BLZ 8467, photo by Julie Mounts and David Smith.

**Figure 5:**
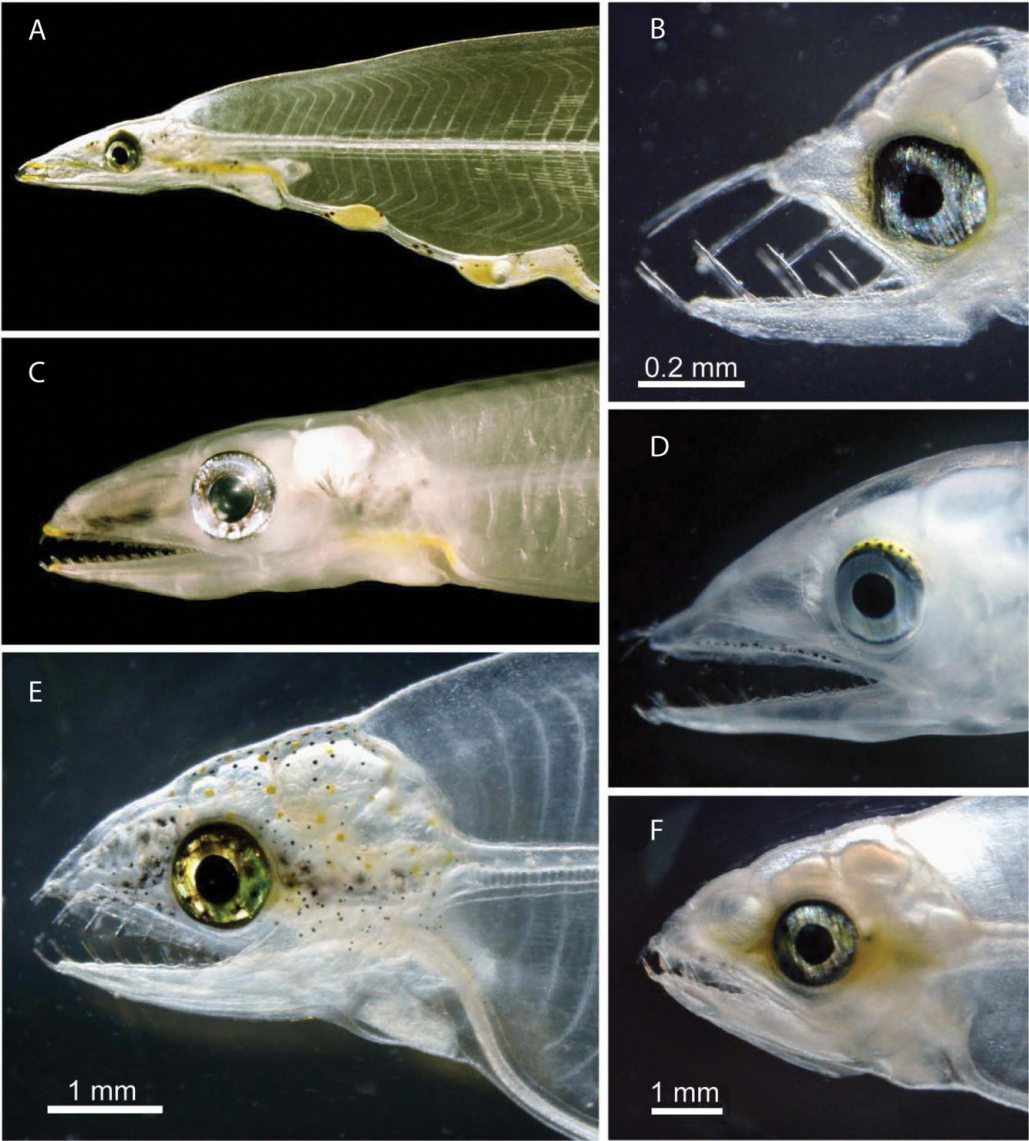
Elopomorpha. A, *Neenchelys* sp. (Ophichthidae). B, E, F, Muraenidae. C, *Saurenchelys* sp. (Nettastomatidae). D, Ophichthidae. Modified from Miller (2009) with the permission of the copyright holder.

Larvae of most basal teleosts – elopiforms, albuliforms, anguilliforms, and clupeiforms – do not exhibit xanthophores, erythrophores, or iridophores (Figs 2, 3). Exceptions occur within the anguilliform families Ophichthidae, Muraenidae, Congridae, and Nettastomatidae. Miller, D'Avella & Tsukamoto (2010: figs 1, 2) described xanthophores along the bodies of two unidentified ophichthid leptocephalus larvae videotaped swimming at night off Hawaii (Fig. 4A, B). An image of the ophichthid *Neenchelys* (Miller, 2009: fig. 57A) has yellow pigment on the snout, anterior portion of the oesophagus, and on the gut swellings (Fig. 5A). Another ophichthid leptocephalus, *Myrichthys breviceps*, captured off Belize bears similar conspicuous xanthophores on the gut swellings (Fig. 4C). Tawa *et al*. (2012: fig. 2) published a colour image of a muraenid leptocephalus (*Strophidon ui*) that exhibits xanthophores in front of and behind the eye, and Miller (2009) provided images (Fig. 5B, E, F herein) of muraenid leptocephali with similar yellow pigment adjacent to the eye (and in one case also scattered on the head). Miller (2009) noted the presence of yellow pigment on the dorsal surface of the eye in some congrids and ophichthids (Fig. 5D) and yellow pigment on the snout and anterior portion of the oesophagus in the nettastomatid *Saurenchelys* (Fig. 5C). Identification of more anguilliform larvae is needed to determine the taxonomic distribution of xanthophores, but the presence of yellow pigment on gut swellings in ophichthids, on the snout and anterior oesophagus in ophichthids and nettastomatids, in front of and behind the eye in muraenids, and dorsal to the eye in congrids and ophichthids might represent diagnostic patterns and therefore warrant additional study. Most leptocephali collected off Belize lack yellow pigment, yet many are members of families discussed above that have it. Anguilliform leptocephali from Belize that lack yellow pigment (Fig. 2) include *Gymnothorax moringa* (Muraenidae), *Moringua edwardsi* (Moringuidae), *Chilorhinus seunsoni* (Chlopsidae), and *Ahlia egmontis*, *Aprognathodon platyventris*, *Myrophis punctatus*, and *Myrophis platyrhynchus* (Ophichthidae). Based on the absence of xanthophores in larval albuliforms and elopiforms, it is reasonable to assume that their absence is ancestral for anguilliforms. The absence of yellow pigment in leptocephali of *Moringua edwardsi* and Synaphobranchidae (Miller, 2009) provides corroborative evidence based on the basal positions of Moringuidae and Synaphobranchidae in the molecular anguilliform phylogeny of Tang & Fielitz (2012). Anguilliform taxa that exhibit yellow pigment in the leptocephalus stage – some Congridae, Nettastomatidae, Ophichthidae, and muraenine Muraenidae – occupy more distal phylogenetic positions in the order (Tang & Fielitz, 2012), but they do not constitute a monophyletic assemblage. It seems likely that xanthophores in larvae evolved independently within the various families of Anguilliformes that exhibit them.

**Figure 6:**
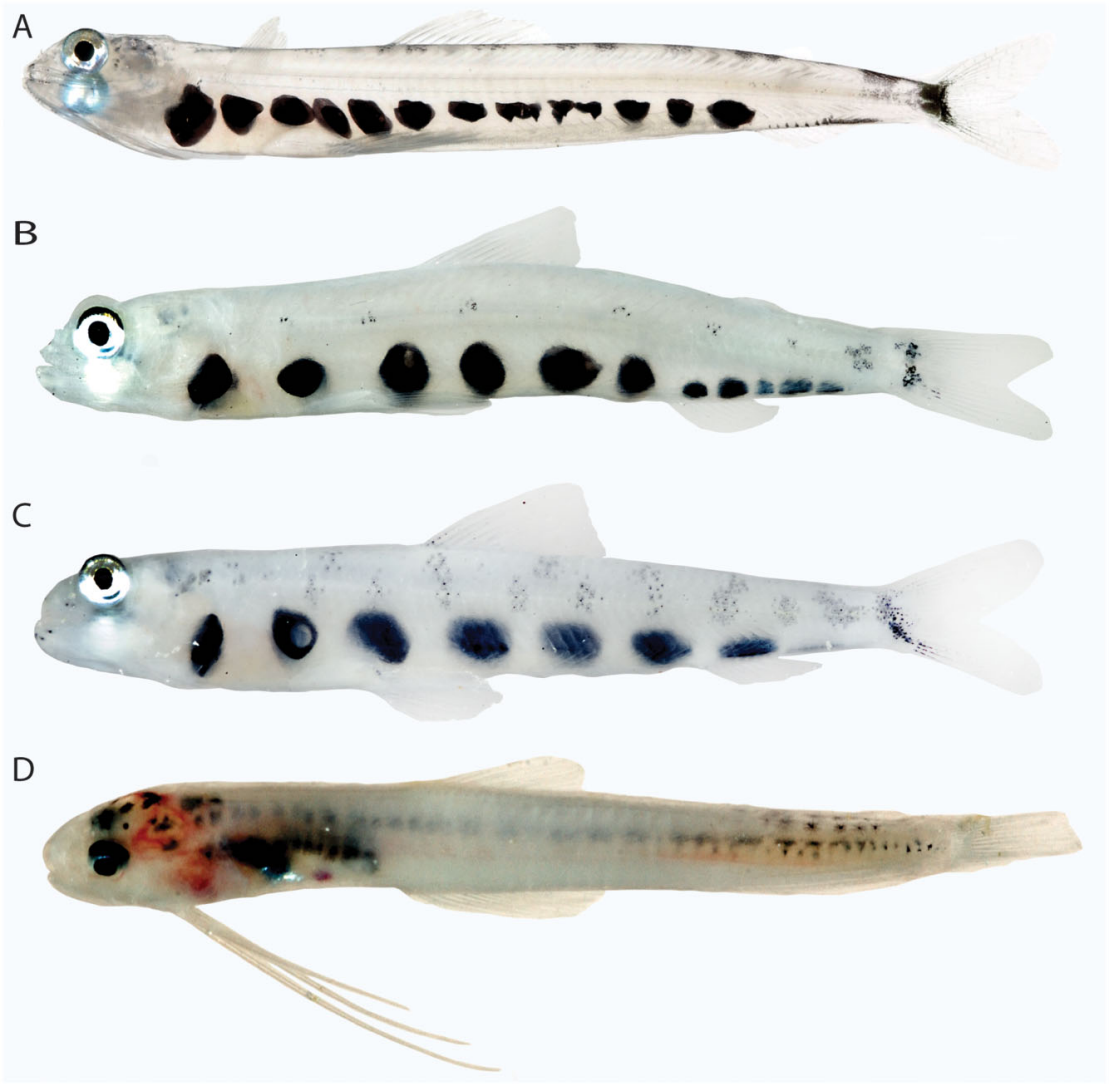
Neoteleostei (Aulopiformes, Myctophiformes) and Acanthomorphata (Gadiformes). A, *Synodus synodus*, 39 mm Standard Length (SL), Belize. B, *Saurida* sp., 26 mm SL, BLZ 8329. C, *Saurida* sp., 32 mm SL, BLZ 8398. D, *Bregmaceros* sp., 12 mm SL, BLZ 4242. Note: the red coloration on the head and body in the *Bregmaceros* image appears to be associated with the circulatory system, not dermal pigment. Photo A by Julie Mounts and Carole Baldwin; B Lee Weigt and Carole Baldwin; C Julie Mounts and David Smith; D Lee Weigt and David Smith.

**Figure 7:**
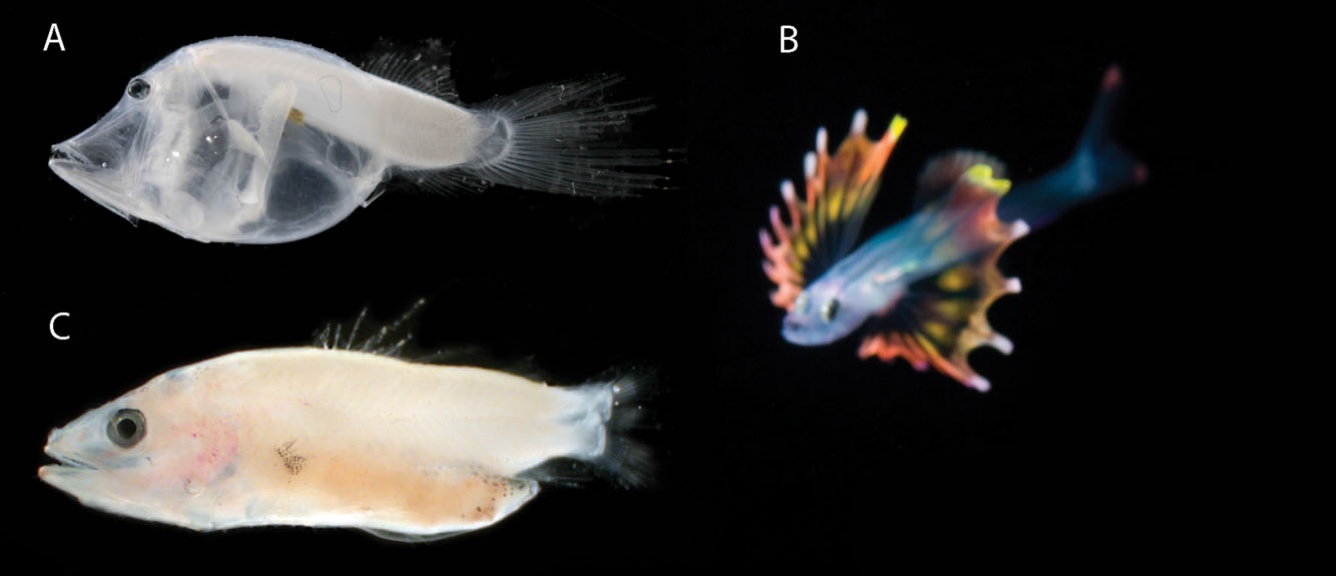
Neoteleostei (Aulopiformes, Myctophiformes) A, *Gigantura* sp., Florida Straits, photo by Cedric Guigand (previously published in Pineda, Hare & Sponaugle, 2007). B, unknown aulopiform, Hawaii, photo by Joshua Lambus. C, unknown myctophiform, Florida Straits, photo by Cedric Guigand.

**Figure 8:**
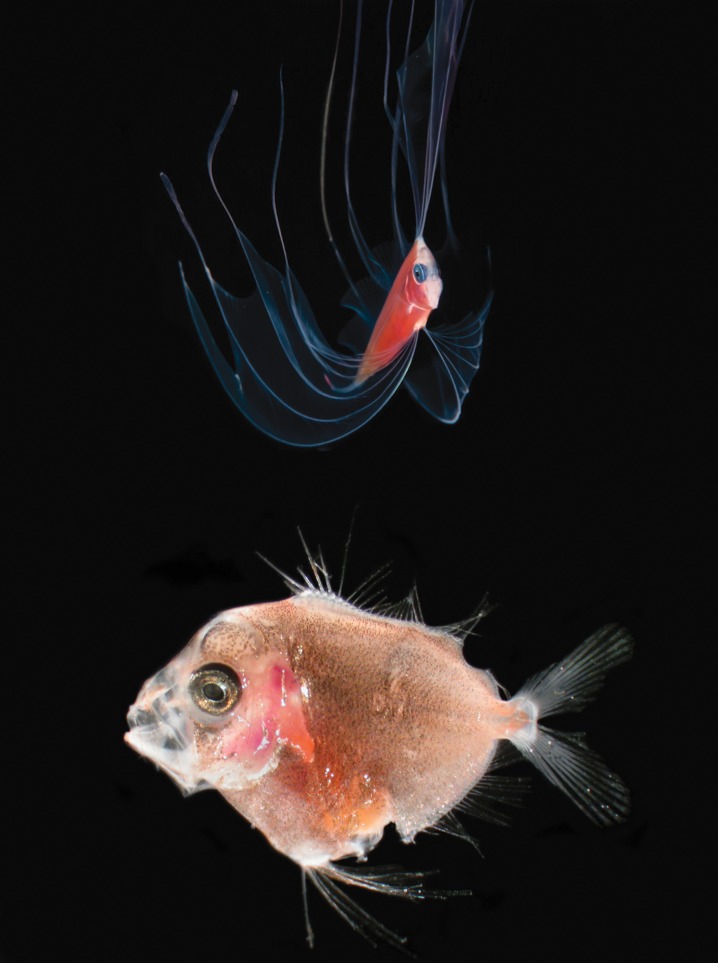
Acanthomorphata (Lampridiformes). Top, unknown (probably Trachipteridae), Hawaii, photo by Joshua Lambus. Bottom, *Lampris guttatus*, Florida Straits, photo by Cedric Guigand.

**Figure 9:**
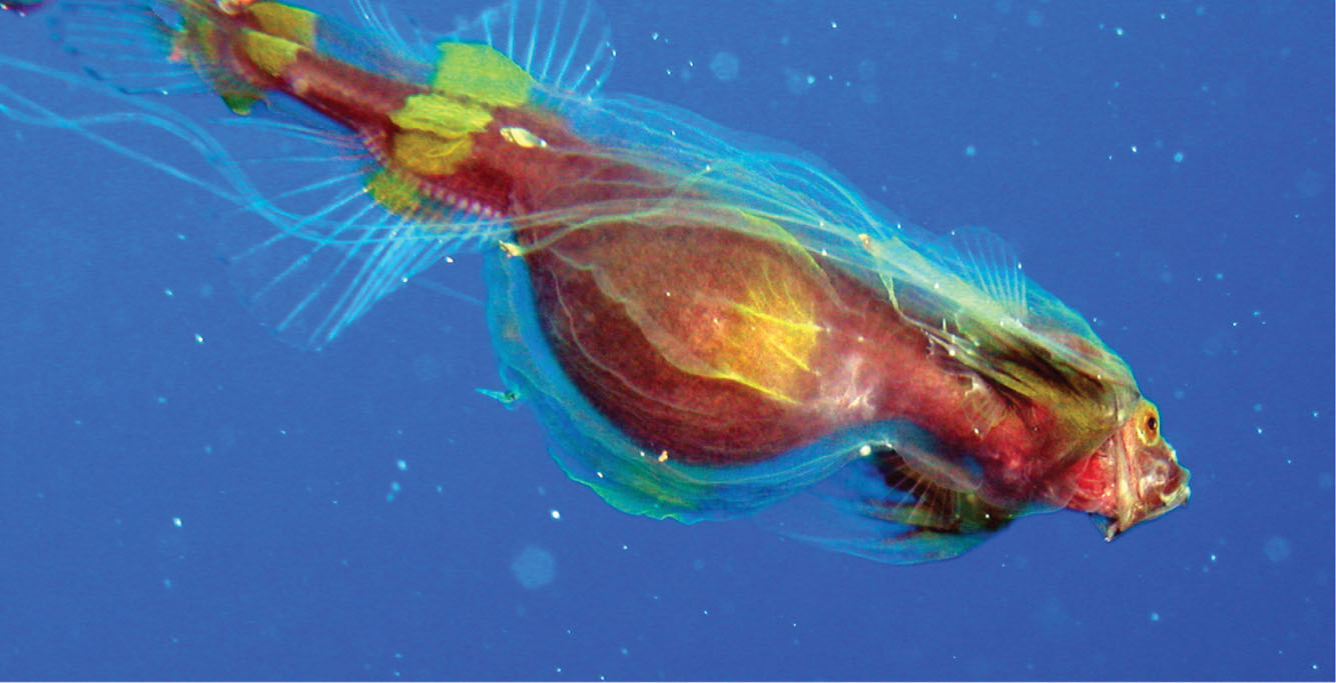
Acanthomorphata (Stephanoberyciformes). Cetomimidae, Mexico. Photo by Donald Hughes.

**Figure 10:**
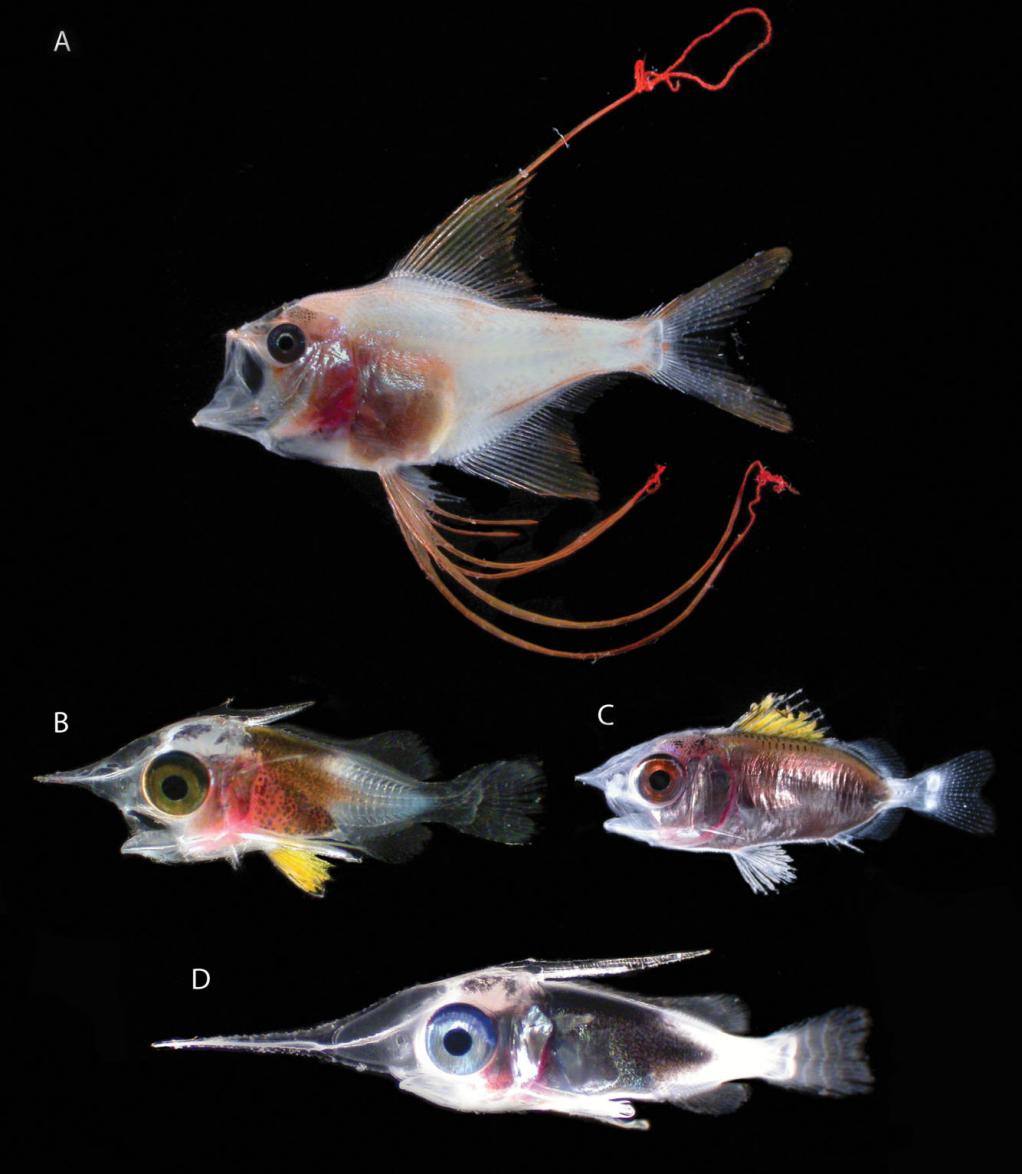
Acanthomorphata (Beryciformes). A, Berycidae. B–D, Holocentridae. All from Florida Straits. Photographs by Cedric Guigand. A, B, and D previously published in Cowen *et al*. (2007), Thorrold, Zacherl & Levin (2007), and Gaines *et al*. (2007), respectively.

Little information is available on the presence or absence of nonmelanistic chromatophores in larvae of basal marine neoteleosts (Fig. 1). Recently hatched larvae of one phosichthyid stomiatiform from off South Africa lack erythrophores and xanthophores, whereas a preflexion larva of a melanostomiatid has yellow pigment on the head and body (Connell, 2007; see links to images in Appendix). Two aulopiform families (Synodontidae and Giganturidae) also have larvae that lack orange or yellow coloration (Figs 6A–C, 7A), but an unidentified aulopiform larva has vibrant erythrophores and xanthophores on enlarged pectoral fins as well as on the dorsal and caudal fins (Fig. 7B). A preflexion myctophiform larva from off South Africa has yellow pigment on the head and body, but yellow pigment is not as evident in a larger preflexion larva (Connell, 2007; Appendix). A postflexion larval myctophiform from the Florida Straits lacks erythrophores and xanthophores (Fig. 7C). In gadiforms, preflexion larvae of an unidentified gadid from South Africa have numerous xanthophores on the head and body (Connell, 2007; Appendix), whereas the bregmacerotid *Bremaceros* from Belize lacks orange and yellow pigment (Fig. 6D). Both lampridiform larvae examined (a trachipterid and *Lampris*) have nearly the entire body and some fins covered with erythrophores (Fig. 8). The single stephanoberyciform larva examined, the bizarre ‘mirapinnid’ larva of the whalefish family Cetomimidae (Johnson *et al*., 2009), has yellow pigment on the body and fins (Fig. 9), and the single zeiform examined, *Zeus faber* (Connell, 2007; Appendix), also has a small bit of yellow pigment in both pre- and postflexion stages that is mixed with and largely occluded by melanophores. All preflexion and postflexion beryciform larvae have erythrophores, xanthophores, iridophores, or some combination of those chromatophores (Fig. 10).

To summarize the comparative information for basal marine teleosts and neoteleosts, several basal neoteleost orders are similar to anguilliforms in having some larvae that exhibit nonmelanistic chromatophores and others that do not. Xanthophores are the only types of chromatophores observed in larval anguilliforms and in the single larval stomiatiform examined that has nonmelanistic chromatophores. Xanthophores are lacking in larval elopiforms, albuliforms, and the few marine clupeiforms examined. Xanthophores are not lacking in all otomorphs, however, as they have been documented in freshwater Cyprinidae (e.g. Johnson *et al*., 1995; Parichy *et al*., 2000; Parichy, 2003). Erythrophores are first observed phylogenetically in marine fish larvae in aulopiforms and are prominent in lampridiforms and some beryciforms. Possibly erythrophores in larvae are phylogenetically significant at the level of Eurypterygia (Aulopiformes and above, Fig. 1). Iridophores first appear phylogenetically in larval beryciforms and could provide corroborative evidence for Johnson & Patterson's (1993) Euacanthopterygii (Beryciformes and above, shaded rectangle in Fig. 1). Iridophores are lacking in larvae of the marine elopomorphs, otomorphs, and other basal neoteleosts examined; however, they are present in some recently hatched freshwater Otomorpha (e.g. *Danio* – Johnson *et al*., 1995; Parichy *et al*., 2000; Parichy, 2003). As discussed below, an ontogenetic transition involving iridophores in atheriniforms, beloniforms, gasterosteiforms, and mugiliforms, which are members of the percomorph series Smegmamorpharia, may help diagnose that group. Within percomorphs (Mugiliformes and above, Fig. 1), erythrophores, xanthophores, iridophores, or some combination of them are present in larvae of every group and in almost every species examined, and the remainder of this paper is devoted to descriptions of percomorph colour patterns and discussions of their potential phylogenetic significance at various taxonomic levels.

### Percomorphacea – Smegmamorpharia

**Figure 11:**
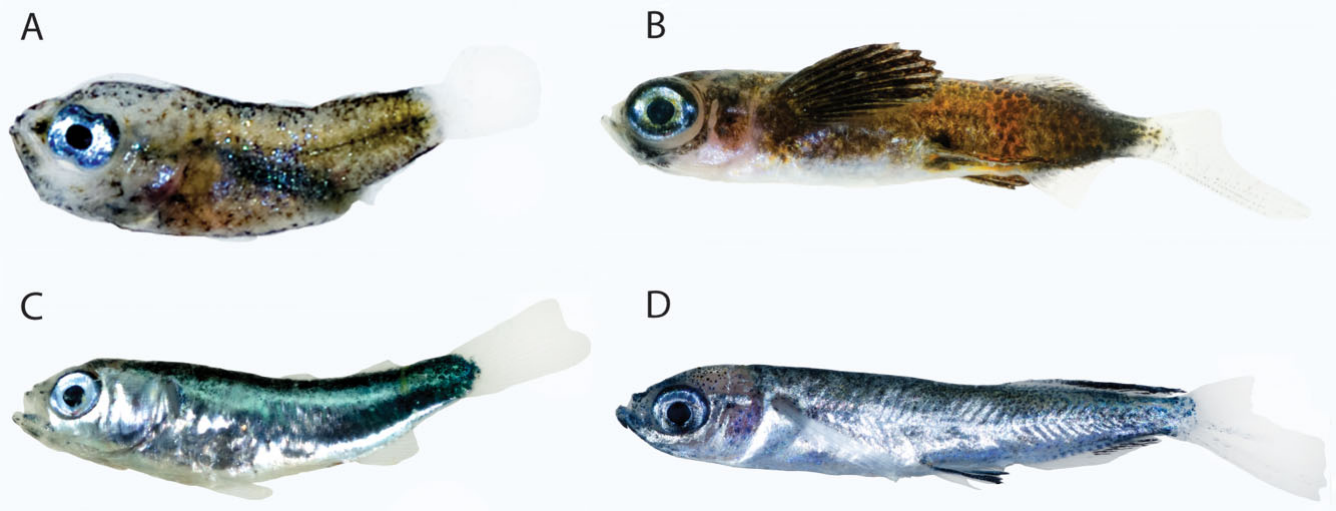
Percomorphacea (Mugiliformes and Beloniformes). A, *Mugil cephalus*, 3.5 mm Standard Length (SL), BLZ 6045. B, *Prognichthys occidentalis*, 11 mm SL, BLZ 7186. C, *Mugil* sp., 8.5 mm SL, BLZ 7069. D, Exocoetidae, 16 mm SL, BLZ 6014. Photos A, D by Lee Weigt and Carole Baldwin; B, C by Julie Mounts and David Smith.

**Figure 12:**
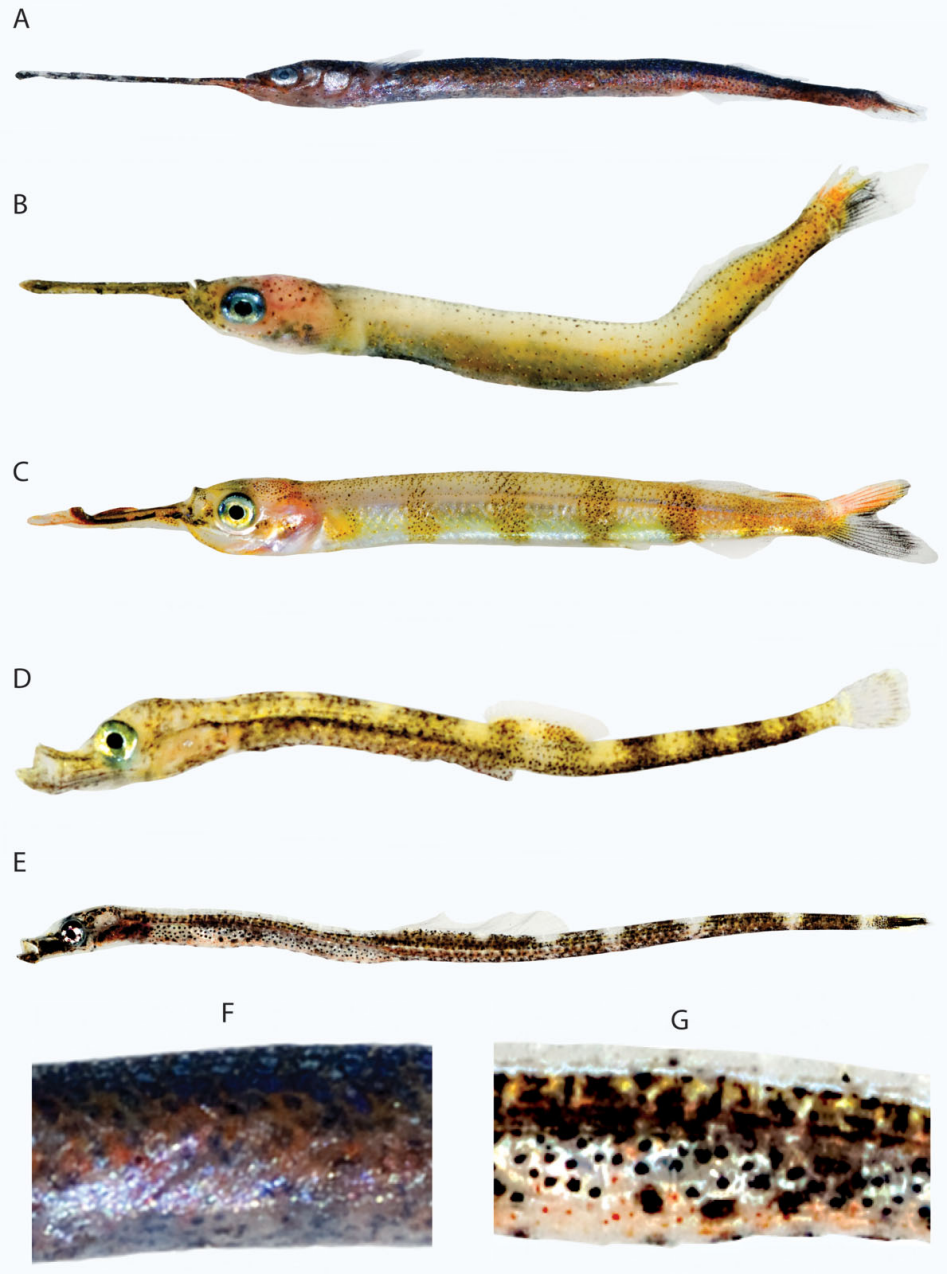
Percomorphacea (Beloniformes, Gasterosteiformes). A, *Platybelone argalus*, 15 mm Standard Length (SL), BLZ 6131. B, *Hemirhamphus brasiliensis*, 19.5 mm SL, BLZ 7071. C, *Hemirhamphus balao*, 34 mm SL, BLZ 7304. D, *Cosmocampus albirostris*, 8.5 mm SL, BLZ 6414. E, *Penetopteryx nanus*, 19 mm SL, BLZ 8337. F and G, close-up photographs of *Platybelone argalus* (A) and *Penetopteryx nanus* (E), respectively, showing abdominal iridophores. Photos A, D, E by Lee Weigt and Carole Baldwin; B, C by Julie Mounts and David Smith.

**Figure 13:**
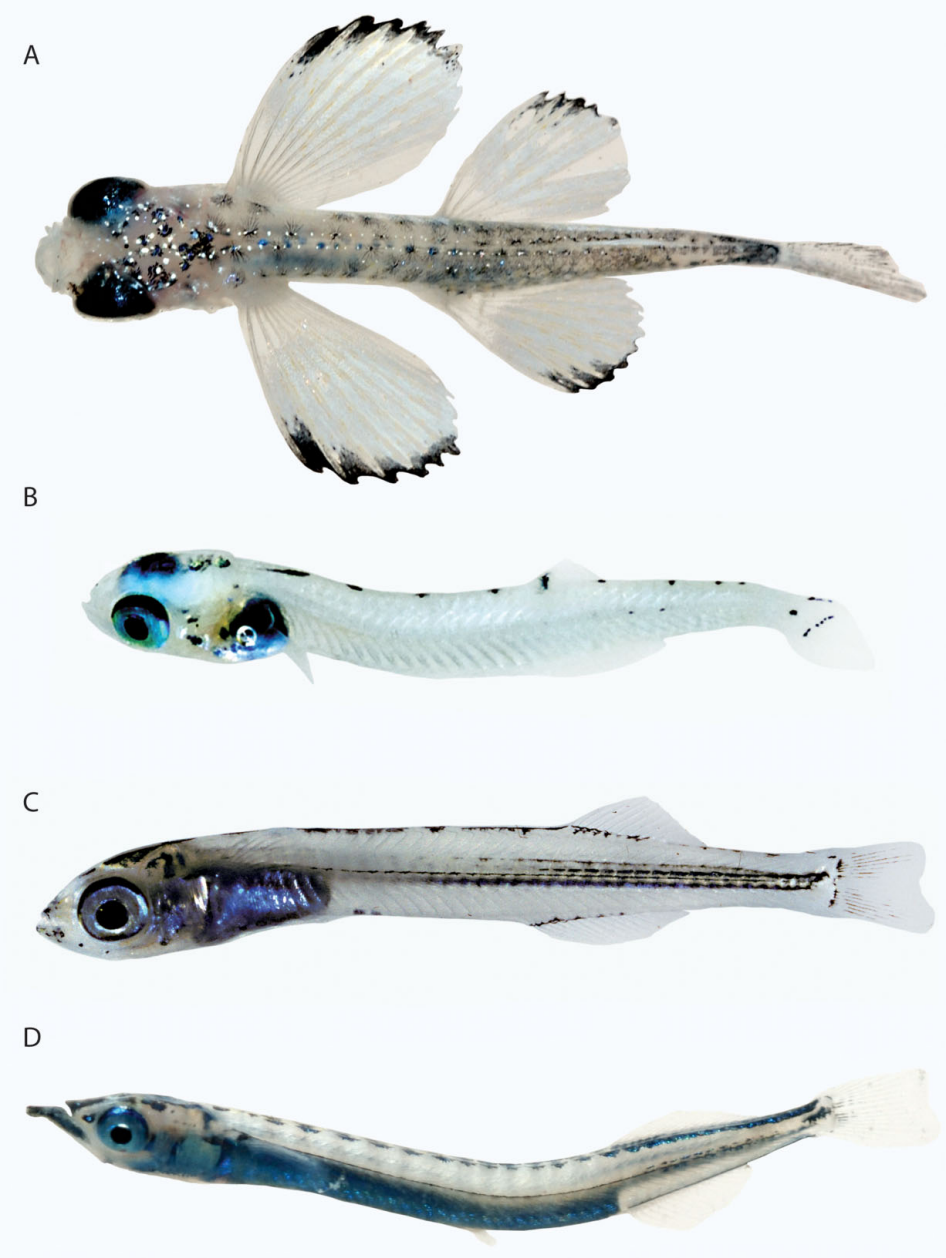
Percomorphacea (Beloniformes, Atheriniformes). A, *Hirundichthys affinis*, 13 mm Standard Length (SL), BLZ 4217. B, *Atherinomorus stipes*, 7 mm SL, BLZ 6051. C, Atherinidae, 14 mm SL, Belize, USNM 353871. D, Beloniformes (Hemirhamphidae?), 14 mm SL, BLZ 4113. Photo A by Lee Weigt and David Smith; B by Lee Weigt and Carole Baldwin; C by David Smith; D by Julie Mounts and Carole Baldwin.

Johnson & Patterson's (1993) Smegmamorpha comprise seven orders of percomorph fishes, but the monophyly of the group has been questioned. For example, Springer & Orrell (2004) did not find support for it based on extensive analysis of the gill-arch musculature and skeleton. Smegmamorph orders for which larvae were examined in this study are Atheriniformes, Beloniformes, Gasterosteiformes, and Mugiliformes. Larval mugilids and exocoetids examined share a striking ontogenetic transition in colour pattern. Small larvae are covered with pale orange/yellow pigment and melanophores but also bear some conspicuous iridophores (Fig. 11A, B). In larger larval specimens, the orange colour is no longer present, and the fishes are silvery and covered almost entirely with iridophores (Fig. 11C, D). This ontogenetic transition was not observed in any nonsmegmamorph fishes and would appear to provide corroborative evidence for a close relationship between Mugiliformes and Atherinomorpha, which comprises Atheriniformes and Beloniformes. Stiassny (1990, 1993) proposed a sister-group relationship between mugiliforms and atherinomorphs despite strong evidence also suggesting that mugilids are closely related to perciforms. This same colour transition occurs in hemirhamphids and belonids but apparently at a later stage of development. Larvae of *Platybelone* and *Hemirhamphus* are covered with erythrophores and xanthophores, respectively, and bear scattered iridophores (Fig. 12A, B, F). A juvenile *Hemirhamphus* still bears xanthophores, but the abdominal region is silvery (Fig. 12C), and adults of both genera are entirely silver. Larval gasterosteiforms also have yellow/orange chromatophores and melanophores covering most of the body as in young mugilids and beloniforms (Fig. 12D, E), and although they never undergo the transition to a silvery body, at least some gasterosteiform larvae have silvery pigment on the abdomen (Fig. 12E, G). Other gasterosteiform larvae known only from early preflexion stages – *Fistularia*, *Aulostomus*, *Aeoliscus –* also exhibit numerous xanthophores mixed with melanophores (Connell, 2007; Appendix), but later larval stages are needed to determine whether or not they develop iridophores. Some larval beloniforms may have a different larval trajectory in that they appear to lack the early yellow/orange stage. For example, a 5.6 mm Notochord Length (NL) larva of *Oxyporhamphus micropterus* from off South Africa is mostly pale, has melanophores and iridophores along the entire dorsal margin of the body and along a portion of the ventral midline, and has a silvery/blue gut (Connell, 2007; Appendix). Possibly there is a small bit of yellow pigment on the oesophagus and gut, but it was difficult to determine if this is dermal pigment or part of the gut contents. It is also possible that the yellow/orange stage develops after notochord flexion, as all mugilid, beloniform, and gasterosteiform larvae in which that colour phase was observed had undergone flexion. A 13-mm Standard Length (SL) *Hirundichthys* has only melanophores and iridophores (Fig. 13A), but younger larvae are unknown. Larval atherinids have few or no orange or yellow chromatophores (smallest specimen examined 7.0 mm SL – Fig. 13B) and have a dense covering of iridophores on the gut. Note the striking similarity between a 14-mm SL atherinid larva and an unidentified beloniform larva in general appearance and the presence of iridophores on the gut that reflect bright blue (Fig. 13C, D).

### Percomorphacea – incertae sedis

#### Acanthuriformes

**Figure 14:**
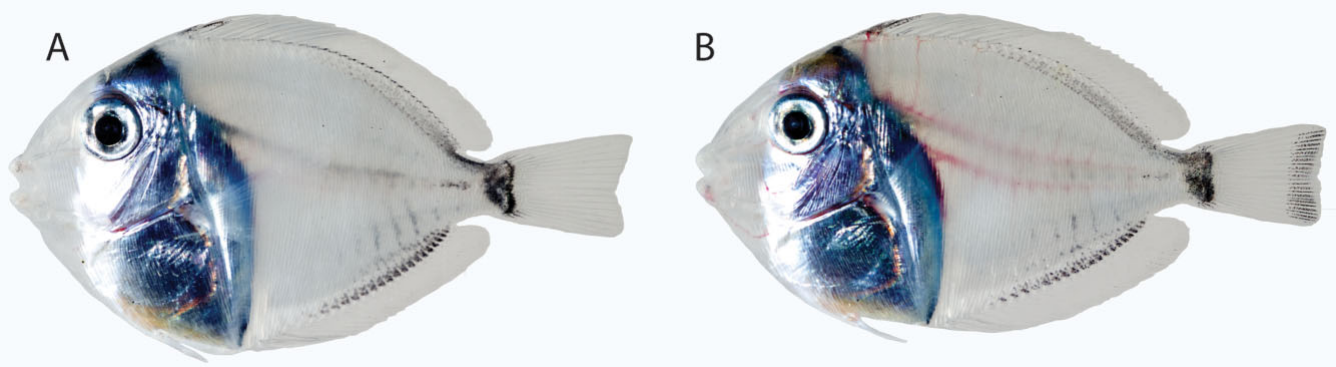
Percomorphacea (Acanthuriformes). A, *Acanthurus bahianus*, 26 mm Standard Length (SL), BLZ 8441. B, *Acanthurus chirurgus*, 28 mm SL, BLZ 8442. Note: the internal red coloration behind the head is associated with the circulatory system, not chromatophores. Photos by Julie Mounts and David Smith.

**Figure 15:**
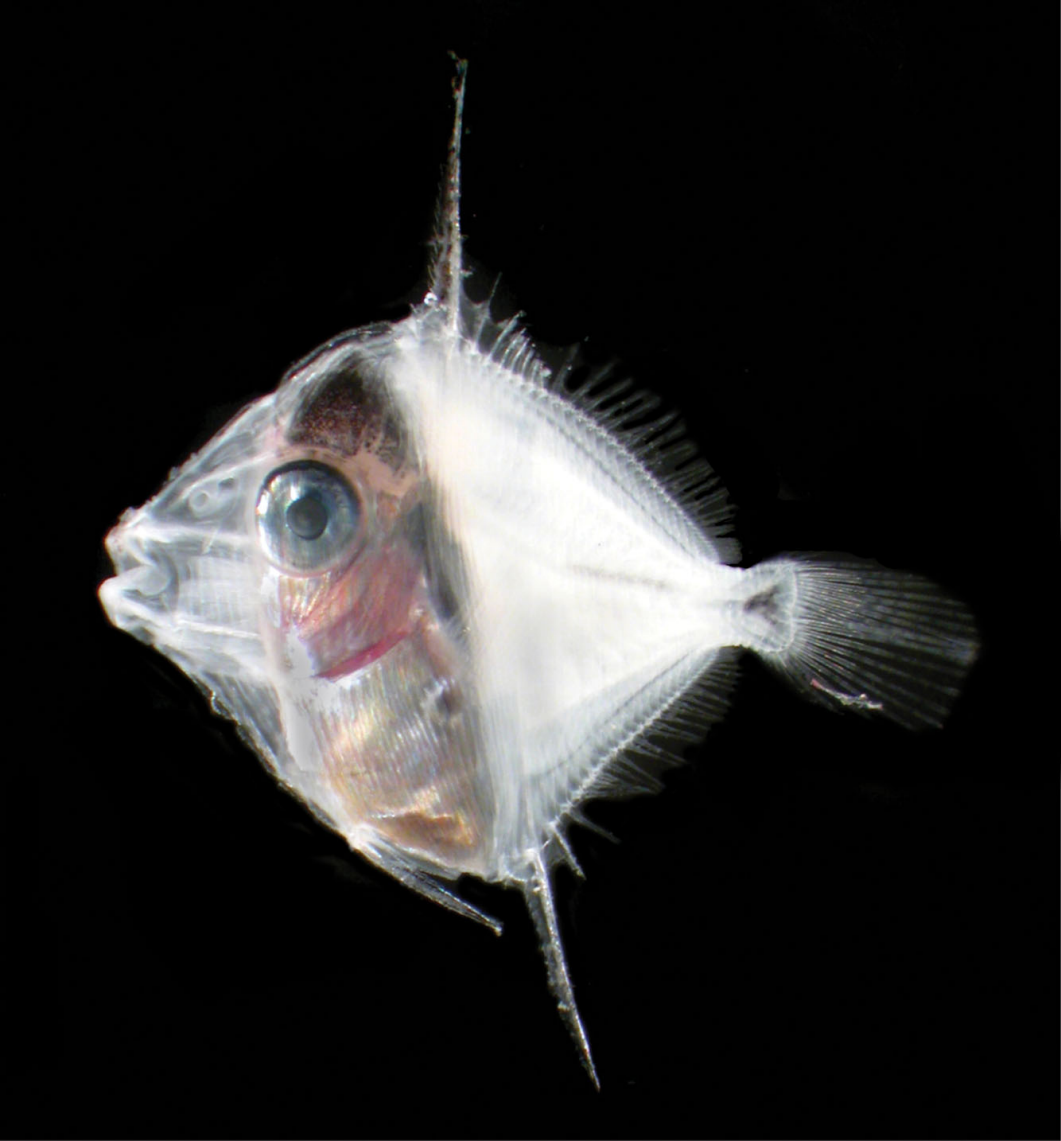
Percomorphacea (Acanthuriformes). *Acanthurus* sp., Florida Straits. Photo by Cedric Guigand.

Acanthuriformes are represented among the larvae examined by two species of *Acanthurus* surgeonfishes from Belize (Fig. 14) and an earlier stage of an unidentified species of *Acanthurus* from the Florida Straits (Fig. 15). The specialized, pelagic ‘acronurus’ larval stage (e.g. Leis & Richards, 2004) is mostly transparent with a band of silver- or blue-reflecting iridophores from the dorsum to the pelvis that encompasses the orbit and gut. There is little if any orange or yellow pigment in the acronurus stage, but an image of preflexion larvae of *Acanthurus mata* (Connell, 2007; Appendix) shows xanthophores on the upper jaw and an internal streak of xanthophores beneath the anterior section of the notochord. Relationships of acanthuriforms are unclear, but a close relationship with tetraodontiforms has been proposed (e.g. Tyler, 1980; Rosen, 1984). Tetraodontiform larvae (see ‘Tetraodontiformes’ below) typically have iridophores on the gut and sometimes over much of the body, but they are otherwise very different from acanthuriforms in having much more colour (xanthophores or bronze iridophores), melanophores (sometimes in striking patterns), or both.

#### Blenniiformes

**Figure 16:**
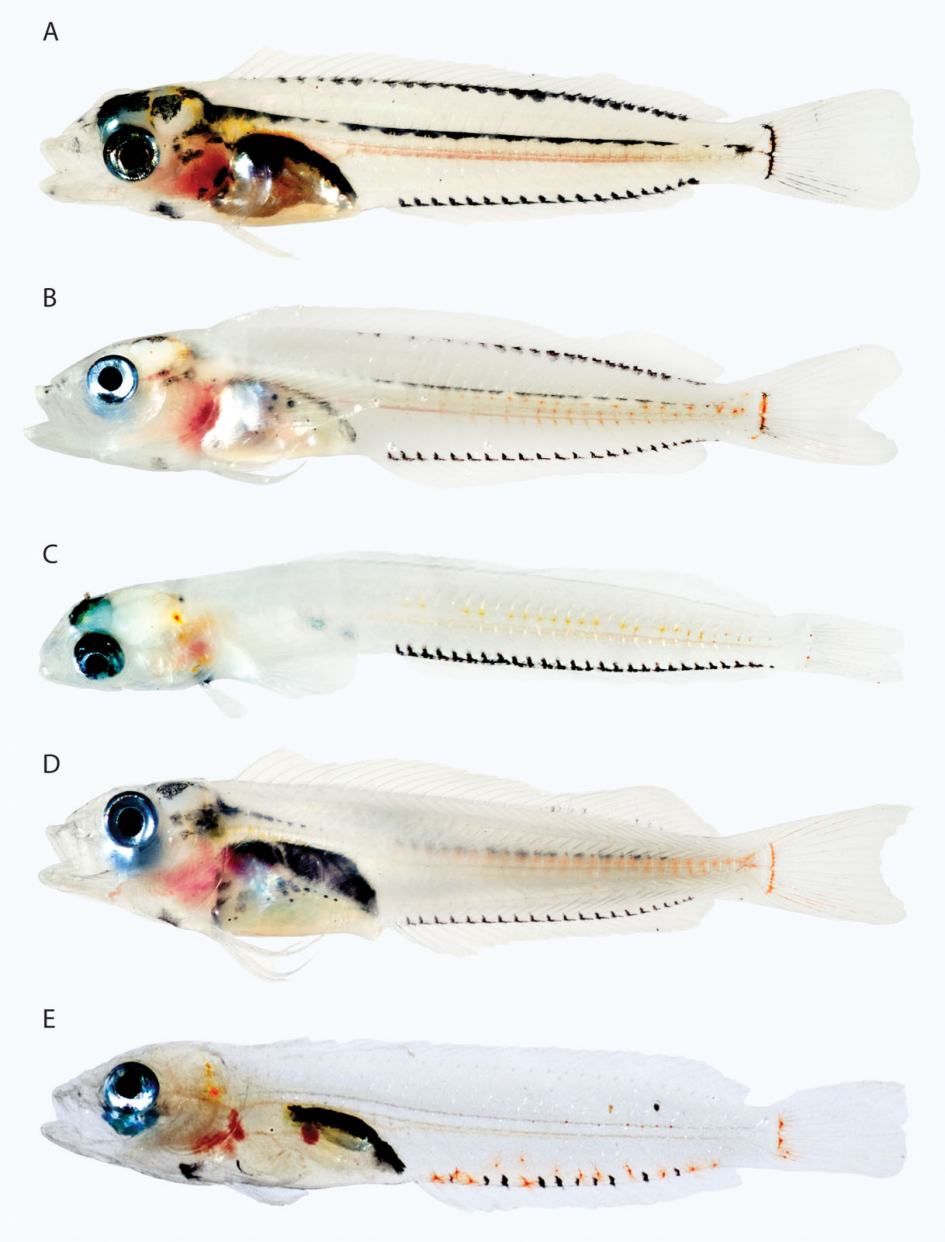
Percomorphacea (Blenniiformes). A, *Labrisomus cricota*, 21.0 mm Standard Length (SL), BLZ 7006. B, *Labrisomus bucciferus*, 19.0 mm SL, BLZ 7253. C, *Acanthemblemaria greenfieldi*, 13.0 mm SL, BLZ 6386. D, *Malacoctenus triangulatus*, 18.0 mm SL, BLZ 8439. E, *Paraclinus fasciatus*, length not recorded, BLZ 6071. Note: in all images, internal red coloration in the thoracic cavity is associated with the circulatory system, not chromatophores. Photos A–D by Julie Mounts and David Smith; E by Lee Weigt and Carole Baldwin.

Blenniiformes are represented in the Belize material by larval chaenopsids and labrisomids. Larvae of both families have an elongate body and share the following chromatophore pattern: melanophores on the ventral midline associated with the anal-fin base and internally along the dorsal margin of the vertebral column, orange or yellow pigment on the temporal region of the head, internal orange pigment along the vertebral column, and orange chromatophores at the base of the caudal fin (Fig. 16). Colour patterns vary little among some genera (e.g. *Acanthemblemaria* of the Chaenopsidae, *Labrisomus* of the Labrisomidae – Fig. 16A–D), but the single larval specimen of *Paraclinus* (Labrisomidae) differs in having prominent orange chromatophores mixed with the melanophores along the anal-fin base (Fig. 16E). Additional *Paraclinus* species are needed to determine whether or not this is a generic pattern. The chromatophore pattern on the body could not be interpreted well in an *in situ* image of a larval fish from Hawaii identified by G. D. Johnson (pers. comm.) as a blenniid (image not reproduced here), but the fish is unlike other blennioids in having enlarged pectoral fins with xanthophores on the membranes between fin elements.

#### Caproiformes

Caproiformes are represented in the images examined only by preflexion *Antigonia rubescens* from South Africa (Connell, 2007; Appendix). Recently hatched larvae have xanthophores on the body, but no information is available for larger specimens.

#### Carangiformes

**Figure 17:**
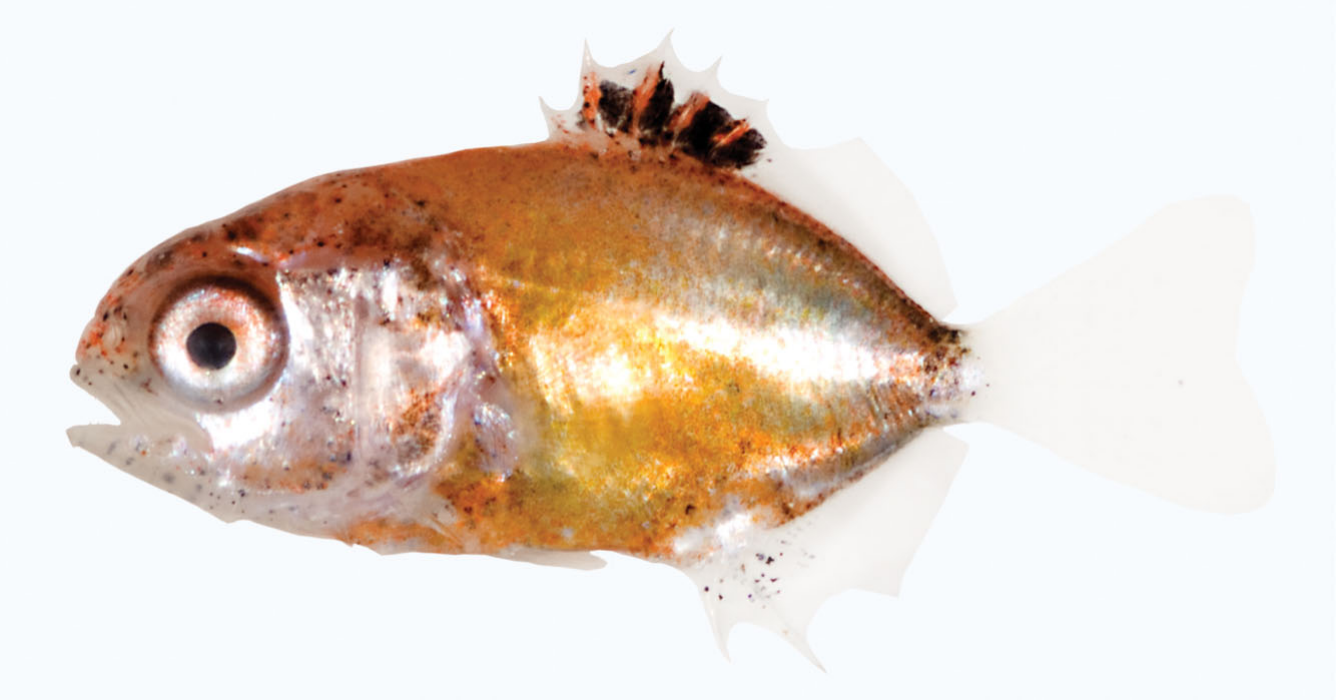
Percomorphacea (Carangiformes). *Trachinotus falcatus*, 8.0 mm Standard Length, BLZ 5428. Photo by Lee Weigt and David Smith.

**Figure 18:**
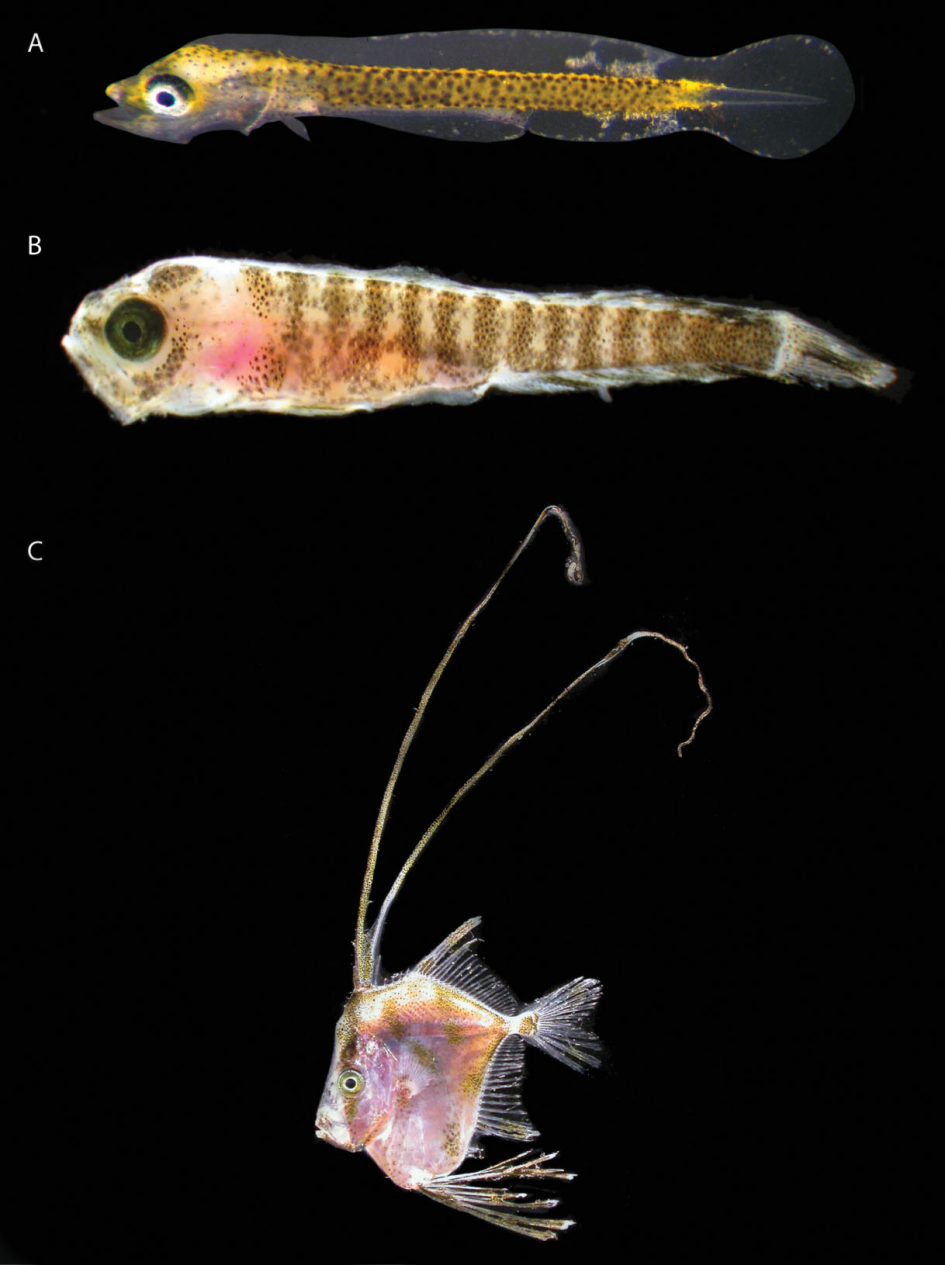
Percomorphacea (Carangiformes). A, *Coryphaena hippurus*, South Africa, photo by Allan Connell. B, *C. hippurus*. C, *Selene vomer*. B and C, Florida Straits, photos by Cedric Guigand, previously published in Fogarty & Botsford (2007) and Cowen *et al*. (2007), respectively. Note: the internal pink coloration in the thoracic region of B is associated with the circulatory system, not chromatophores.

Carangiformes are not common in larval-fish collections off Belize, but an 8.0-mm SL *Trachinotus* (Carangidae) has most of the body covered with orange chromatophores and silver-reflecting iridophores (Fig. 17). A colour image of a young carangid, *Selene* (Fig. 18C), shows yellow/orange chromatophores on a somewhat silvery body as well as on most fins. A 5.1-mm preflexion larva of the coryphaenid, *Coryphaena hippurus*, has xanthophores mixed with melanophores from the tip of the snout posteriorly to the base of the incipient caudal fin (Fig. 18A). A postflexion *Coryphaena hippurus* larva (Fig. 18B) and a reared postflexion rachycentrid, *Rachycentron canadum* (D. Benetti, pers. comm.), have some yellow pigment mixed with melanophores on the body, but the solid yellow ground coloration present in preflexion *Coryphaena* is absent. Colour patterns are not known for other carangiforms (Nematistiidae, Echeneididae), and no conclusions can be drawn regarding the phylogenetic significance of colour for the order or for families, genera, and species within. The bars of pigment in postflexion *Coryphaena* (Fig. 18B) resemble those of *Selene* in having some yellowish coloration mixed with melanophores, and this character could be significant at the ordinal level. Phylogenetic analysis of both mitochondrial (Miya, Satoh & Nishida, 2005) and nuclear (Little, Lougheed & Moyes, 2010) data suggested a close relationship between carangiforms and pleuronectiforms, but there is nothing obvious in the colour patterns of larvae to support this (see ‘Pleuronectiformes’ below). Nuclear DNA data have also suggested a relationship between carangids and istiophorids (Little *et al*., 2010), which is discussed under ‘Scombriformes’ below.

#### Gobiesociformes

**Figure 19:**
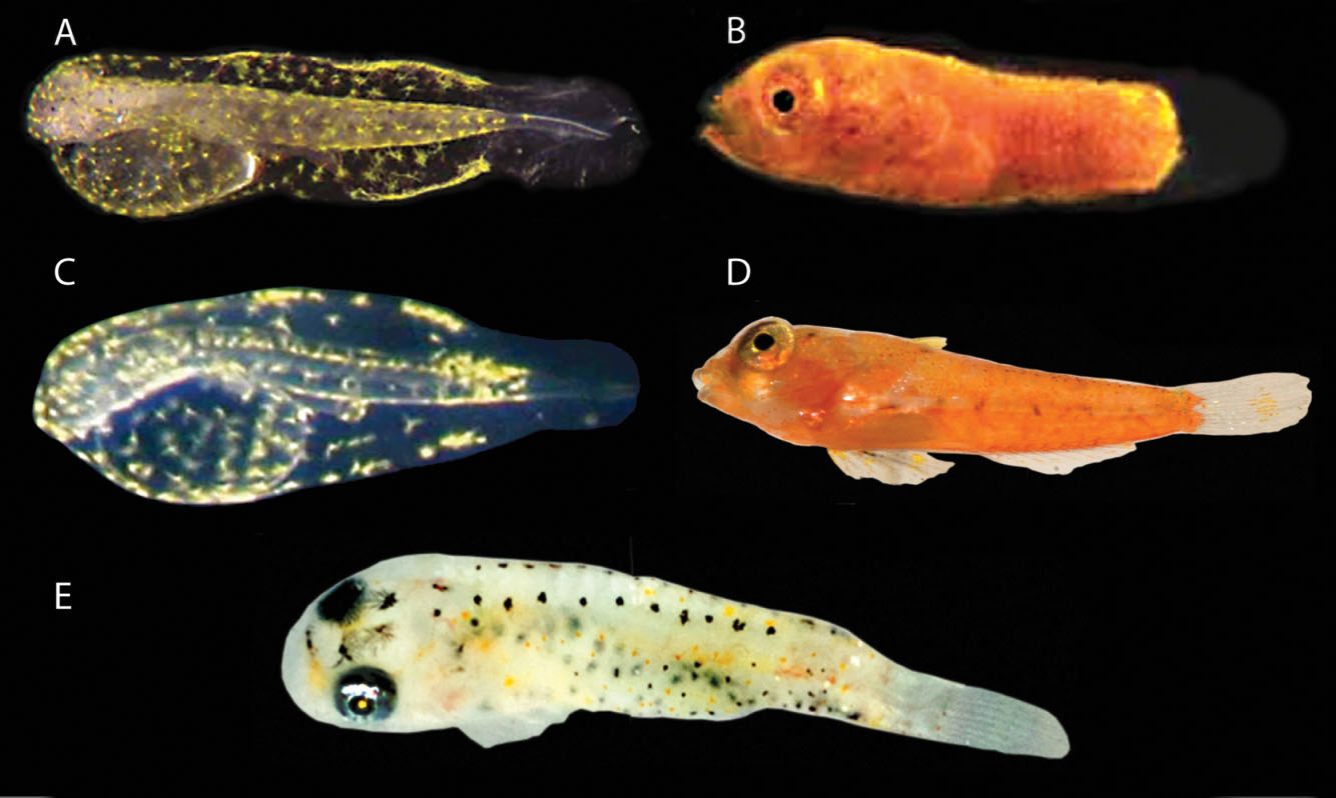
Percomorphacea (Gobiesociformes). A, B, *Synchiropus splendidus*, reared specimens. C, *Callionymus marleyi*, South Africa. D. *Callionymus bairdi*, 7.0 mm Standard Length (SL), BLZ 4315. E, *Acyrtops beryllinus*, 4.0 mm SL, BLZ 6231. Photos A, B by Matthew Wittenrich; C by Allan Connell; D by Lee Weigt and David Smith; E by Julie Mounts and David Smith.

Gobiesociformes comprise the Gobiesocidae, Callionymidae, and Draconettidae. Colour information in larvae is available for several callionymids and one gobiesocid. Wittenrich *et al*. (2010) described morphological development of laboratory-reared *Synchiropus splendidus* and noted a complete colour transition from yellow in early-stage larvae to nearly solid orange at 8 days posthatching (Fig. 19A, B). Connell (2007) provided a colour image of a 1.8-mm NL larva of *Callionymus* sp. from South Africa (Fig. 9C) and one of a 1.6-mm NL larva of *Draculo* (Appendix) that are nearly identical to the similar-sized (2 days posthatching) larva of *Syn. splendidus* (Fig. 19A) in having the head and body covered in prominent xanthophores except for the posterior fin fold. The colour pattern in larger larvae of the South African *Callionymus* is unknown, but postflexion larvae of *Callionymus bairdi* from Belize (Fig. 19D) are nearly solid orange like *Synchiropus*. Additional material is needed to determine whether the observed yellow-to-orange colour transition is diagnostic of callionymids or if it is also present in other gobiesociform families. A 4.0-mm SL larva of the gobiesocid *Acyrtops* from Belize has yellow/orange coloration on the head and scattered over most of the body (Fig. 19E), possibly a very similar pattern as in the callionymids but with the erythrophores contracted. Larger and smaller specimens of these taxa are not available. The only other percomorph larvae examined that were as extensively covered by erythrophores as postflexion callionymid larvae are some apogonids and the goby *Priolepis* (see ‘Perciformes’ and ‘Gobiiformes’ below)*. Apogon aurolineatus* is particularly similar to *Callionymus bairdi* in having a bright orange body with yellow first dorsal and pelvic fins. The presence of similar chromatophore patterns in these presumably distantly related taxa is best interpreted as convergence.

Pigment in larvae does not appear to provide any evidence for a close relationship between gobiesociforms and blenniiforms as proposed by Miya *et al*. (2003) and Springer & Orrell (2004). The extensive coverage of yellow/orange pigment in the gobiesociforms examined is very different from the more restrictive colour pattern observed in blenniiforms; however, information on colour is needed for larvae of more taxa, especially those blenniiforms hypothesized by Stepien *et al*. (1997) to be more basal members of the group than labrisomids and chaenopsids.

#### Gobiiformes

Gobiiformes examined include members of the Gobiidae, Eleotrididae, and Microdesmidae. Larvae of all exhibit erythrophores or xanthophores, or both, and some have iridophores, but there are no obvious diagnostic patterns for the order, major clades within the order, or the highly diverse Gobiidae. Preliminary comparative data suggest that colour patterns may be most informative at intergeneric, generic, and species levels.

**Figure 20:**
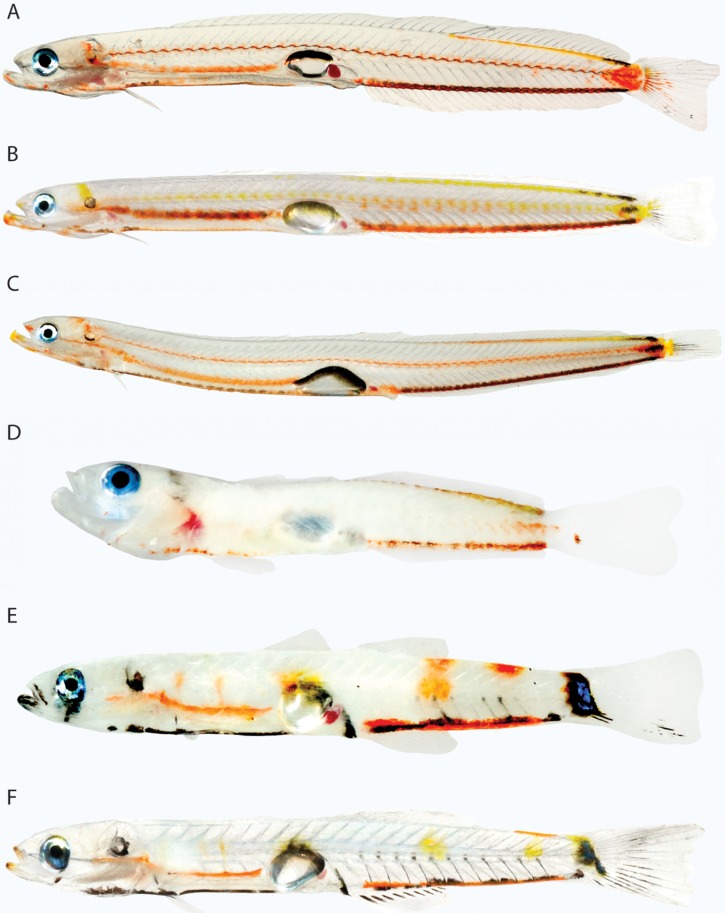
Percomorphacea (Gobiiformes). A, *Cerdale floridana*, 21.0 mm Standard Length (SL), Belize. B, *Microdesmus bahianus*, 17.5 mm SL, Belize. C, *Microdesmus carri*, 22 mm SL, BLZ 8322. D. *Ptereleotris helenae*, 12.0 mm SL, BLZ 7323. E, *Eleotris pisonis*, 13.0 mm SL, BLZ 7101. F, *Erotelis smaragdus*, 13 mm SL, Belize. Note: the internal red coloration in the thoracic region of *P. helenae* is associated with the circulatory system, not chromatophores. Photos A, B, F by Julie Mounts and Carole Baldwin; C by Lee Weigt and Carole Baldwin; D, E by Julie Mounts and David Smith.

Larvae of the microdesmid genera *Microdesmus* and *Cerdale* have nearly identical patterns of orange/yellow chromatophores (Fig. 20A–C). Hoese (1984) united *Microdesmus*, *Cerdale*, and three other microdesmid genera with *Ptereleotris* and its allies in an expanded family Microdesmidae, but Smith & Thacker (2000) recognized the Microdesmidae and Ptereleotrididae as separate families. Thacker's (2003, 2009) molecular phylogenetic analyses did not recover a monophyletic group comprising microdesmids and ptereleotridids, but the molecular analysis of Thacker & Roje (2011) did, with ptereleotridids paraphyletic with respect to microdesmids. Superficially, the larval *Ptereleotris* (Fig. 20D) is quite different from larval *Microdesmus* and *Cerdale* in that it does not have an elongate body with an obvious series of erythrophores internally along the vertebral column and above the gut. The available colour images of *Ptereleotris*, however, are of specimens that are mostly opaque (vs. transparent), and although internal erythrophores are present above the gut and along the lateral midline posteriorly, it is not possible to determine the extent of these series. Colour patterns in the three genera are otherwise quite similar, with all having a series of erythrophores along the ventral midline (except directly beneath the swim bladder), orange or yellow chromatophores in a series along the dorsal midline posteriorly, and at least some orange pigment externally on the central portion of the caudal peduncle. This pattern was not observed in other gobiiforms, and may provide corroborative evidence for a monophyletic group comprising the two families. The hypothesis that *Coryphopterus* gobies are more closely related to *Microdesmus* and *Cerdale* than microdesmids are to *Ptereleotris* (Thacker, 2003) would not appear to be supported by colour patterns in larvae. *Coryphopterus* gobies have a distinctive pattern of erythrophores on the trunk and lack the chromatophore pattern described above for microdesmids (see last paragraph of Gobiiformes section, below). *Coryphopterus* shares with microdesmids the presence of erythrophores mixed with melanophores along the anal-fin base, but this pigment is also present in *Ptereleotris* and even the distantly related eleotridids.

Miller (1998) synonymized the eleotridid genera *Eleotris* and *Erotelis*, and colour patterns in larvae of the two genera are strikingly similar and distinctive among gobiiforms (Fig. 20E, F). Thacker's (2003) molecular analysis did not recover the two as a monophyletic group; rather, they are successive sister groups to all other gobiiforms excluding the Odontobutidae. The more comprehensive molecular phylogeny of Thacker (2009) corroborates a common ancestry for the two genera, with *Erotelis* embedded within the *Eleotris* clade. In addition to having similar patterns of erythrophores/xanthophores, *Eleotris* and *Erotelis* are the only two gobiiform larvae examined that have a swath of blue-reflecting iridophores at the base of the caudal fin.

**Figure 21:**
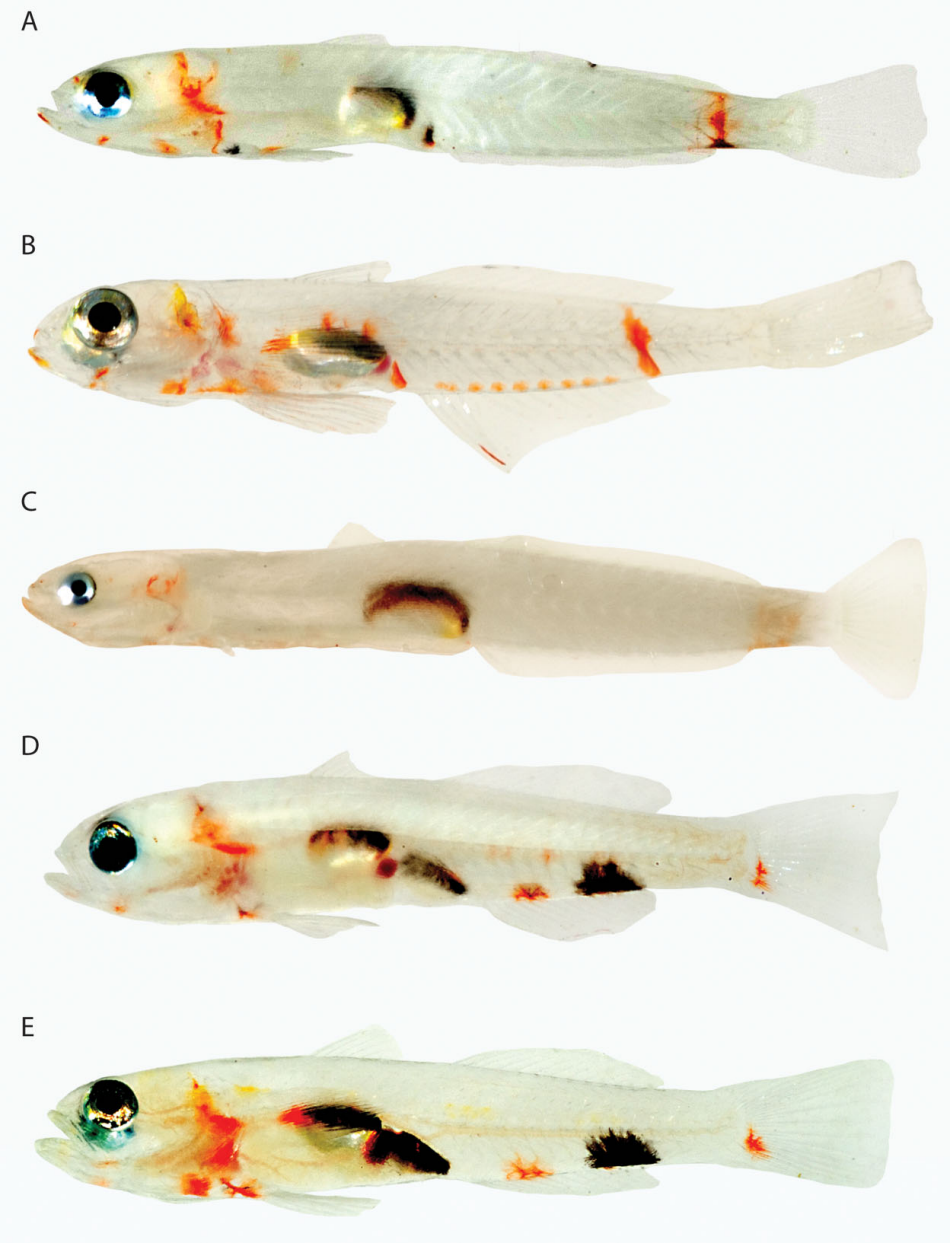
Percomorphacea (Gobiiformes). A, *Ctenogobius saepepallens*, 10.0 mm Standard Length (SL), BLZ 7387. B, *Gnatholepis thompsoni*, 11.0 mm SL, Belize. C, *Gobionellus oceanicus*, 12.0 mm SL, BLZ 5473. D, *Nes longus*, 12.0 mm SL, BLZ 7183. E, *Psilotris* sp., 12.0 mm SL, BLZ 7187. Photo A by Lee Weigt and Carole Baldwin; B by Julie Mounts and Carole Baldwin; C by Lee Weigt and David Smith; D, E by Julie Mounts and David Smith.

Morphological and molecular data support a close relationship between *Ctenogobius* and *Gnatholepis* (Harrison, 1989; Thacker, 2003, 2009). As noted by Baldwin & Smith (2003), larval *Ctenogobius saepepallens* and *Gnatholepis thompsoni* are similar in having a prominent, narrow bar of orange pigment on the body just posterior to the anal-fin base that is lacking in other gobiids (Fig. 21A, B). Baldwin & Smith (2003) also noted that the orientation of this bar is slightly different in the two species and that it may not be homologous. *Gobionellus oceanicus* has a wide bar of pale orange pigment on the caudal peduncle that, because of the long anal fin in this species, is located at the posterior base of the anal fin as the narrow bar is in *Ctenogobius* and *Gnatholepis* (Fig. 21C). The phylogenetic significance within the Gobiidae of orange pigment at the posterior base of the anal fin extending dorsally from the ventral midline is unclear. Observations of colour patterns in larvae of more genera of *Gobionellus-*group gobies (Birdsong, Murdy & Pezold, 1988) is needed, including *Evorthodus*, which Thacker (2003, 2009) proposed as the sister group of *Ctenogobius*. Postlarval *Ctenogobius boleosoma* (Wyanski & Targett, 2000) shares with *Ct. saepepallens* an erythrophore at the tip of the lower jaw, a vertical reddish-orange streak between the thoracic and abdominal regions, and the orange bar posterior to the anal-fin base. Identification of other *Ctenogobius* larvae is needed, but the combination of these pigment characters may be diagnostic of the genus.

*Nes* and *Psilotris* are seven-spine gobies of the ‘*Gobiosoma* group’ (Birdsong *et al*., 1988) that share with *Varicus*, *Chriolepis*, and *Gobulus* the absence of head pores (Böhlke & Robins, 1968). Rüber, Van Tassell & Zardoya (2003) recovered this group as monophyletic, minus *Varicus*, which was not included in their analysis. As noted by Smith & Baldwin (1999), larvae of *Nes* and *Psilotris* are so similar that at first these authors did not recognize them as distinct taxa. The chromatophore pattern is nearly identical in the two genera and distinctive among gobiids examined (Fig. 21D, E). Identification of additional goby larvae, including *Gobulus* and *Chriolepis*, is needed to determine whether the pattern is unique to *Nes* and *Psilotris* or perhaps to a larger group. Rüber *et al*. (2003) hypothesized that the relationships of *Nes* are as follows: (*Nes*(*Gobulus*(*Chriolepis* + *Psilotris*))).

**Figure 22:**
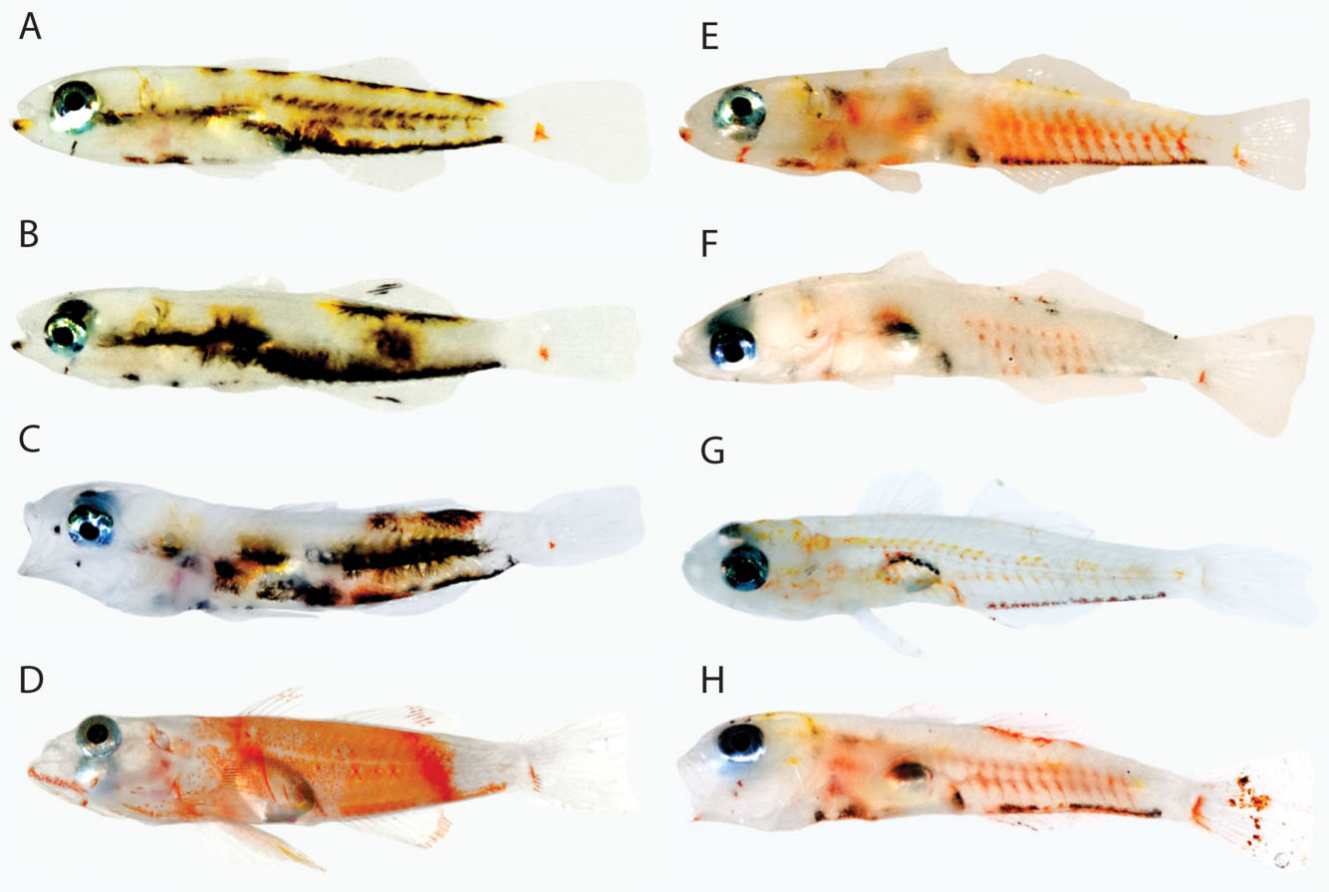
Percomorphacea (Gobiiformes). A, *Bathygobius curacao*, 5.5 mm Standard Length (SL), BLZ 7305. B, *Bathygobius lacertus*, 6.0 mm SL, BLZ 7370. C, *Bathygobius soporator*, 6.0 mm SL, BLZ 6072. D, *Priolepis hipoliti*, 10.0 mm SL, Belize. E, *Coryphopterus kuna*, 7.5 mm SL, BLZ 5134. F, *Coryphopterus tortugae*, 7.0 mm SL, BLZ 5227. G, *Coryphopterus personatus*, 8.0 mm SL, BLZ 10007. H, *Coryphopterus venezuelae*, 8.5 mm SL, BLZ 5392. Photos A, B by Julie Mounts and David Smith; C by Lee Weigt and Carole Baldwin; D−F, H by Julie Mounts and Carole Baldwin; G by Donald Griswold and Carole Baldwin.

At the generic level, colour patterns in larvae are useful in diagnosing *Bathygobius* and *Coryphopterus. Bathygobius* larvae have orange/yellow chromatophores on the dorsal and ventral portions of the trunk (in association with melanophores) that extend toward, and often meet at, the lateral midline (Fig. 22A–C). Although the colour (yellow or orange) and density of pigment differ among species, the net effect is distinctive (Baldwin & Smith, 2003; Tornabene *et al*., 2010). All known *Coryphopterus* larvae have diagonal bars of orange pigment on the trunk, the height of the bars and how far the series extends anteriorly varying among species (Baldwin & Smith, 2003; Fig. 22E–H). Colour patterns in larvae of genera hypothesized to be closest to *Bathygobius* (*Glossogobius*, *Istigobius*, and *Callogobius*; Tornabene & Pezold, 2011, and references therein; Thacker, 2009) and *Coryphopterus* (*Lophogobius*; Thacker, 2003, 2009) are unknown. The unique generic patterns described above could diagnose larger groups. Monophyly of Thacker's (2003) clade IIIA, which includes *Bathygobius* and *Priolepis*, would not appear to be supported by larval colour patterns. Larval *Priolepis* (Fig. 22D) is distinctively orange.

#### Labriformes

**Figure 23:**
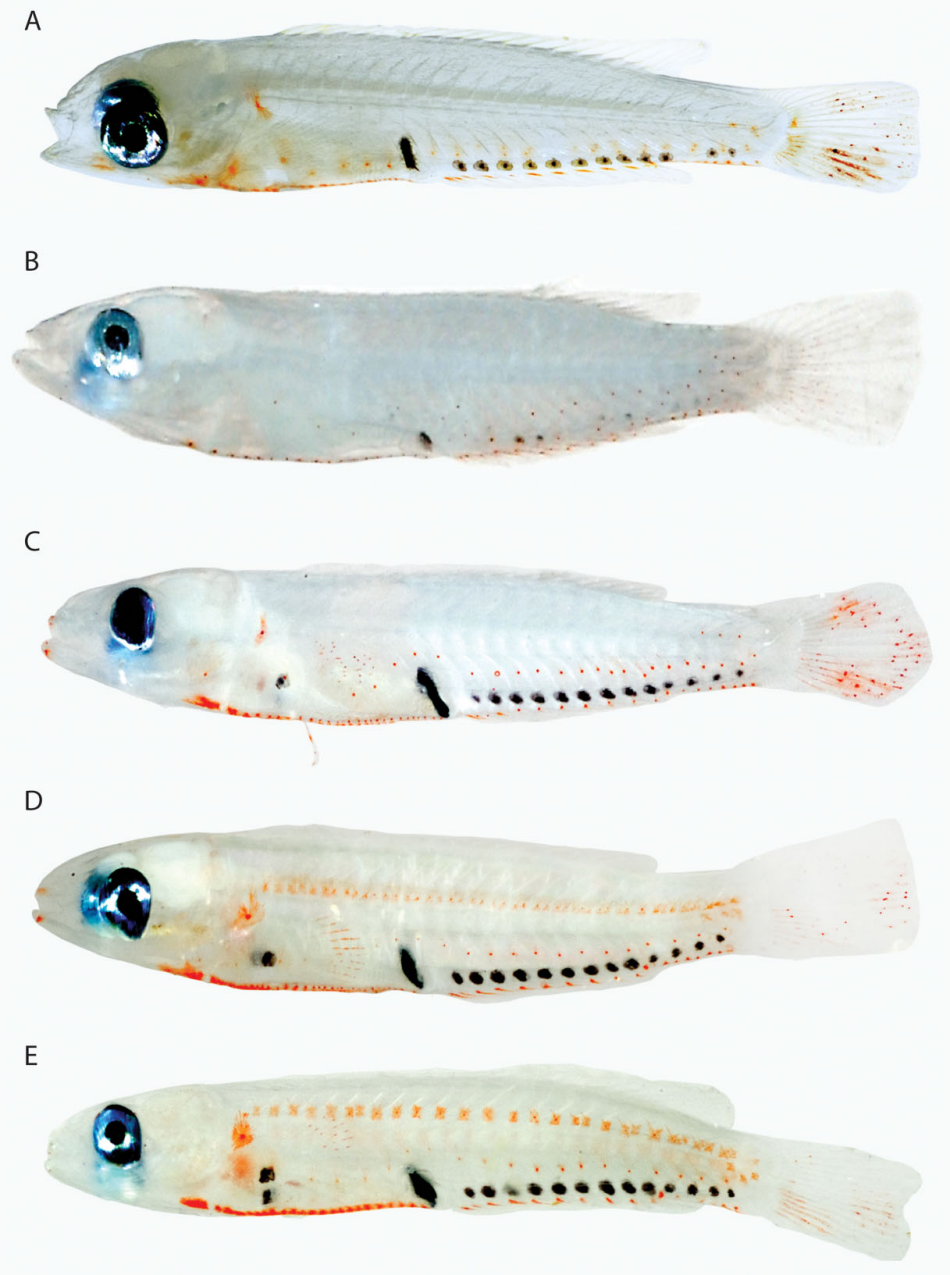
Percomorphacea (Labriformes). A, *Cryptotomus roseus*, 9.0 mm Standard Length (SL), BLZ 10005. B, *Scarus iseri*, 7 mm SL, Belize. C, *Sparisoma atomarium*, 10.0 mm SL, BLZ 7312. D, *Sparisoma chrysopterum*, 9.0 mm SL, BLZ 6383. E, *Sparisoma radians*, 11.0 mm SL, BLZ 7289. Photo A by Donald Griswold and Carole Baldwin; B by Julie Mounts and Carole Baldwin; C−E by Julie Mounts and David Smith.

Labriformes comprise the Cichlidae, Embiotocidae, Labridae, Odacidae, Pomacentridae, and Scaridae. The monophyly of the group is questionable (Wiley & Johnson, 2009, and references therein), and molecular data suggest that scarids are embedded within the Labridae (Westneat & Alfaro, 2005; Choat *et al*., 2012). Marine labriforms for which colour patterns were assessed for larvae are pomacentrids, labrids, and scarids. All exhibit nonmelanistic chromatophores, usually erythrophores, but the patterns are not diagnostic of the order. Based on existing comparative material, colour patterns appear useful in diagnosing some families and genera. All scarid larvae examined are united in having a linear series of erythrophores along the ventral midline of the trunk from beneath the operculum to the anus, a linear series of erythrophores along the anal-fin base, a roughly linear series of erythrophores above the anal-fin base that curves dorsally on the caudal peduncle where it is continuous with erythrophores on the lateral midline, and erythrophores on the caudal fin (Fig. 23). This pattern is present in *Cryptotomus*, *Scarus*, and *Sparisoma*, which differ from one another in (1) the extent of orange coloration on the caudal fin (with streaks of bright orange chromatophores on the ventral lobe in *Cryptotomus*, Fig. 23A, vs. orange pigment more scattered or paler in the other genera); (2) organization of erythrophores and melanophores above the anal-fin base (somewhat haphazard in *Scarus –* Fig. 23B, linear in *Cryptotomus* and *Sparisoma –* Fig. 23A, C, D); and (3) the configuration of erythrophores along the ventral midline on the anterior portion of the trunk (forming an almost continuous line of orange pigment in *Cryptotomus* and *Sparisoma*, more widely spaced in *Scarus*). A generic-level phylogeny of scarids presented by Kazancioğlu *et al*. (2009) suggests that *Cryptotomus* and *Sparisoma* are sister groups, and (2) and (3) above, if apomorphic, would lend extra support to this hypothesis. The anterior extension of the midlateral series of erythrophores almost to the head in *Sparisoma atomarium* (Fig. 23D) and *Sparisoma radians* (Fig. 23E) is unique among labriforms and may indicate that these species are more closely related to one another than either is to *Sparisoma chrysopterum* (Fig. 21C).

**Figure 24:**
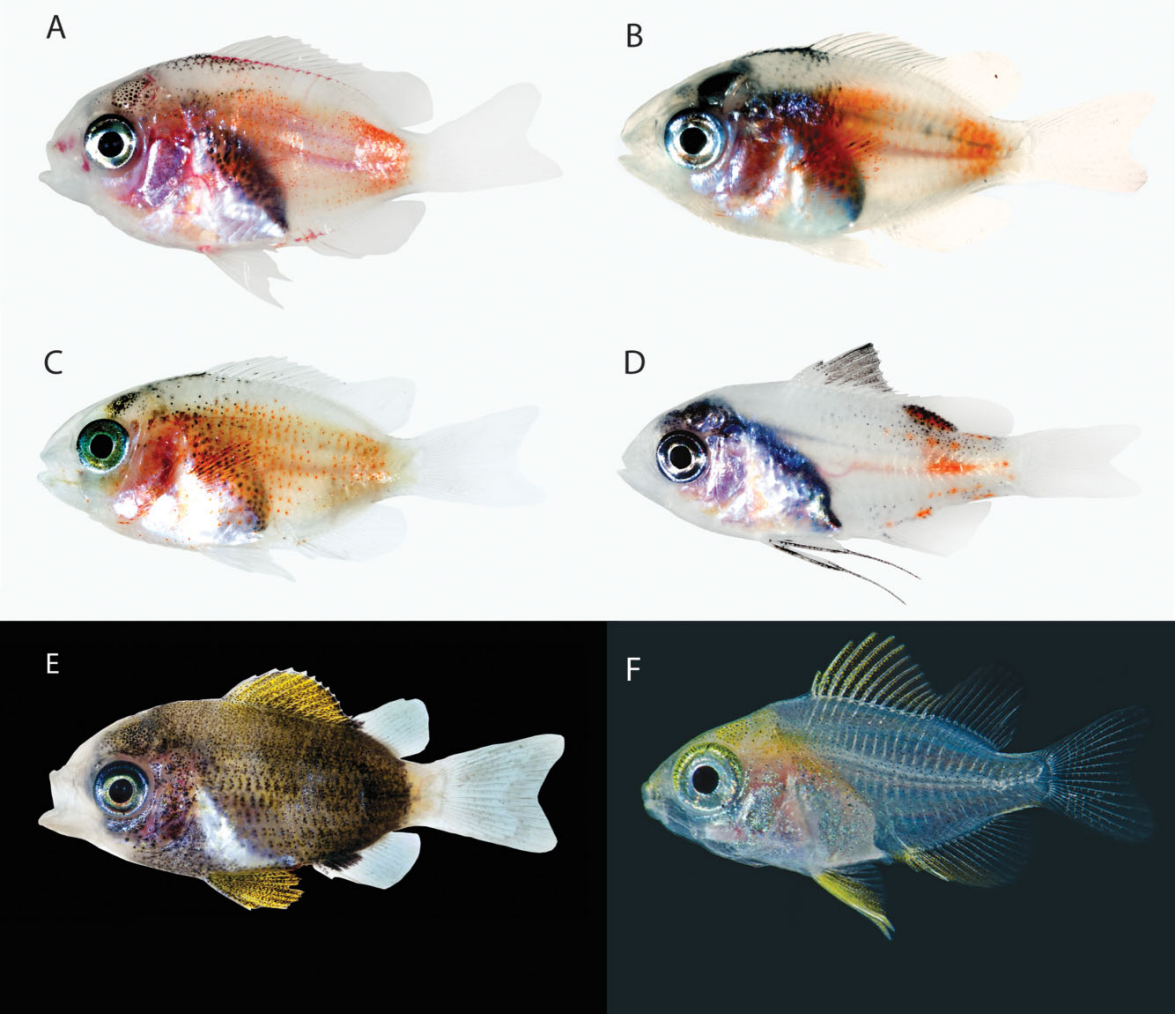
Percomorphacea (Labriformes). A, *Stegastes partitus*, 11.5 mm Standard Length (SL), BLZ 8454. B, *Stegastes variabilis*, 10.0 mm SL, BLZ 4523. C, *Stegastes planifrons*, 10.0 mm SL, BLZ 6008. D, *Chromis cyanea*, 14.0 mm SL, BLZ 8451. E, *Abudefduf saxatilis*, 10.0 mm SL, BLZ 10214. F, *Amblyglyphidodon ternatensis*, reared aquarium specimen. Photos A, D by Julie Mounts and David Smith; B by Lee Weigt and David Smith; C by Lee Weigt and Carole Baldwin; E by Donald Griswold and Carole Baldwin; F by Matthew Wittenrich.

Larval pomacentrids examined have silvery iridophores on the gut and at least some erythrophores or xanthophores on the trunk and fins (Fig. 24). Colour patterns of larvae of the four genera examined are distinctive. *Stegastes* larvae have a swathe of orange pigment on the trunk from just behind the eye to the anterior-most part of the caudal peduncle, where the swath ends abruptly in a near vertical line (Fig. 24A–C). Differences among *Stegastes* species include the presence or absence of erythrophores on the pectoral fin and along the spinous dorsal-, pelvic-, and anal-fin bases. Colour information was available for only one species of *Chromis* (Fig. 24D) and one of *Abudefduf* (Fig. 24E), but patterns in these genera are distinct from one another and from *Stegastes*. *Chromis* lacks colour on the fins and has erythrophores restricted to the posterior portion of the trunk and caudal peduncle, whereas *Abudefduf* is distinctive in having xanthophores on most of the trunk (mixed with melanophores) and conspicuously yellow first dorsal and pelvic fins. Like *Stegastes*, pigment on the trunk in *Abudefduf* ends abruptly on the anterior portion of the caudal peduncle. Reared larvae of *Amblyglyphidodon ternatensis* (Fig. 24F) are similar to *Abudefduf* in having prominent yellow pigment on the dorsal and pelvic fins. They are distinctive in having yellow pigment covering the dorsal portion of the head, including the dorsal portion of the orbit, and also the anterior portion of the anal fin. The molecular phylogeny of Cooper, Smith & Westneat (2009) suggests that *Stegastes*, *Chromis*, *Abudefduf*, and *Amblyglyphidodon* are members of four distinct evolutionary assemblages (Stegastinae, Chrominae, Abudefdufinae, Pomacentrinae, respectively), a hypothesis that is not contradicted by colour patterns in larvae. Larval *Abudefduf* and *Amblyglyphidodon* are the most similar of the four in terms of coloration, and Abudefdufinae and Pomacentrinae are sister groups according to Cooper *et al*. (2009). Acquisition of colour information for larvae of additional pomacentrids is needed to determine whether or not the colour patterns identified herein characterize the subfamilies (or some subset of them) and whether aspects of the colour pattern in *Abudefduf* and *Amblyglyphidodon* represent a synapomorphy of Abudefdufinae and Pomacentrinae.

**Figure 25:**
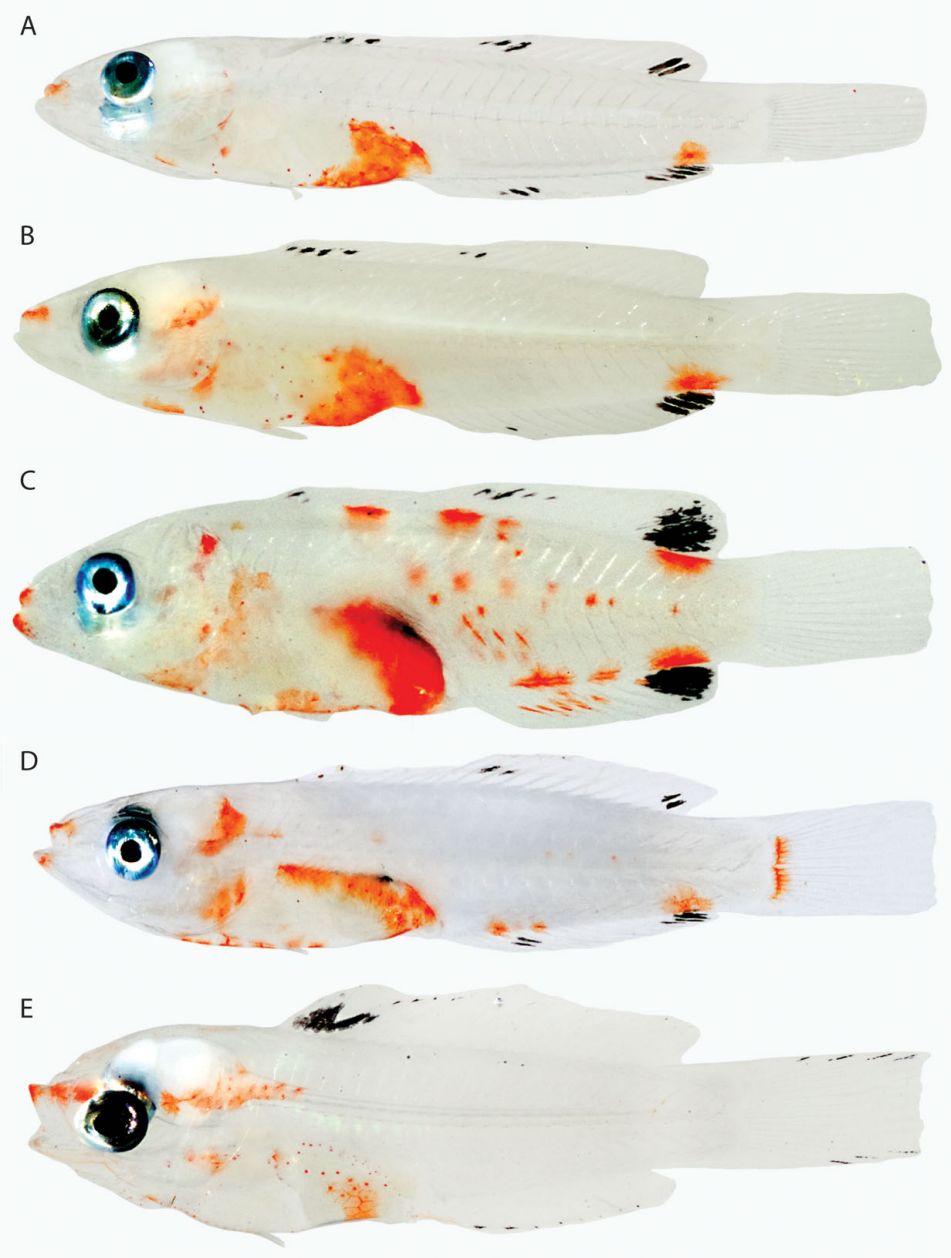
Percomorphacea (Labriformes). A, *Halichoeres bivittatus*, 12.5 mm Standard Length (SL), BLZ 6426. B, *Halichoeres garnoti*, 15.0 mm SL, BLZ 7085. C, *Halichoeres maculipinna*, 17.0 mm SL, BLZ 7124. D, *Halichoeres poeyi*, 14.0 mm SL, BLZ 6102. E, *Thalassoma bifasciatum*, 11.0 mm SL, BLZ 8334. Photos A−C by Julie Mounts and David Smith; D, E by Lee Weigt and Carole Baldwin.

All genera of labrid larvae examined (*Anampses*, *Halichoeres*, *Lachnolaimus*, *Thalassoma*, *Xyrichtys*) except *Doratonotus* have prominent orange pigment on the tip of the upper jaw and usually also on the tip of the lower jaw (Fig. 25). Otherwise, colour patterns are distinctive among genera or groups of genera. *Anampses* (A. Connell, pers. comm.), *Halichoeres* (Fig. 25A–D), and *Thalassoma* (Fig. 25E) larvae have at least a small cap and sometimes a broader covering of erythrophores on the gut and orange spots or blotches on the posterior portion of the head. *Anampses* and *Halichoeres* also have an orange blotch on the ventral portion of the trunk just posterior to the anal-fin base. Species-specific features within *Halichoeres* include the presence of several orange blotches on the dorsal and mid-lateral portions of the trunk (*Halichoeres maculipinna*, Fig. 25C) and presence of a vertical line of erythrophores on the caudal-fin base (*Halichoeres poeyi*, Fig. 25D). Colour patterns in *Halichoeres bivittatus* (Fig. 25A) and *Halichoeres garnoti* (Fig. 25B) are extremely similar, but these species can be separated by the pattern of melanophores on the dorsal and anal fins. Rocha, Pinheiro & Gasparini (2010) presented a preliminary molecular phylogeny of New World *Halichoeres*, a relevant aspect of which is the placement of *Ha. maculipinna* in a clade distinct from one comprising *Ha. poeyi*, *Ha. garnoti*, *Ha. bivittatus*, and several other *Halichoeres* species (Rocha *et al*., 2010: fig. 4). Larvae of *H. maculipinna* differ from those of *Ha. poeyi*, *Ha. garnoti*, and *Ha. bivittatus* in having much more orange coloration on the body and more prominent black blotches on the dorsal and anal fins posteriorly (Fig. 25). The larvae of *Ha. poeyi*, *Ha. garnoti*, and *Ha. bivittatus* are very similar. The molecular phylogeny of labrids by Westneat & Alfaro (2005) did not include *Ha. maculipinna*, but it suggests that *Halichoeres* is paraphyletic without the inclusion of numerous other genera, including *Thalassoma* and *Anampses*. The presence of erythrophores in the three genera on the upper jaw and gut, combined with the presence of distinct dark markings on the dorsal and anal fins, constitute a unique pattern within labriforms that may support a close relationship among these genera.

**Figure 26:**
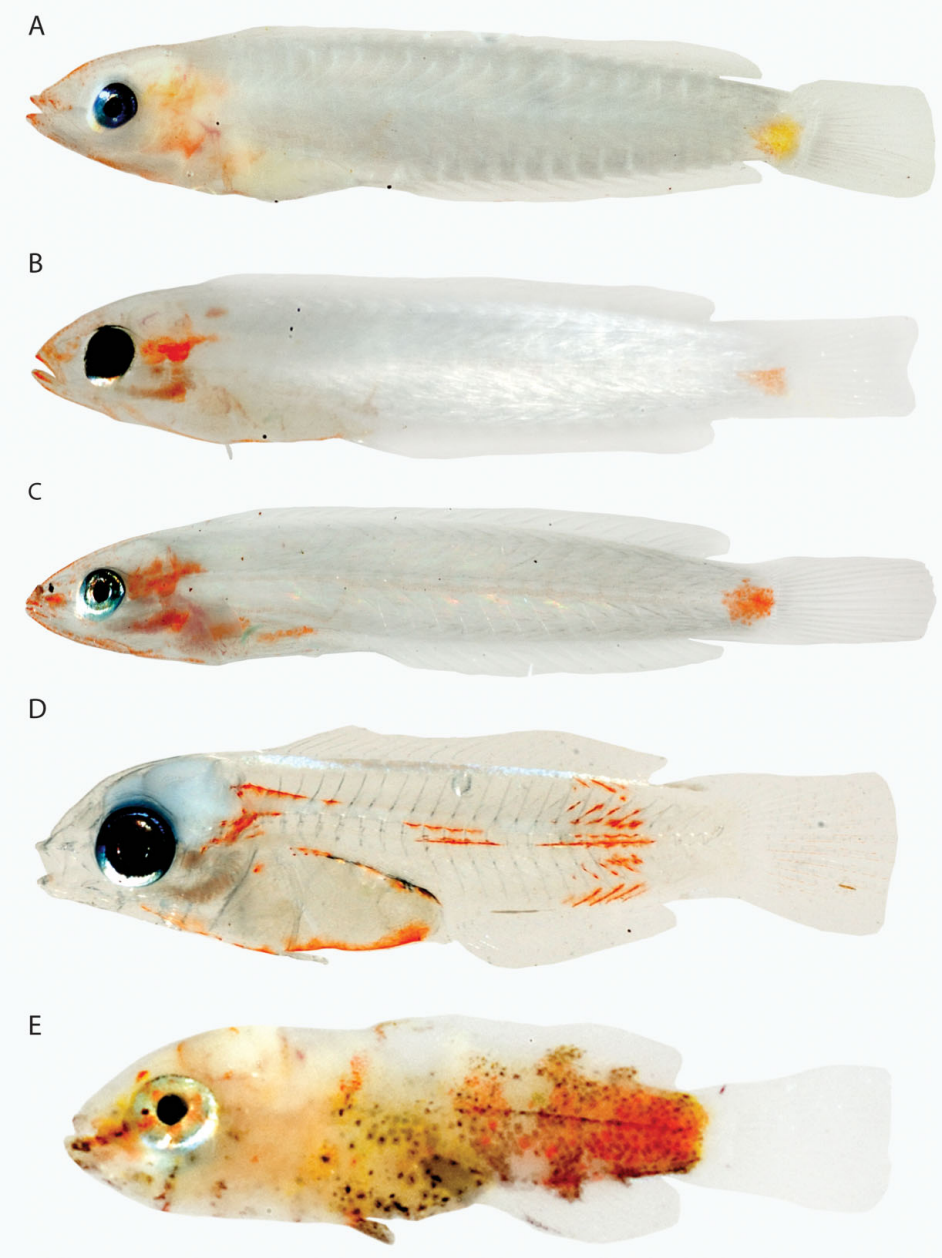
Percomorphacea (Labriformes). A, *Xyrichtys novacula*, 12.0 mm Standard Length (SL), BLZ 5394. B, *Xyrichtys splendens*, 17.0 mm SL, BLZ 7019. C, *Xyrichtys martinicensis*, 15.0 mm SL, Belize. D, *Doratonotus megalepis*, 7.0 mm SL, Belize. E, *Lachnolaimus maximus*, 7.0 mm SL, BLZ 7373. Photos A, C, D by Julie Mounts and Carole Baldwin; B, E by Julie Mounts and David Smith.

*Xyrichtys* larvae are elongate and consistently have erythrophores on both jaws and behind the eye. The rest of the body is pale except for a blotch of colour on the caudal peduncle (Fig. 26A–C). In *Xyrichtys novacula* (Fig. 26A) this blotch is always yellow, whereas in *Xyrichtys splendens* (Fig. 26B) and *Xyrichtys martinicensis* (Fig. 26C) it is orange. Differences in size and shape of the orange blotch distinguish these two species. There is little if any intraspecific variation in chromatophore pattern among the individuals of each *Xyrichtys* species examined.

*Doratonotus* and *Lachnolaimus* are monotypic genera, and their larvae are clearly distinct from one another and other labrids (Fig. 26D, E). *Doratonotus* larvae have internal erythrophores along the vertebral column and along the myosepta of the posterior third of the trunk and, as noted above, lack erythrophores on the jaws. *Lachnolaimus* larvae are very different from those of other labrids in having almost the entire body covered with orange/yellow chromatophores – mostly xanthophores anteriorly and erythrophores posteriorly.

#### Lophiiformes

**Figure 27:**
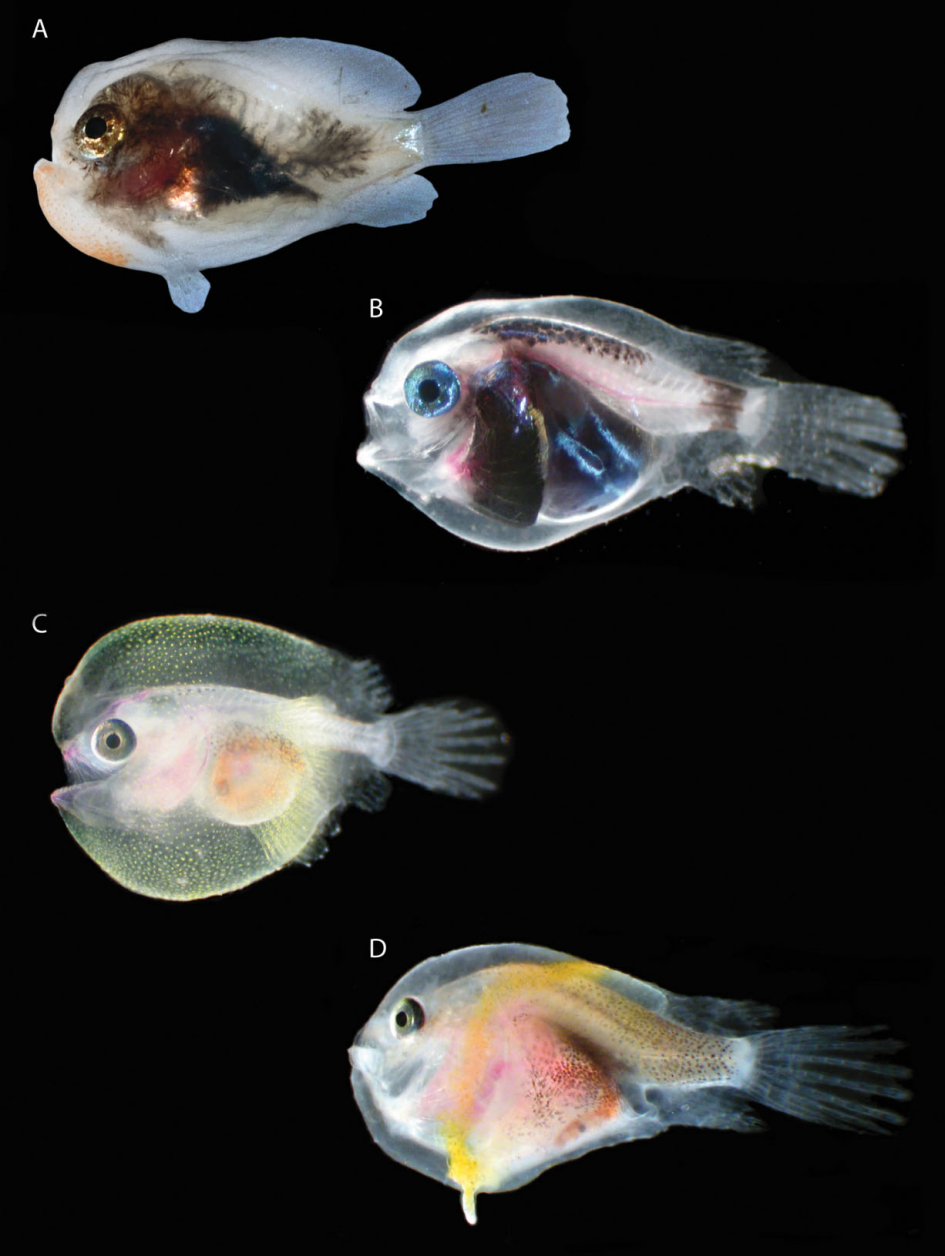
Percomorphacea (Lophiiformes). A, *Antennarius pauciradiatus*, 6.0 mm Standard Length (SL), BLZ 6043. B−D, unknown lophiiforms, Florida Straits. Note: the internal pink coloration in the images appears to be associated with the circulatory system, not chromatophore patterns. Photo A by Lee Weigt and Carole Baldwin; B−D by Cedric Guigand, B and C previously published in Cowen *et al*. (2007) and Fogarty & Botsford (2007), respectively.

Lophiiformes comprise more than a dozen percomorph families, but larvae of only one species from Belize, *Antennarius pauciradiatus* (Antennariidae), have been identified (Fig. 27A). Images of colour patterns in several unidentified lophiiform larvae from the Florida Straits were used for comparative purposes (Fig. 27B–D). In *Ant. pauciradiatus* the distended skin forming the characteristic lophiiform ‘balloon’ around the head and body is lightly covered with erythrophores in the lower jaw and gular regions. The three unidentified lophiiform larvae have different colour patterns from *Ant. pauciradiatus* and one another, two of them exhibiting xanthophores (Fig. 27C, D) and one of them seemingly lacking xanthophores/erythrophores and exhibiting only blue iridophores (Fig. 27B). Lophiiforms, which form part of Rosen & Patterson's (1969) and Patterson & Rosen's (1989) Paracathopterygii, were hypothesized to be percomorph fishes in the molecular phylogeny of Miya *et al*. (2003). The ‘paracanthopts’ examined for colour patterns in larvae are gadids (Fig. 6D), lophiiforms, and ophidiiforms (see below). With the limited amount of material available, the only observation relevant here is that unlike the gadid *Bregmaceros*, which lacks xanthophores, erythrophores, and iridophores, all lophiiforms (and ophidiiforms) examined have one or more of those types of chromatophores. Like numerous other acanthomorph orders, lophiiforms are currently considered *incertae sedis* within the Percomorphacea (Fig. 1), but the hypothesis of Holcroft & Wiley (2008) that lophiiforms are closely related to tetraodontiforms is discussed below (see Tetraodontiformes).

#### Ophidiiformes

**Figure 28:**
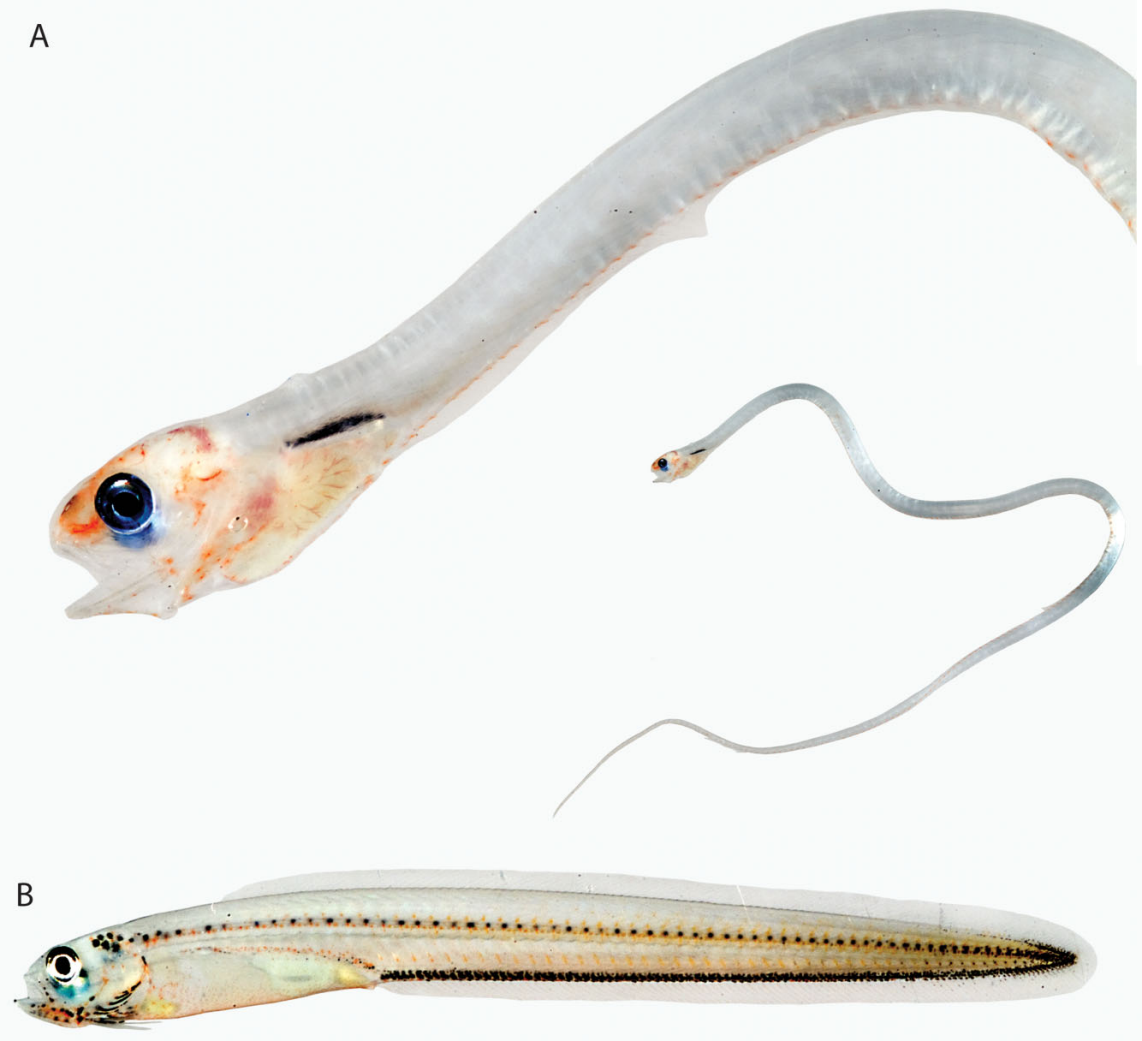
Percomorphacea (Ophidiiformes). A (and inset), *Carapus bermudensis*, 152 mm Standard Length (SL), BLZ 8462. B, *Parophidion schmidti*, 36.5 mm SL, BLZ 8459. Photos by Julie Mounts and David Smith.

**Figure 29:**
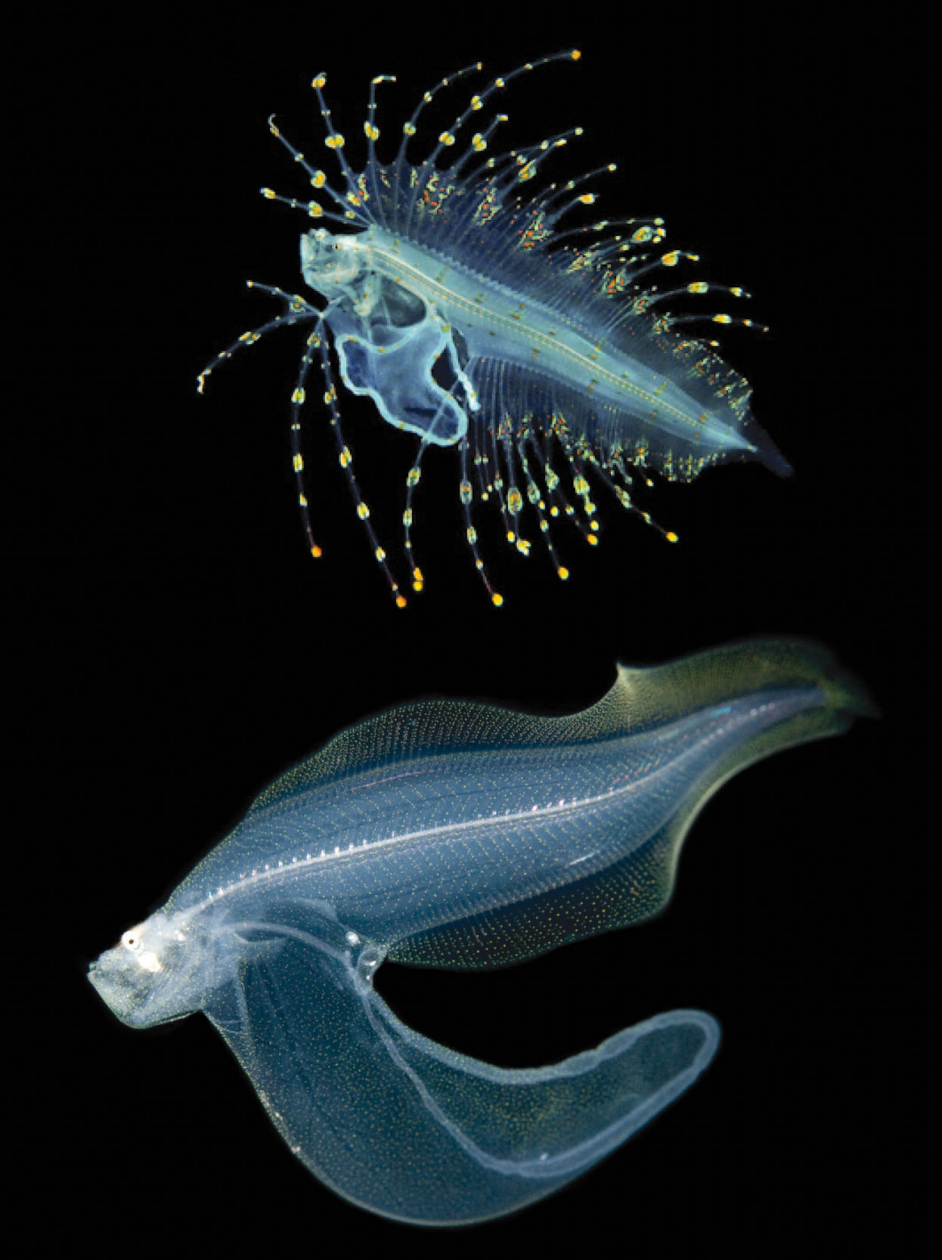
Percomorphacea (Ophidiiformes). Top, *Brotulataenia* sp. and bottom, *Lampogrammus* sp., Hawaii. Photos by Joshua Lambus.

Ophidiiformes were also hypothesized to be percomorphs rather than paracanthopterygians by Miya *et al*. (2003, 2005). Wiley & Johnson (2009) noted that no convincing evidence for the monophyly of the order (ophidioids + bythitoids) exists. Colour information in larvae is known for two ophidioids from Belize, *Carapus bermudensis* (Carapidae) and *Parophidion schmidti* (Ophidiidae), both of which have conspicuous orange/yellow chromatophore patterns (Fig. 28). *In situ* images of two Hawaiian ophidiids that appear to be larval *Brotulataenia* and *Lampogrammus* (Fig. 29) show strikingly beautiful larvae with numerous xanthophores on the body and fins, as well as erythrophores on the fins in the former. Erythrophores in *Parophidion* are confined to the head and trunk and do not extend onto the median fins. No conclusions about the potential phylogenetic significance of colour patterns in larval ophidioids can be drawn at this time, and colour patterns, if any, in young bythitoids are unknown.

#### Perciformes

**Figure 30:**
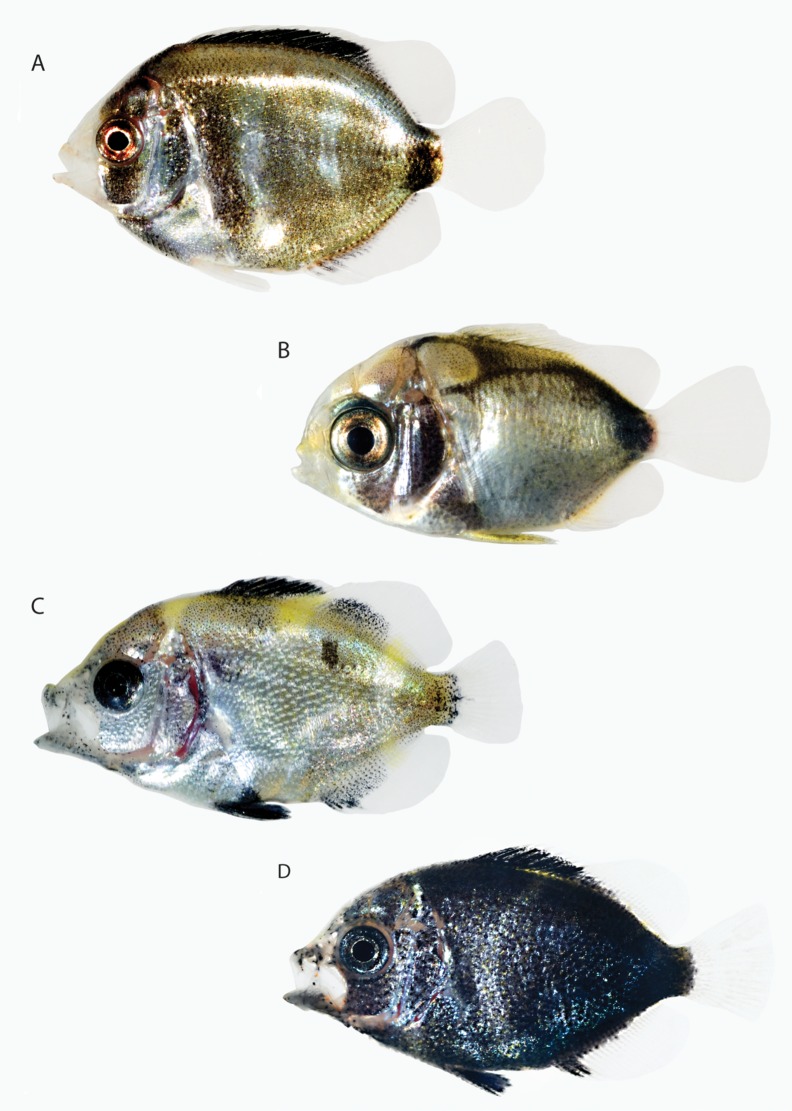
Percomorphacea (Perciformes). A, *Holacanthus ciliarus*, 16.0 mm Standard Length (SL), BLZ 8477. B, *Chaetodon capistratus*, 12.0 mm SL, BLZ 8436. C, *Pomacanthus arcuatus*, 10.0 mm SL, BLZ 10101. D, *Pomacanthus paru*, 10.0 mm SL, BLZ10213. Photos A, B by Julie Mounts and David Smith; C, D by Donald Griswold and Carole Baldwin.

**Figure 31:**
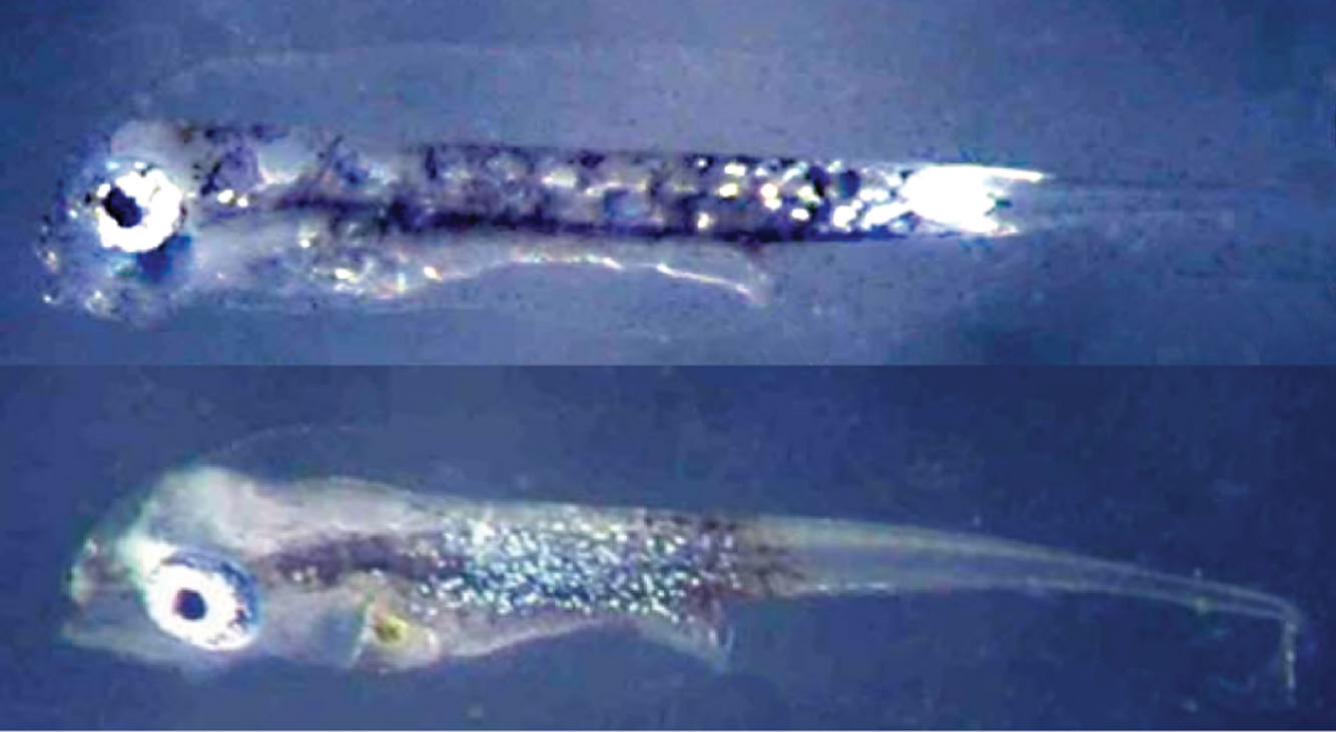
Percomorphacea (Perciformes). Top, preflexion larva of *Chaetodon marleyi*, 3.8 mm Notochord Length (NL). Bottom, preflexion larva of *Pomacanthus rhomboides*, 3.9 mm NL. Both images are of specimens from off South Africa. Photos by Allan Connell.

Perciformes remain a conglomerate of families and *incertae sedis* genera that are not united by synapomorphies (Wiley & Johnson, 2009), and larvae of the group exhibit a diverse array of chromatophore patterns. At the interfamilial level, the Chaetodontidae and Pomacanthidae have larvae that are covered with iridophores – silver/bronze in *Holacanthus*, *Chaetodon*, and *Pomacanthus arcuatus*, silver/blue in *Pomacanthus paru* (Fig. 30). Although not as conspicuous in *Holacanthus* (Fig. 30A) and *Pomac. paru* (Fig. 30D) as in *Chaetodon* and *Pomac. arcuatus*, larvae of all specimens of all three genera have xanthophores, erythrophores, or both, minimally on the jaws, snout, base of dorsal fin, and caudal peduncle. It does not appear that chaetodontids and pomacanthids resemble smegmamorphs in transitioning from an early stage featuring predominantly xanthophores/erythrophores to a later stage featuring primarily iridophores, as preflexion *Chaetodon marleyi* and *Pomacanthus rhomboides* have few if any xanthophores or erythrophores (Fig. 31). The preflexion *Chaetodon* and *Pomacanthus* are quite similar in having a broad band of iridescent silvery blue iridophores around the trunk. Chaetodontids and pomacanthids have been considered to be so closely related in the past that until Burgess' (1974) publication, pomacanthids were classified as a subfamily of the Chaetodontidae. The families were still considered closely related by Tyler *et al*. (1989), who delineated a monophyletic group comprising chaetodontids, pomacanthids, and *Drepane* (Drepaneidae) based on configuration of the ethmoid bone. Colour in larval *Drepane* is unknown, but the presence in larvae of that genus of a colour pattern comprised largely of iridophores with xanthophores/erythrophores positioned as described above for chaetodontids and pomacanthids could provide corroborative evidence for the monophyly of the group. Recent molecular studies have challenged the hypothesis of a close relationship between chaetodontids and pomacanthids. Using mitochondrial genes, Bellwood, van Herwerden & Konow (2004) hypothesized that chaetodontids are more closely related to scatophagids than to pomacentrids, and Holcroft & Wiley (2008) hypothesized that chaetodontids and scatophagids are part of a large group also comprising acanthuroids, lophiiforms, and tetraodontiforms but not pomacanthids. Colour information for larval scatophagids is not available, but colour in larvae would not appear to support a closer relationship between chaetodontids and acanthurids (Figs 14, 15) than between chaetodontids and pomacanthids (Fig. 30). Larval chaetodontids bear little resemblance to larval lophiiforms (Fig. 27), but larval tetraodontid tetraodontiforms examined are similar to chaetodontids and pomacanthids in having the body covered with bronze/gold iridophores and the abdominal region silvery (see ‘Tetraodontiformes’ below).

**Figure 32:**
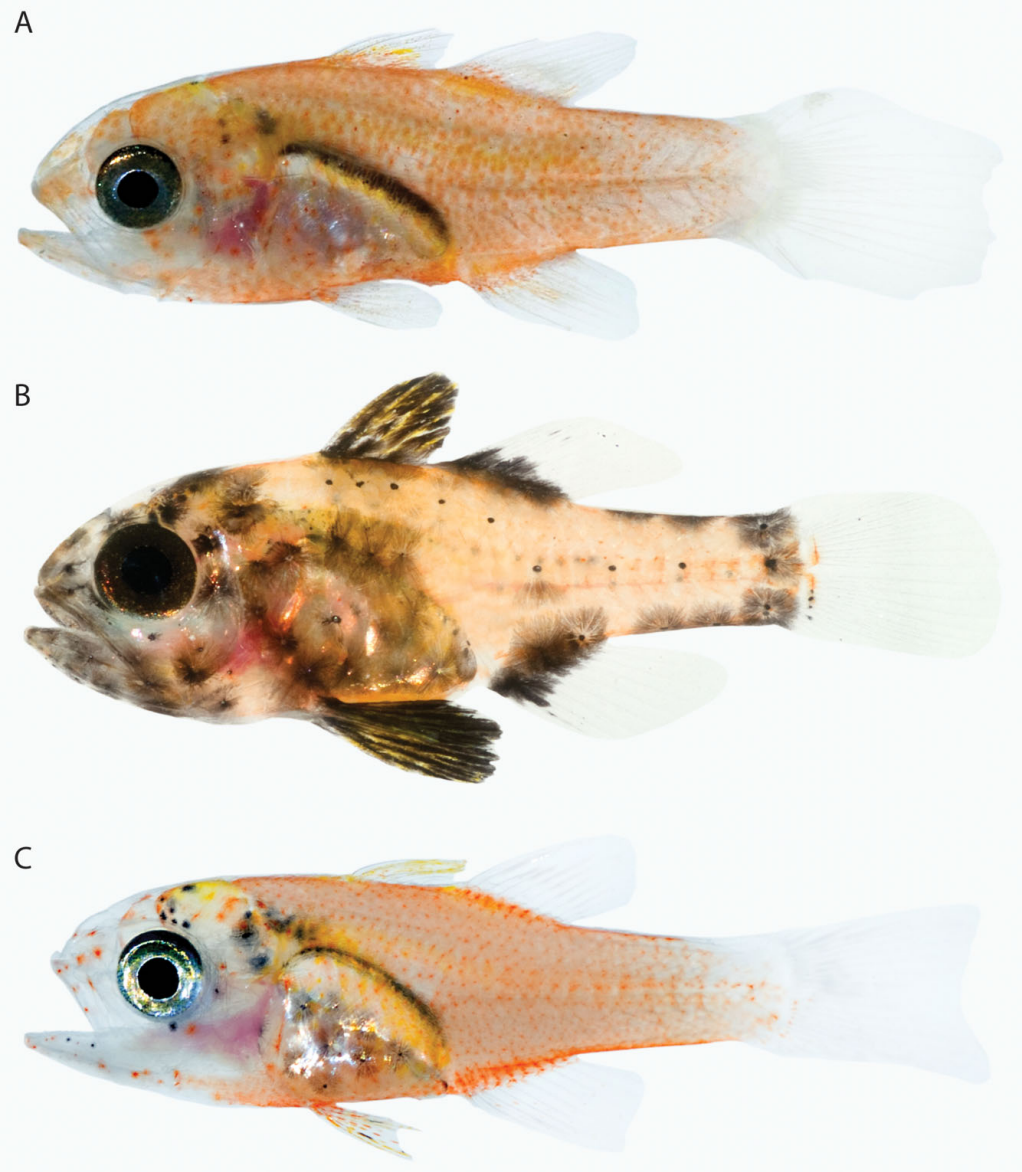
Percomorphacea (Perciformes). A, *Apogon aurolineatus*, 9.0 mm Standard Length (SL), BLZ 10001. B, *Astrapogon puncticulatus*, 13.0 mm SL, BLZ 7125. C, *Phaeoptyx xenus*, 10.0 mm SL, BLZ 10220. Photos A, C by Donald Griswold and Carole Baldwin; B by Julie Mounts and David Smith.

At the familial level, larval Apogonidae have nearly the entire body covered with erythrophores. With the exception of *Priolepis* gobies and *Callionymus* dragonettes, no other larvae examined are covered so completely with erythrophores, and this feature may be synapomorphic for the family or some subset of it. Colour patterns in western Atlantic larval *Apogon*, *Astrapogon*, and *Phaeoptyx* (Fig. 32) have been described (Baldwin *et al*., 2009a; 2011). Patterns of melanophores, in conjunction with orange/yellow chromatophores, diagnose the three genera. Within *Apogon*, colour patterns delineate several species groups that may be meaningful in the generic classification of the group (Baldwin *et al*., 2011).

**Figure 33:**
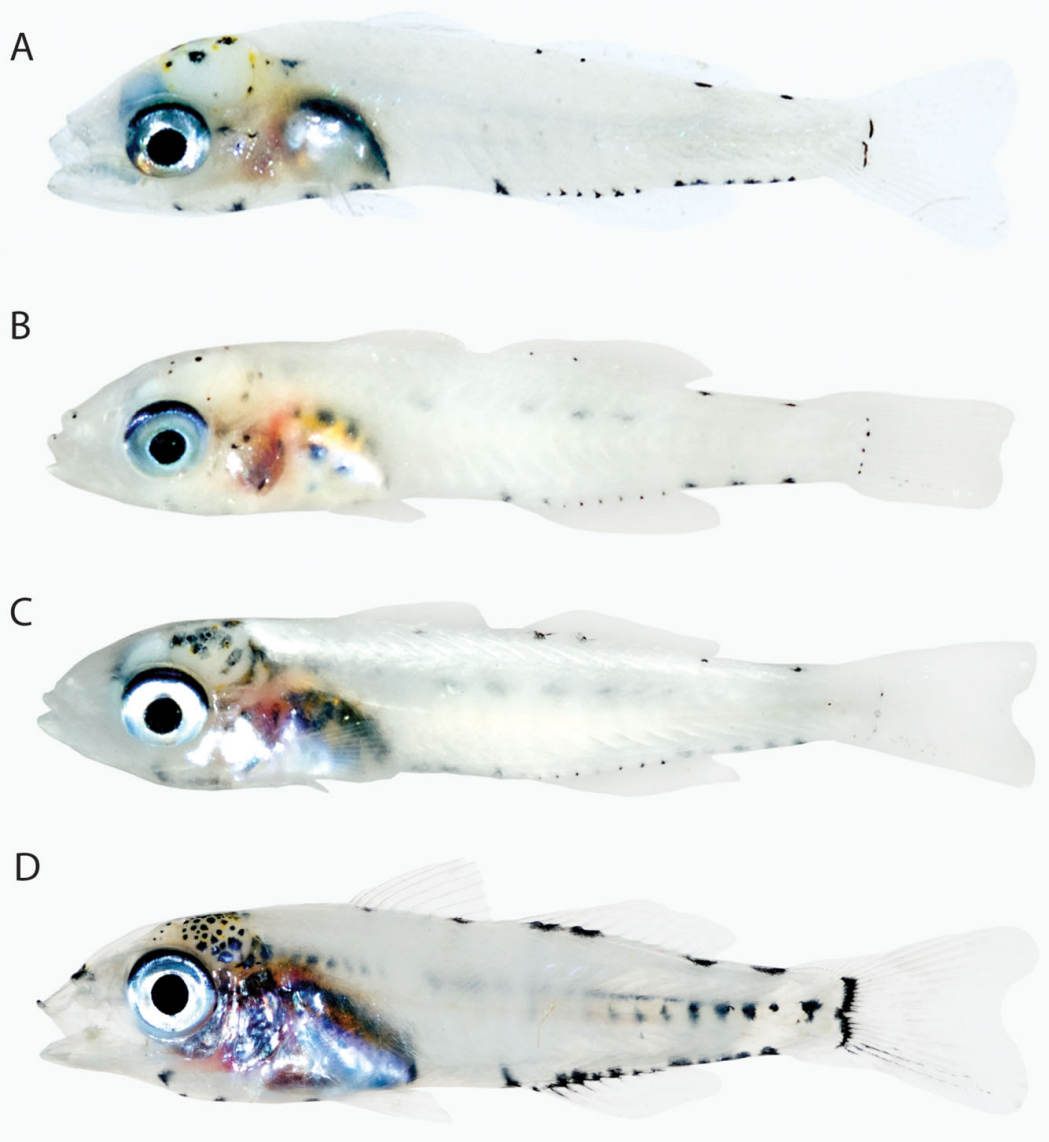
Percomorphacea (Perciformes). A, *Eucinostomus gula*, 9 mm Standard Length (SL), BLZ 10107. B, *Eucinostomus jonesi*, 9.2 mm SL, BLZ 7257. C, *Eucinostomus harengulus*, 12 mm SL, BLZ 7345. D, *Eucinostomus melanopterus*, 15 mm SL, BLZ 10228. Photos A, D by Donald Griswold and Carole Baldwin; B, C by Julie Mounts and David Smith.

Larval Gerreidae examined lack erythrophores and xanthophores on most of the body, but some pale yellow pigment is usually present over the gut and swimbladder and sometimes on the dorsal portion of the head (Fig. 33). Determining whether this pattern, combined with the distinctive arrangement of melanophores at the bases of the median fins, characterizes *Eucinostomus* (larvae of four species identified) or the entire family requires additional material.

**Figure 34:**
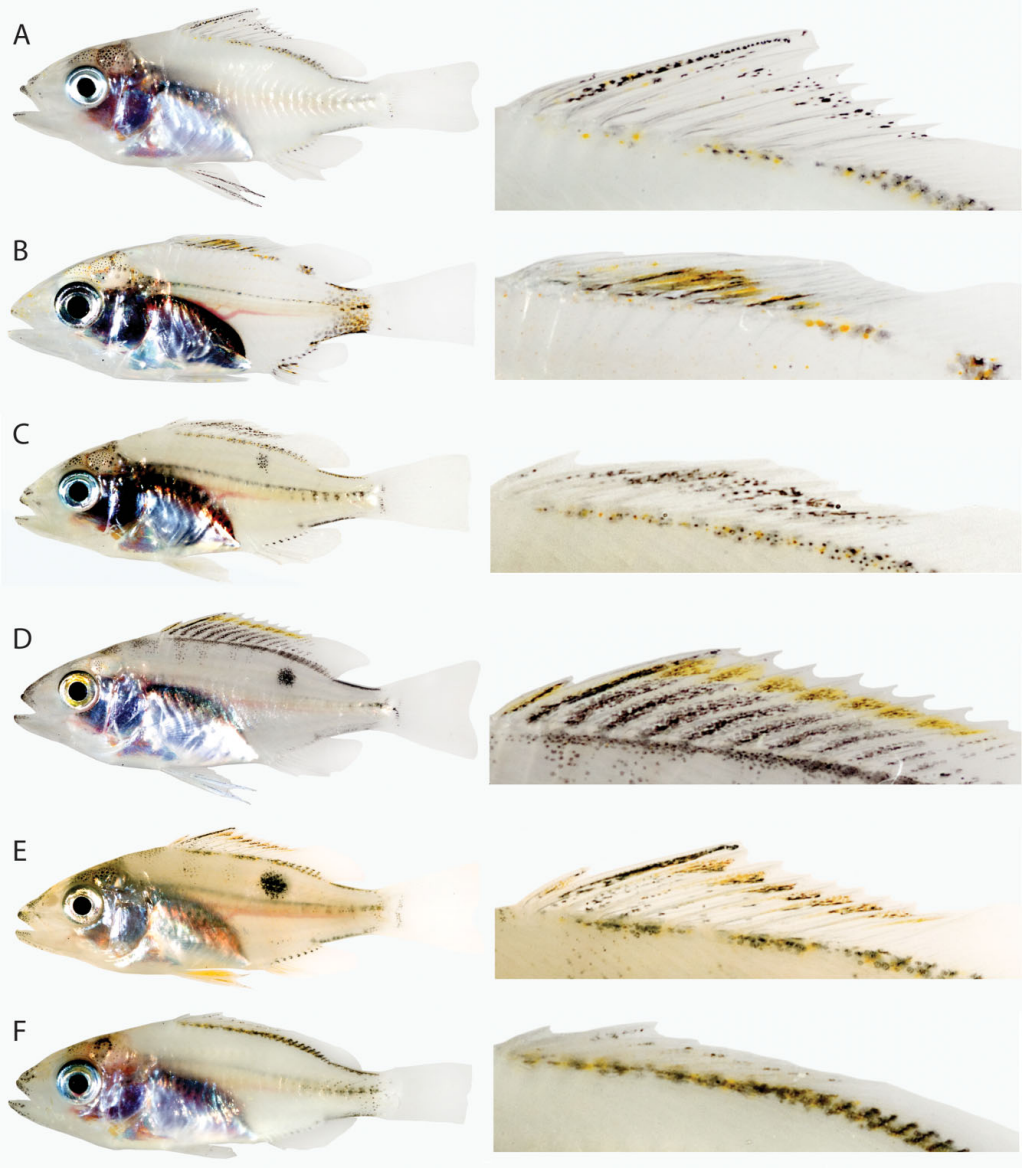
Percomorphacea (Perciformes). A, *Lutjanus analis*, 16.5 mm Standard Length (SL), BLZ 8466. B, *Lutjanus griseus*, 13.5 mm SL, BLZ 8427. C, *Lutjanus synagris*, 18.0 mm SL, BLZ 7150. D, *Lutjanus vivanus*, 26 mm SL, BLZ 8399. E, *Lutjanus mahogani*, 23.0 mm SL, BLZ 5453. F, *Ocyurus chrysurus*, 17.5 mm SL, BLZ 7052. Images in column on right are enlarged views of the dorsal fin of each image in left column. Photos A−C, F by Julie Mounts and David Smith; D by Lee Weigt and Carole Baldwin; E by Lee Weigt and David Smith.

Late-stage larvae of the Lutjanidae are easily recognized by the cap of silver iridophores over the gut and opercular region, a mostly clear trunk, and xanthophores associated with the dorsal fin (or its base) and sometimes caudal peduncle (Fig. 34). The pattern of xanthophores appears species specific within *Lutjanus*. Presence of the same general pattern of iridophores and xanthophores in *Ocyurus chrysurus* (Fig. 34F) may lend support to the hypothesis based on morphological (including larval) and molecular data that *Ocyurus* is a synonym of *Lutjanus* (Domeier & Clarke, 1992; Chow, Clarke & Walsh, 1993; Clark, Domeier & Laroche, 1997), but information on colour patterns in larvae of other lutjanid genera is needed to determine at what taxonomic level the colour pattern in *Lutjanus* is significant. In general appearance, including the presence of melanophores dorsally on the head, a silvery gut, pigment associated with the pelvic-fin spine, and usually orange or yellow chromatophores on the caudal peduncle, larval *Lutjanus* is very similar to larvae of the grouper genus *Mycteroperca* (see ‘Scorpaeniformes’ below).

**Figure 35:**
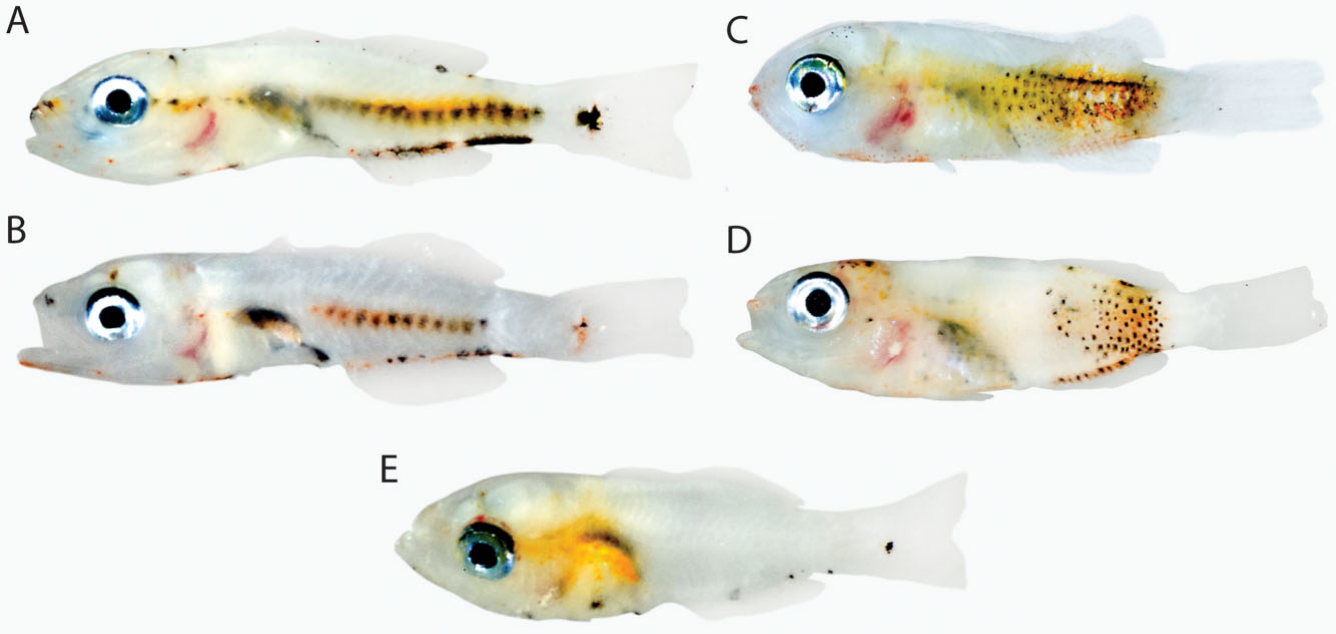
Percomorphacea (Perciformes). A, *Haemulon sciurus*, 8.0 mm Standard Length (SL), BLZ 7369. B, *Haemulon plumieri*, 7.0 mm SL, BLZ 6204. C, *Calamus* sp., 5.0 mm SL, BLZ 6022. D, *Calamus* sp., 9.0 mm SL, BLZ 7256. E, *Anisotremus virginicus*, 5.5 mm SL, BLZ 7346. Photos A, D, E by Julie Mounts and David Smith; B, C by Lee Weigt and Carole Baldwin.

At the generic level, larval *Haemulon* (Haemulidae) has a stripe of xanthophores/erythrophores mixed with dark melanophores on the posterior half of the trunk (Fig. 35A, B). Among perciforms examined, larval *Calamus* is most similar in having a swathe of xanthophores/erythrophores on the posterior portion of the trunk mixed with melanophores (Fig. 35C, D). There are no chromatophores on the posterior portion of the trunk in the haemulid *Anisotremus* (Fig. 35E), but several species of the haemulid genus *Pomadasys* are very similar to *Haemulon* (e.g. *Pomadasys commersonnii*, Connell, 2007; Appendix). There is no consensus based on morphological or molecular data for inter-relationships of haemulids and sparids among perciforms.

**Figure 36:**
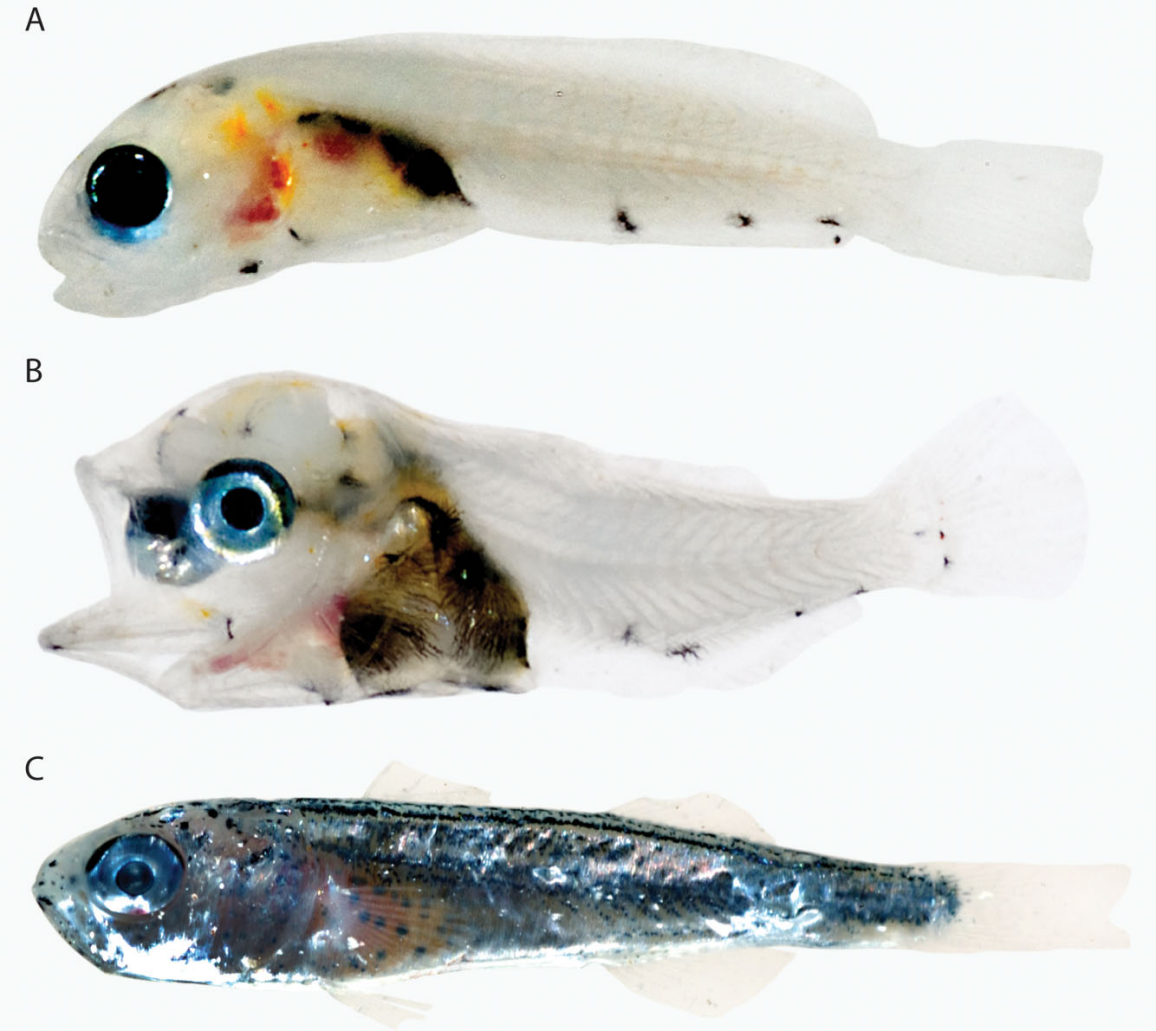
Percomorphacea (Perciformes). A, Opistognathidae?, 9.0 mm Standard Length (SL), BLZ 6394. B, *Odontoscion dentex*, 6.0 mm SL, BLZ 10153. C, *Upeneus parvus*, 10.5 mm SL, BLZ 4505. Note: internal red coloration in the thoracic region of A is associated with the circulatory system, not chromatophores. Photo A by Julie Mounts and David Smith; B by Donald Griswold and Carole Baldwin; C by Lee Weigt and David Smith.

A larva tentatively identified as an opistognathid (Fig. 36A) and the sciaenid *Odontoscion* (Fig. 36B) lack orange/yellow chromatophores on the trunk and have xanthophores confined to the head and gut region. A larval Mullidae, *Upeneus parvus*, does not resemble any other perciform larvae examined in that it is covered with melanophores and blue iridophores (Fig. 36C). Based on mitochondrial and nuclear DNA data, Smith & Craig (2007) suggested a close relationship between mullids and a nonperciform family, Dactylopteridae (Dactylopteriformes are *incertae sedis* in percomorphs in the classification of Wiley & Johnson, 2009). Although colour information is lacking, dactylopterid larvae have huge head spines that are lacking in *Upeneus*. Possibly the dense covering of iridophores in *Upeneus* larvae will be of value in identifying its closest relatives in the future. Preflexion larvae of *Oplegnathus* (Oplegnathidae), *Pempheris* (Pempheridae), *Neoscorpis* (Scorpididae), and several other perciform families from off South Africa have a considerable amount of yellow pigment on the trunk (Connell, 2007; Appendix), but larger specimens are needed for comparisons with other postflexion perciform larvae.

#### Pleuronectiformes

**Figure 37:**
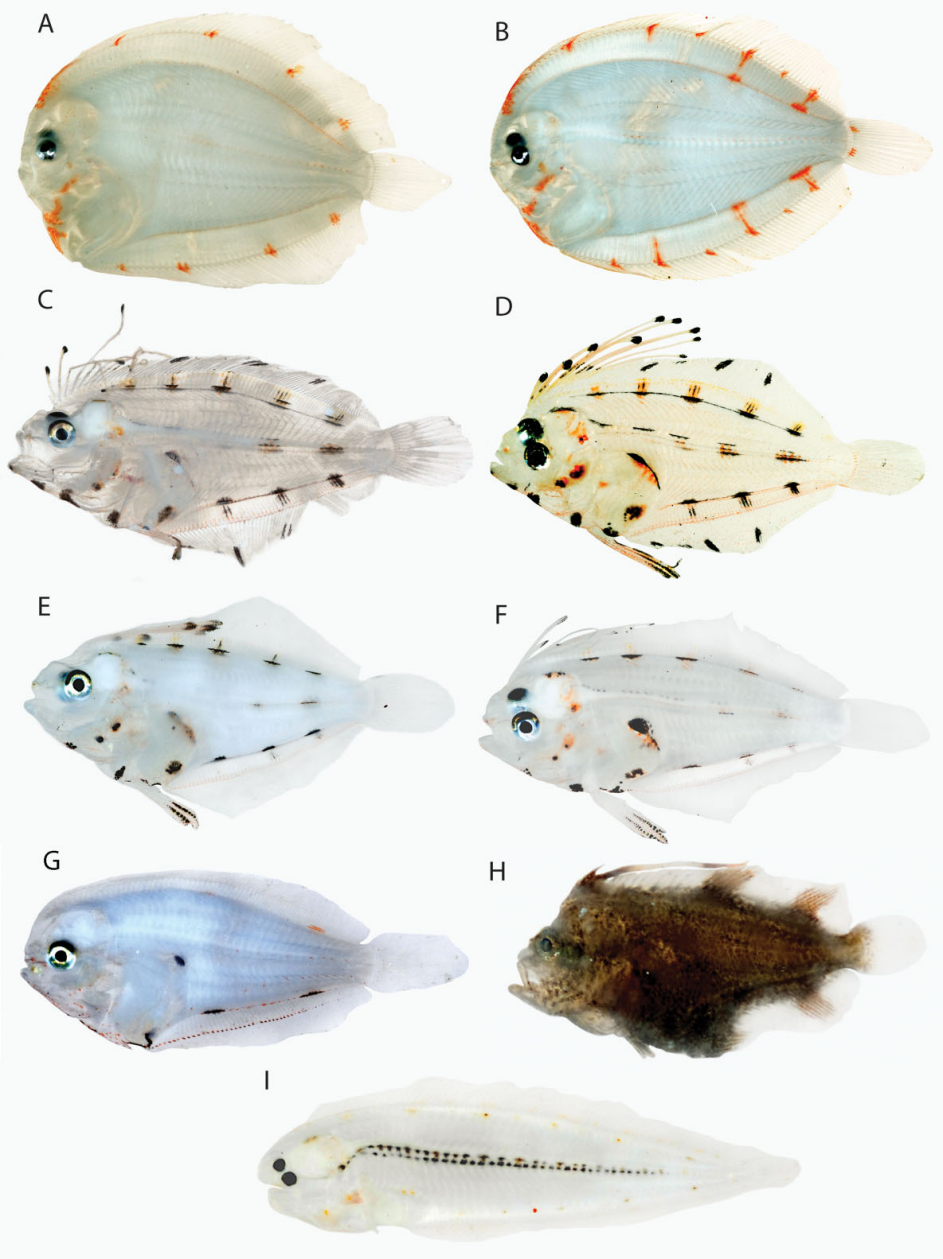
Percomorphacea (Pleuronectiformes). A, *Bothus maculiferus*, 13.0 mm Standard Length (SL), BLZ 4219. B, *Bothus ocellatus*, 19.0 mm SL, Belize. C, *Syacium* sp., 17.0 mm SL, Belize. D, *Syacium* sp., 15.5 mm SL, BLZ 7078. E, *Syacium* sp., 12.0 mm SL, BLZ 6010. F, *Syacium* sp., 13.0 mm SL, BLZ 8463. G, *Citharichthys* sp., 9.5 mm SL, BLZ 6006. H, *Trinectes* sp., 5.0 mm SL, BLZ 10161. I, *Symphurus* sp., 11 mm SL, BLZ 7779. Photo A by Lee Weigt and David Smith; B, C by Julie Mounts and Carole Baldwin; D, F by Julie Mounts and David Smith; E, G, I by Lee Weigt and Carole Baldwin; H by Donald Griswold and Carole Baldwin.

**Figure 38:**
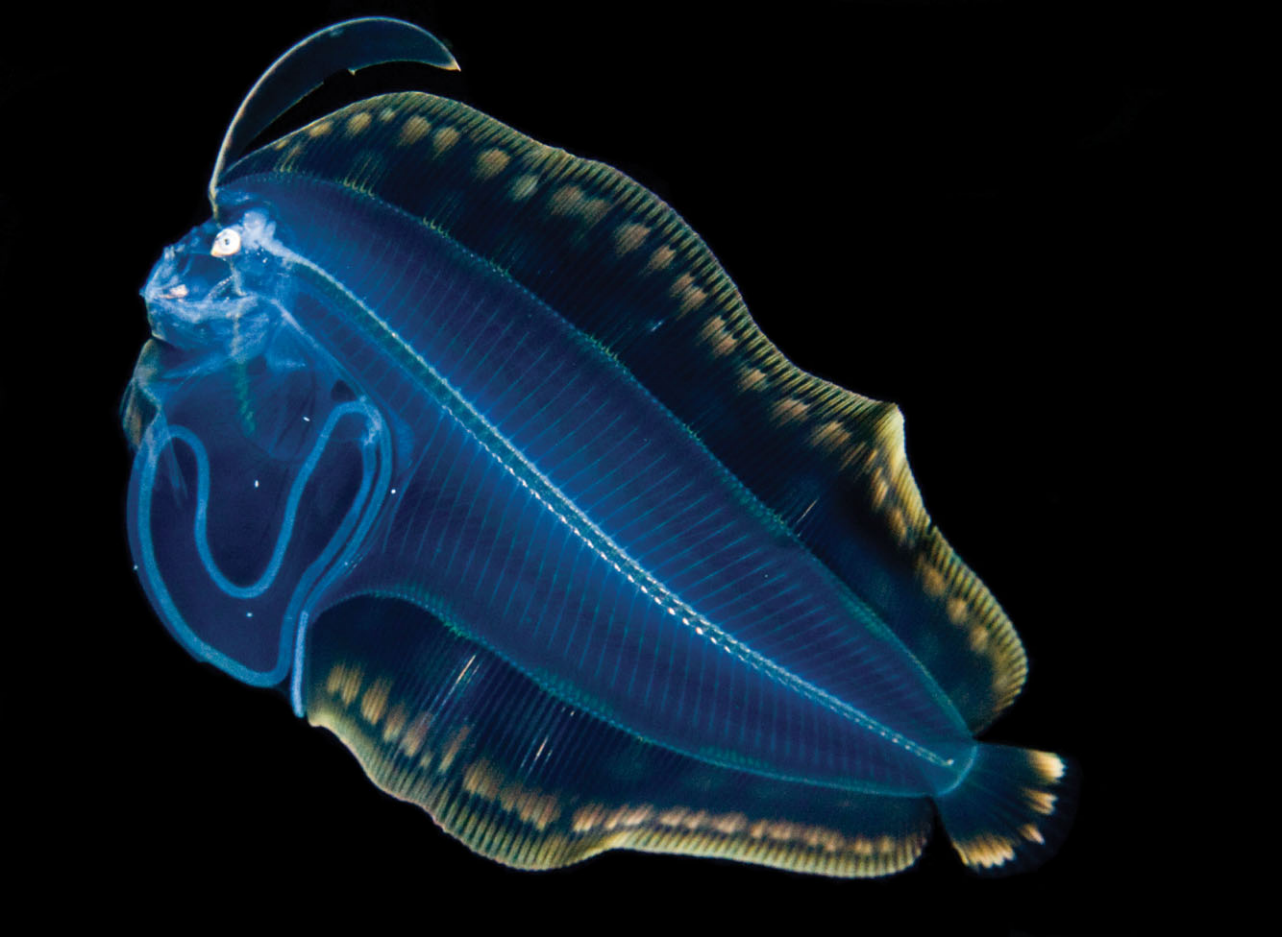
Percomorphacea (Pleuronectiformes). Unknown pleuronectiform, Hawaii. Photo by Joshua Lambus.

Pleuronectiformes are united as a monophyletic group in part based on ontogeny (transformation from a bilaterally symmetrical larva to an asymmetrical adult), and one or more elongate anterior dorsal-fin elements characterize larvae of several families (Hensley & Ahlstrom, 1984). Colour patterns in larvae should prove useful phylogenetically at some levels. The presence of bright orange erythrophores on the median fins and fin bases, pelvic fin, head, and lateral midline (where they are small to minute) in the bothids *Bothus maculiferus* and *Bothus ocellatus*, combined with the absence of melanophores, may be synapomorphic for the genus (Fig. 37A, B). Superficially different from *Bothus* larvae because of the presence of numerous melanophores, larvae of the paralichthyid genus *Syacium* (e.g. Fig. 37C−F) are quite similar to *Bothus* in nonmelanistic colour pattern. Larvae of *Syacium papillosum* and three unidentified species have orange/yellow pigment on the median fins and fin bases, pelvic fin, head, and lateral midline, but also in a distinct patch on the dorsal portion of the gut. A single, small *Citharichthys* larva (Paralichthyidae) appears to have a similar pattern, but erythrophores are tiny (or contracted), and it is difficult to discern their precise distribution (Fig. 37G). Bothids and at least some paralichthyids (including *Citharichthys* and *Syacium*) consistently appear as sister groups or components of a slightly larger monophyletic group based on morphology and molecules (Cooper & Chapleau, 1998; Berendzen & Dimmick, 2002; Pardo *et al*., 2005; Azevedo *et al*., 2008), and the general colour pattern described above may help define a clade that includes bothids and those paralichthyids. However, the more distantly related Cynoglossidae (one *Symphurus* examined) has a similar colour pattern, with orange pigment present on the median fins and fin bases, head, and lateral midline (Fig. 37I). The single Achiridae examined (*Trinectes*) is heavily covered with melanophores, largely obscuring the nonmelanistic colour pattern, but pale orange pigment is visible on the median and pelvic fins (Fig. 37H). An *in situ* image of an unidentified pleuronectiform larva from off Hawaii, possibly a bothid, has the same general colour pattern observed in most pleuronectiforms – i.e. yellow/orange pigment on the median fins (dorsal and anal fins almost completely covered with pigment), dorsal- and anal-fin bases, pelvic fin, head, and lateral midline (Fig. 38).

Species differences in the material examined are evident in the configuration of erythrophores, *Bothus* serving as an excellent example (Fig. 37A, B). In the absence of melanophores, preserved larvae of *Bo. ocellatus* and *Bo. maculiferus* lack diagnostic pigment patterns, but fresh specimens are easily distinguished by the pattern of erythrophores (*Bo. ocellatus* with more orange markings along the dorsal- and anal-fin bases than *Bo. maculiferus* and with erythrophores on the base of the caudal fin, in dashes along the dorsal and ventral body margins posteriorly, and in lines between those dashes and the orange markings along the bases of the dorsal and anal fins vs. none of these markings in *Bo. maculiferus*). As noted under ‘Carangiformes’, a relationship between pleuronectiforms and carangiforms as proposed by Little *et al*. (2010) would not appear to be supported by colour patterns in larvae, nor would a proposed relationship (Smith & Craig, 2007) with *Xiphias gladius* (see ‘Scombriformes’ below).

#### Scombriformes

**Figure 39:**
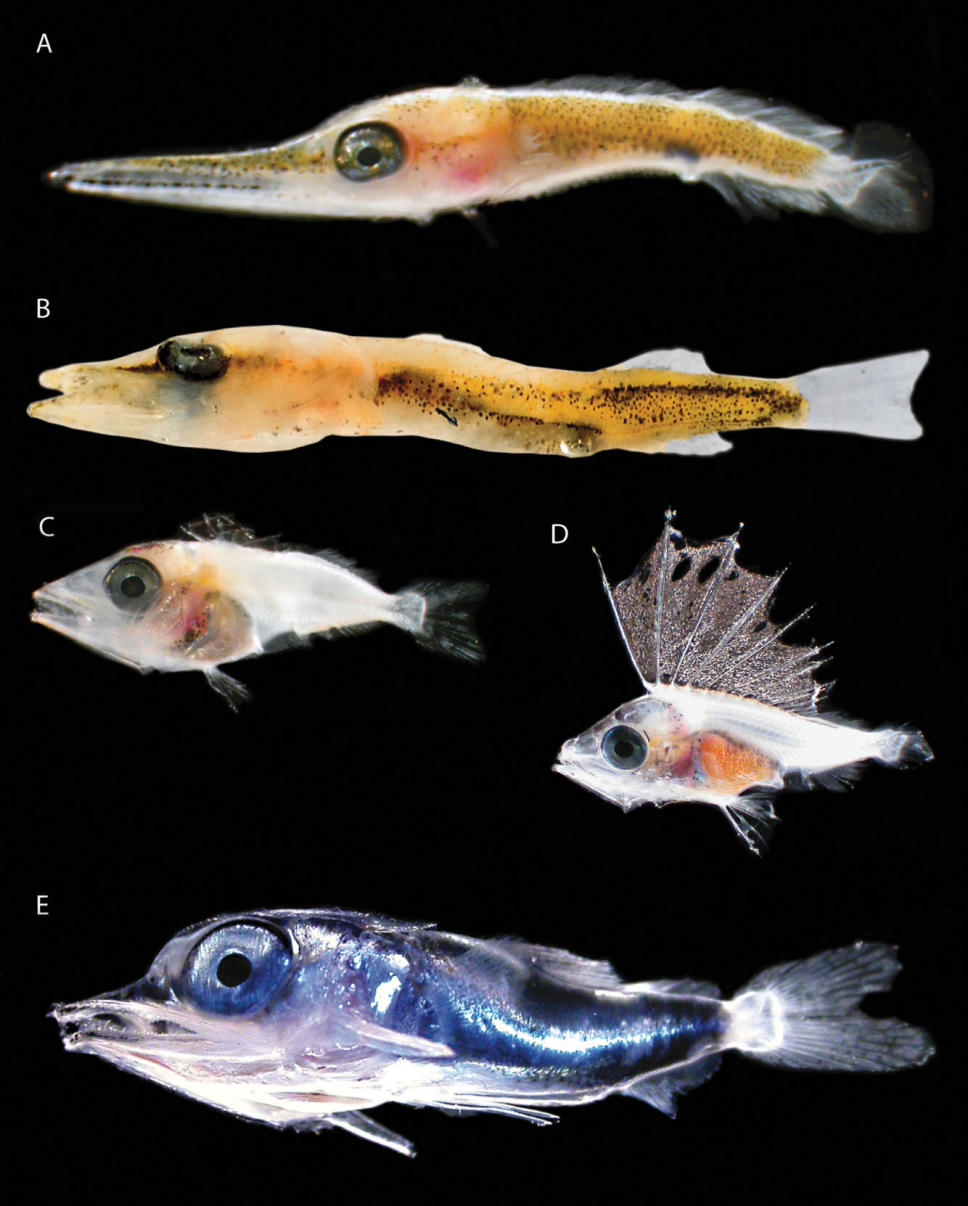
Percomorphacea (Scombriformes). A, *Xiphias gladius*. B, *Sphyraena barracuda*, 8.0 mm Standard Length, Belize. C, Scombridae. D, Gempylidae. E, Istiophoridae. Photos A, C−E by Cedric Guigand (specimens from Florida Straits), B by Julie Mounts and Carole Baldwin.

Scombriformes are represented in the material examined by larvae of the Gempylidae, Istiophoridae, Scombridae, Sphyraenidae, and Xiphiidae. Early postflexion larvae of *Xiphias* (Fig. 39A) and *Sphyraena* (Fig. 39B) have extremely similar colour patterns – yellow extending from the snout to the caudal peduncle with numerous melanophores mixed in. A larval scombrid examined, by contrast, is mostly pale, with only a small amount of yellow pigment over the gut and pale orange pigment along the anal-fin base and lateral midline (Fig. 39C, see also http://vertebrates.si.edu/fishes/larval/perci.html for an image of a preflexion *Thunnus* that is similar). A postflexion *Auxis* from off South Africa has some reddish pigment over the gut but is otherwise similarly pale (Connell, 2007; Appendix). A larval gempylid is similar to the scombrids examined in having a mostly pale head and trunk, but the spinous dorsal fin is densely pigmented with melanophores and bronze iridophores (Fig. 39D). A larval istiophorid examined does not resemble any of the other scombriform larvae in that nearly the entire body is covered with blue-reflecting iridophores (Fig. 39E). The relationship of billfishes to scombrids has been the subject of much controversy (e.g. Collette *et al*., 1984; Johnson, 1986; Orrell, Collette & Johnson, 2006; Little *et al*., 2010), and larval colour patterns do not appear to shed much light on the matter. The most recent molecular hypotheses (Orrell *et al*., 2006; Little *et al*., 2010) suggest that billfishes are not the closest relatives of scombrids. Orrell *et al*. (2006: fig. 3) proposed a sister-group relationship between *Sphyraena* and *Xiphias* + Istiophoridae, and, if apomorphic, the similar colour patterns in larval *Sphyraena* and *Xiphias* could provide further support for this hypothesis.

#### Scorpaeniformes

**Figure 40:**
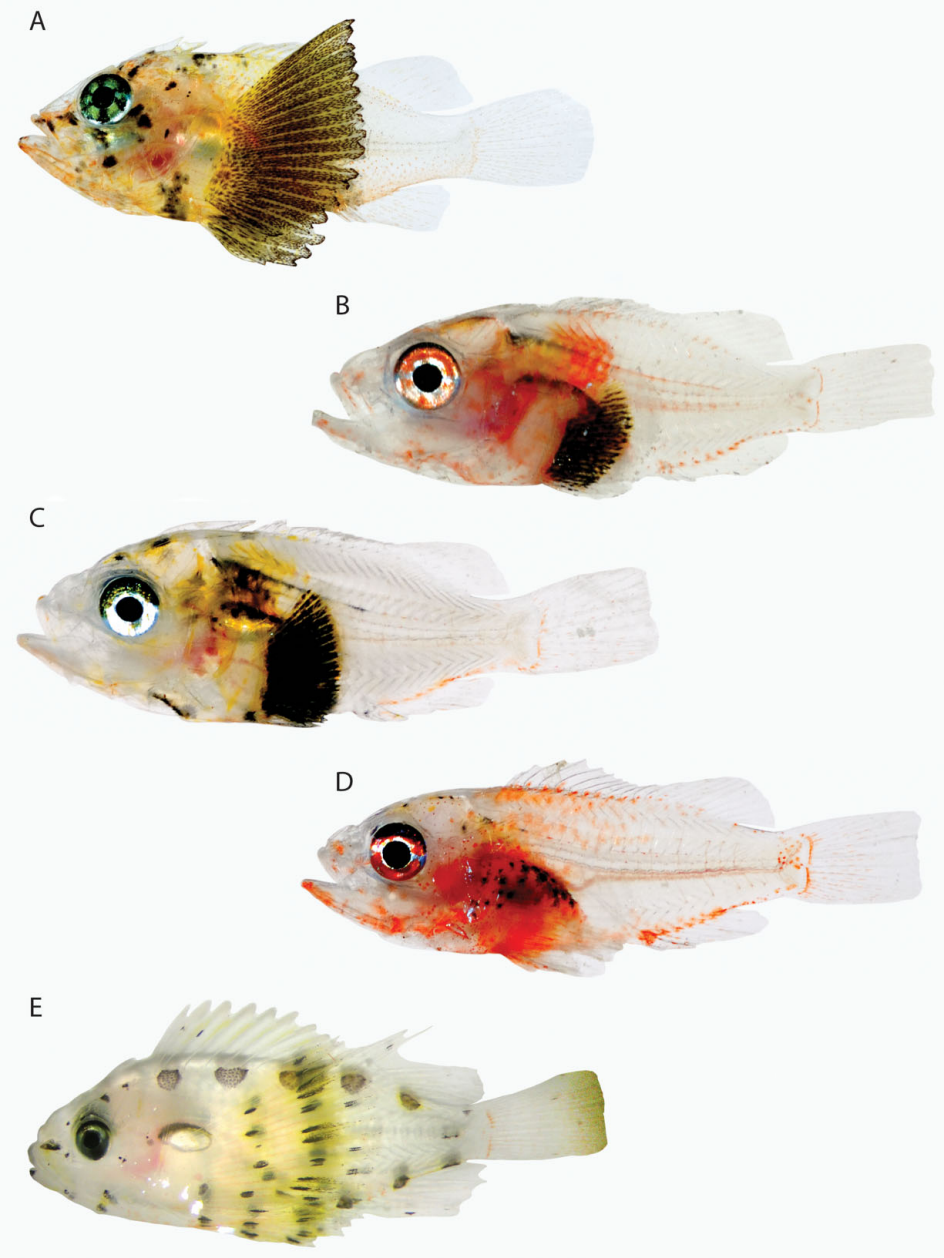
Percomorphacea (Scorpaeniformes). A, *Scorpaenodes carribaeus*, 9.0 mm Standard Length (SL), BLZ 6019. B, *Scorpaena inermis*, 7.0 mm SL, Belize. C, *Scorpaena grandicornis*, 7.5 mm SL, BLZ 10215. D, *Scorpaena bergi*, 10 mm SL, BLZ 10232. E, *Dendrochirus brachypterus*, South Africa. Photo A by Lee Weigt and Carole Baldwin; B by Julie Mounts and Carole Baldwin; C, D by Donald Griswold and Carole Baldwin; E by Allan Connell.

**Figure 41:**
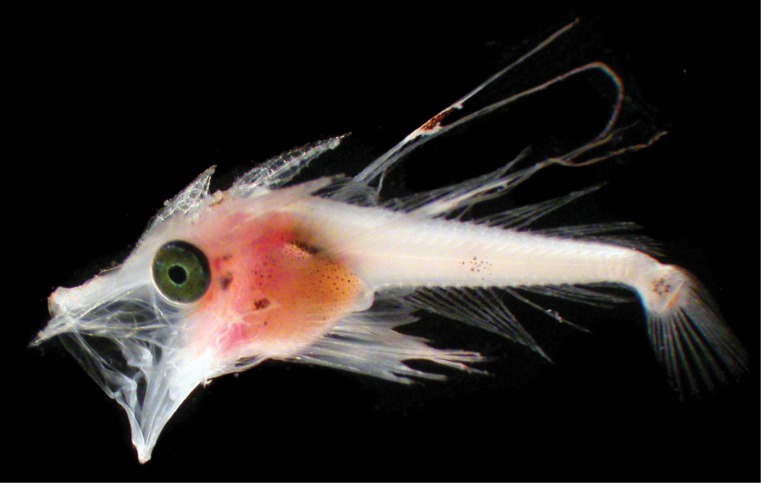
Percomorphacea (Scorpaeniformes). *Peristedion* sp., Florida Straits. Photo by Cedric Guigand.

Scorpaeniformes traditionally have comprised the scorpaenids and their mail-cheeked relatives (e.g. Nelson, 2006), but Wiley & Johnson (2009) followed Imamura & Yabe (2002) in placing scorpaenoids, platycephaloids, and the Serranidae in the Scorpaeniformes. Some molecular data conflict with this hypothesis and the proposed monophyly of the component suborders – Scorpaenoidei and Serranoidei (Smith & Wheeler, 2004; Smith & Craig, 2007). All scorpaeniforms for which colour in larvae has been examined – the scorpaenids *Scorpaena*, *Scorpaenodes*, *Dendrochirus*; the peristiid, *Peristedion*; and the serranids *Diplectrum*, *Serranus*, *Hypoplectrus*, *Gonioplectrus*, *Mycteroperca*, *Bathyanthias*, *Liopropoma*, *Pseudogramma*, *Rypticus*, and *Pseudanthias –* have orange/yellow chromatophores on the body. The three scorpaenid genera have erythrophores or xanthophores on an enlarged pectoral fin, the placement and extent of the colour aiding generic recognition: in *Scorpaenodes* nearly the entire fin is covered with xanthophores and melanophores with sometimes the very proximal area clear (Fig. 40A); in *Scorpaena* yellow or orange chromatophores – and sometimes dense melanophores – cover the proximal portion of the fin, and the distal portion is clear (Fig. 40B–D); and in *Dendrochirus* xanthophores form a band across the central portion of the fin (Fig. 40E). The pectoral fin superficially looks larger in *Scorpaenodes* than in the other genera because the distal portion is heavily pigmented. Within *Scorpaena*, the colour of the chromatophores on the pectoral fin and whether or not they are mixed with melanophores allow species recognition (Fig. 40B–D). In *Peristedion*, the pectoral fin is also enlarged, the upper rays extremely so, and there appear to be erythrophores mixed with melanophores on the upper elongate rays (Fig. 41).

**Figure 42:**
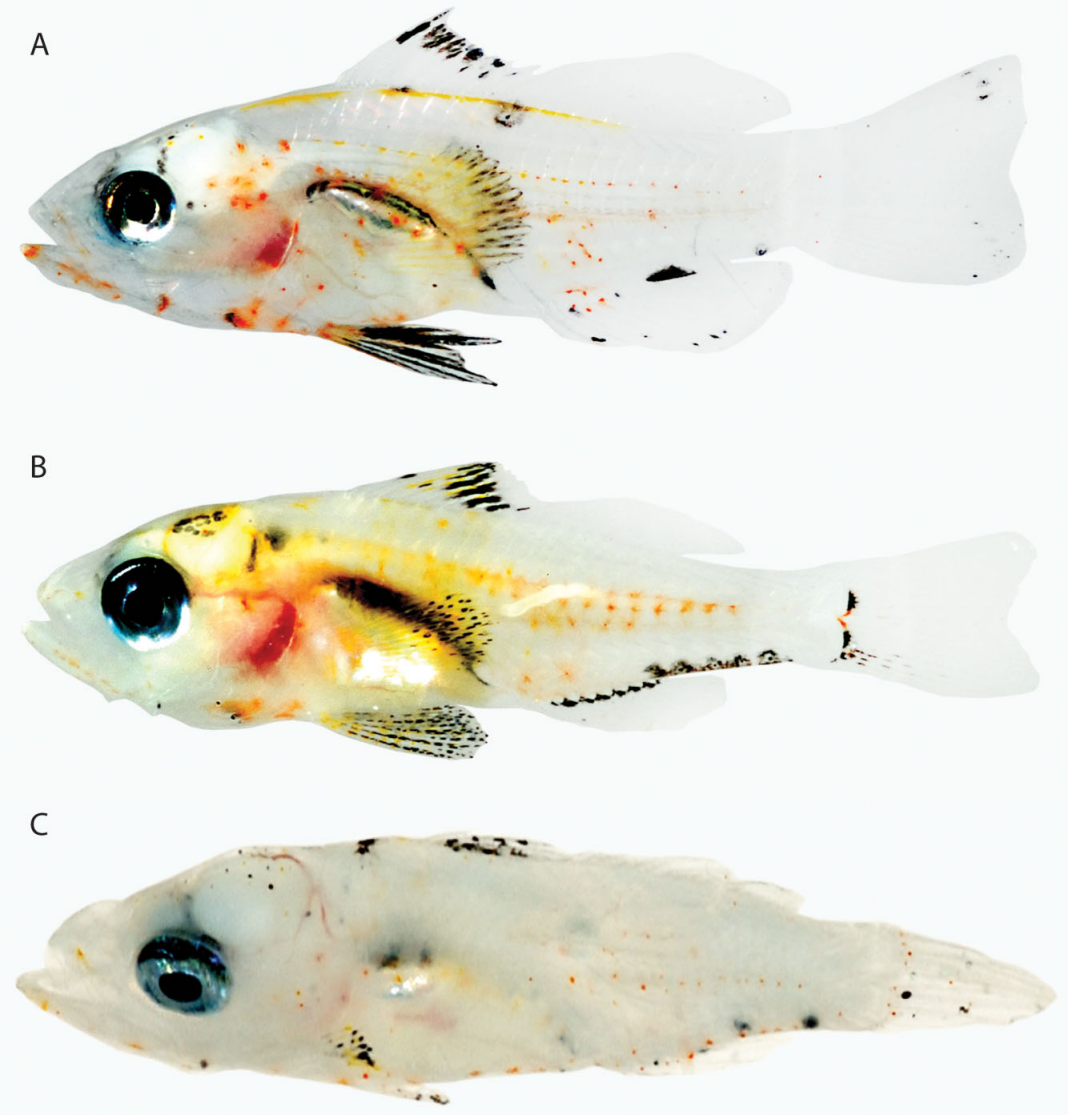
Percomorphacea (Scorpaeniformes). A, *Serranus baldwini*, 9.5 mm Standard Length (SL), BLZ 8400. B, *Diplectrum bivittatum*, 12 mm SL, BLZ 7318. C, *Hypoplectrus* sp., 5.0 mm SL, BLZ 4588. Photos A, B by Julie Mounts and David Smith; C by Julie Mounts and Carole Baldwin.

**Figure 43:**
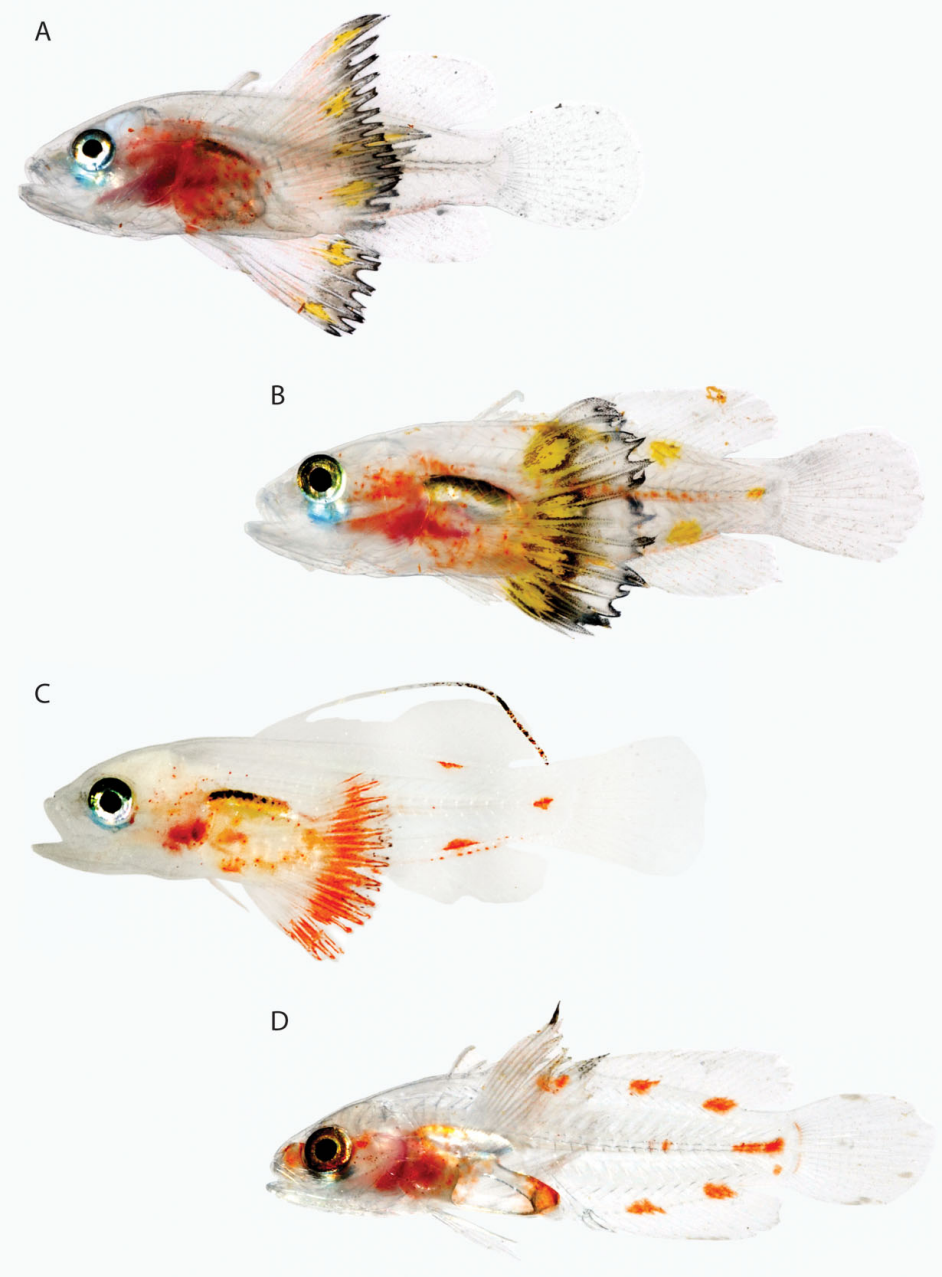
Percomorphacea (Scorpaeniformes). A, *Rypticus* sp., 12.0 mm Standard Length (SL), Belize. B, *Rypticus* sp., 11.5 mm SL, Belize. C, *Rypticus bistrispinus*, 8 mm SL, BLZ 7715. D, *Pseudogramma gregoryi*, 11 mm SL. Photos A, B, D by Julie Mounts and Carole Baldwin; C by Lee Weigt and Carole Baldwin.

*Diplectrum*, *Serranus*, and *Hypoplectrus* (serranine serranids) likewise have erythrophores or xanthophores, always mixed with melanophores, on the pectoral fin and are further similar to *Scorpaena* larvae in having yellow/orange chromatophores on the head and on the anterior and midlateral portions of the trunk (Fig. 42). The pectoral fin is not enlarged in the serranines, but it is enlarged and highly pigmented in some epinepheline serranids, including the genus *Rypticus* (Fig. 43A–C). Distinct patterns of orange/yellow chromatophores and melanophores on the enlarged fin characterize genetic lineages/species of *Rypticus*. Larvae of another epinepheline serranid examined, *Pseudogramma*, have an enlarged pectoral fin, but have only pale orange or yellow coloration vs. bright orange/yellow as in *Rypticus* (Fig. 43D). The presence of an elongate, pigmented pectoral fin is unusual among larval percomorphs examined and may be phylogenetically significant in uniting scorpaenids and serranids at the ordinal level.

**Figure 44:**
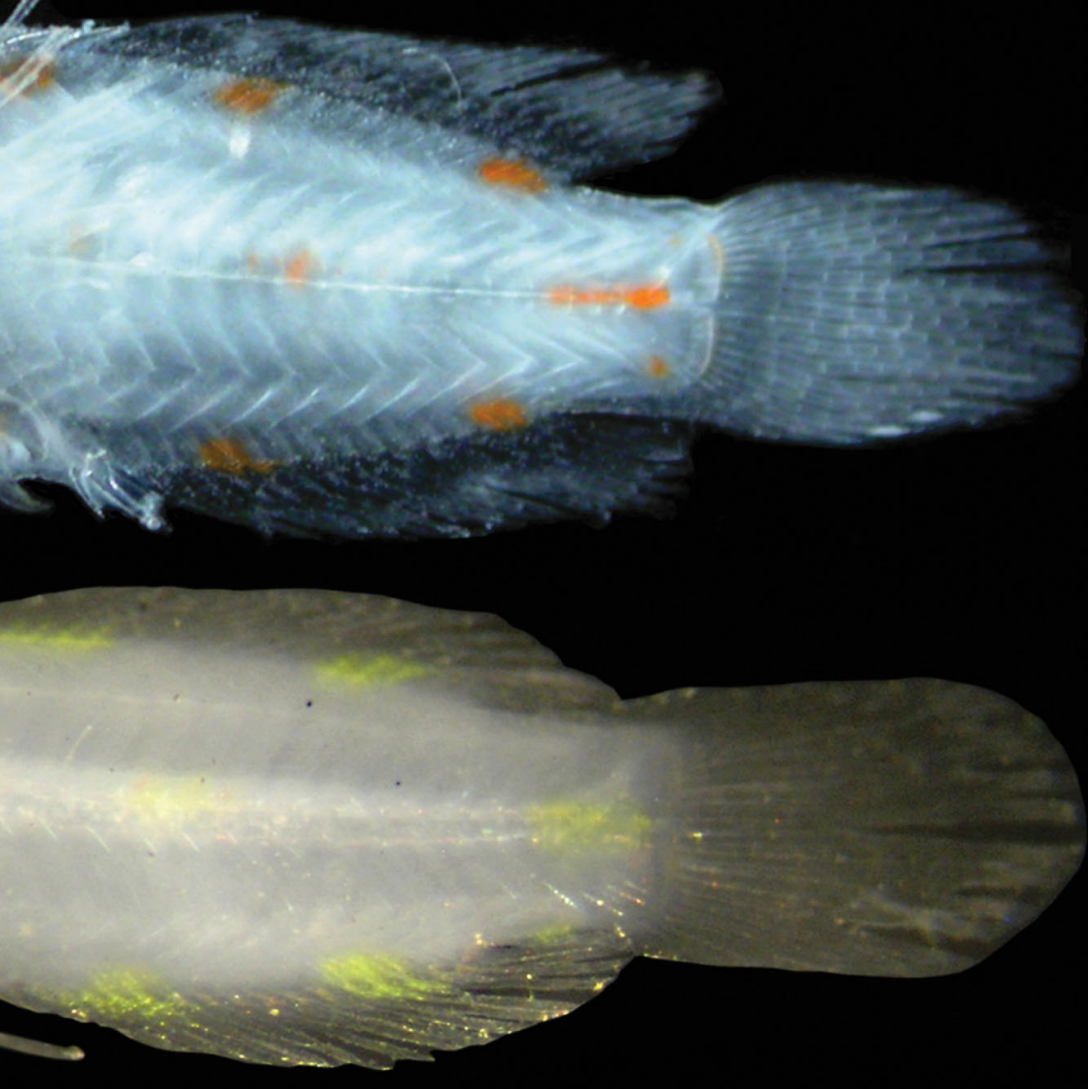
Percomorphacea (Scorpaeniformes). Top, posterior region of larval *Pseudogramma gregoryi*, Florida Straits, photo by Cedric Guigand. Bottom, posterior region of larval *Pseudogramma polyacantha*, South Africa, photo by Allan Connell.

Larval *Pseudogramma* also share with at least some *Rypticus* a similar pattern of prominent orange/yellow chromatophores on the posterior portion of the trunk: one blotch is present centrally on the caudal peduncle, and two blotches precede it, one on the dorsal portion of the trunk and one on the ventral portion (Fig. 43B–D). *Pseudogramma* and one species of *Rypticus* (Fig. 43B, D) have additional orange/yellow chromatophore blotches further anteriorly. An image of the Pacific *Pseudogramma polyacantha* shows a nearly identical colour pattern to that observed in the Atlantic *Pseudogramma gregoryi* except that the chromatophores are xanthophores rather than erythrophores (Fig. 44). Additional specimens of *Pseudogramma polyacantha* are needed to determine whether the colour is always yellow, but in multiple colour images of *Pseudogramma gregoryi* examined from Belize larval-fish collections, the colour is always orange.

**Figure 45:**
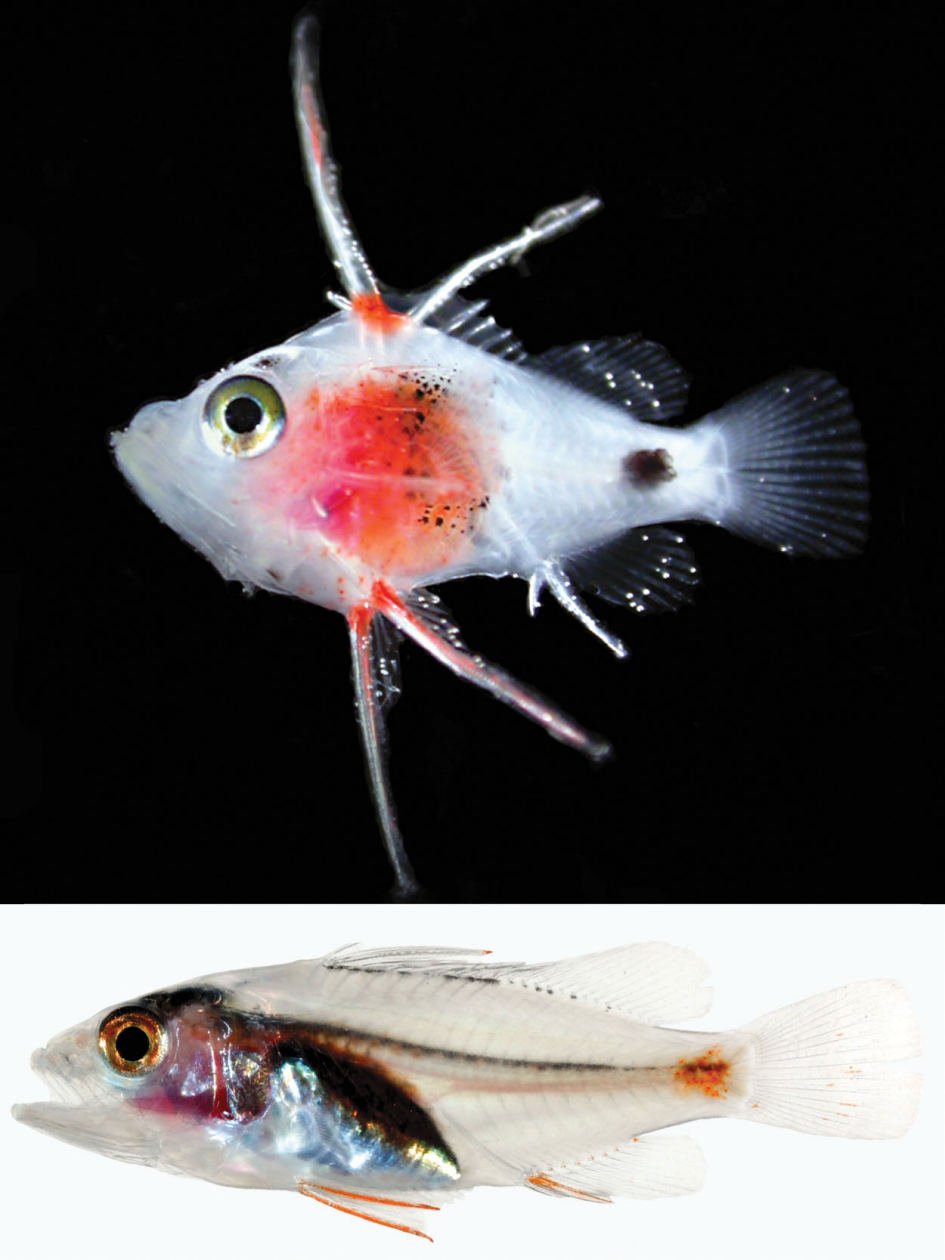
Percomorphacea (Scorpaeniformes). Top, *Gonioplectrus hispanus*, Florida Straits, photo by Cedric Guigand. Bottom, *Mycteroperca bonaci*, 21.0 mm Standard Length, Belize, photo by Julie Mounts and Carole Baldwin.

**Figure 46:**
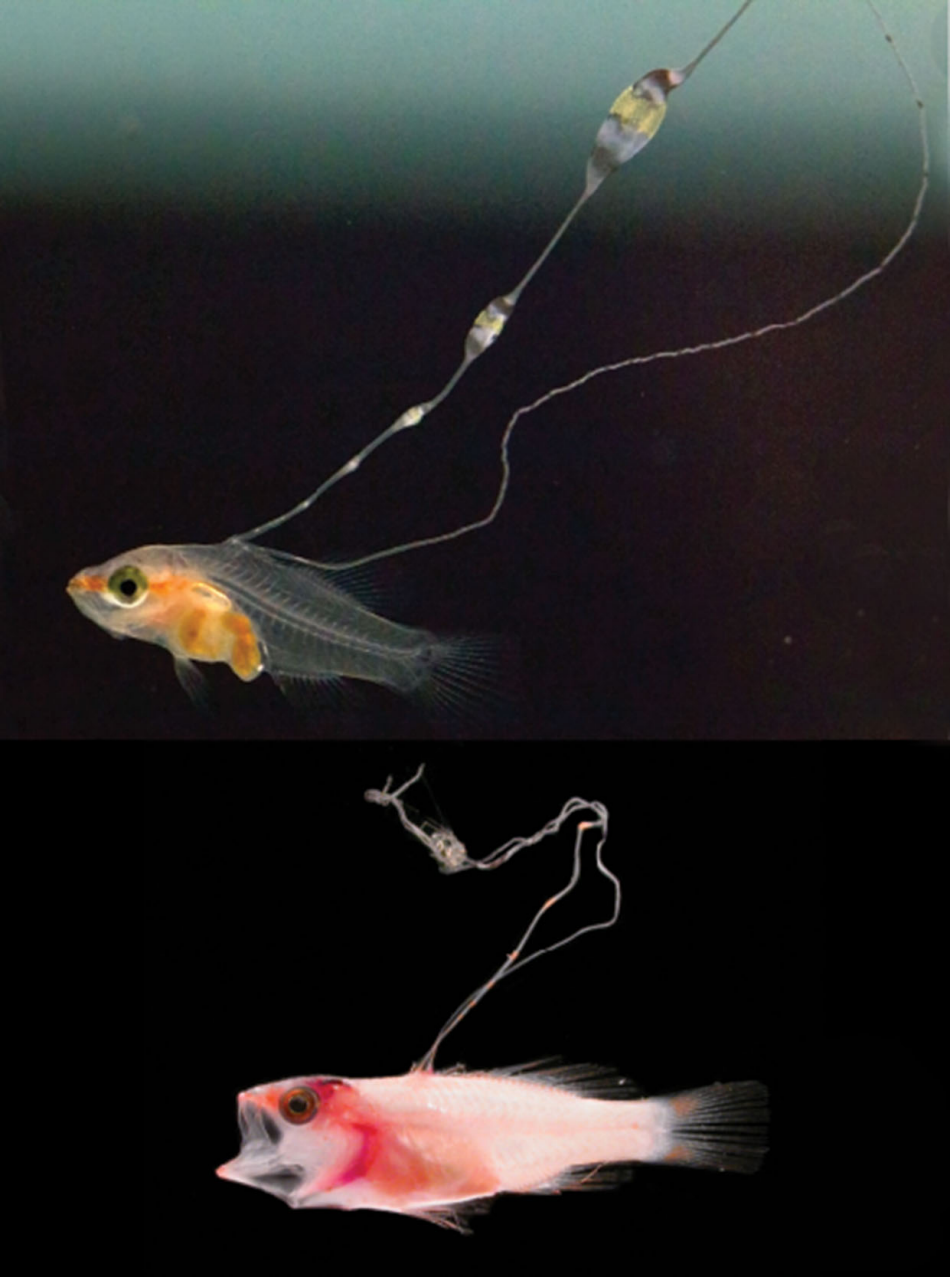
Percomorphacea (Scorpaeniformes). Top, *Liopropoma rubre*, reared, photo by Christopher Paparo. Bottom, *Bathyanthias* sp., Florida Straits, photo by Cedric Guigand.

**Figure 47:**
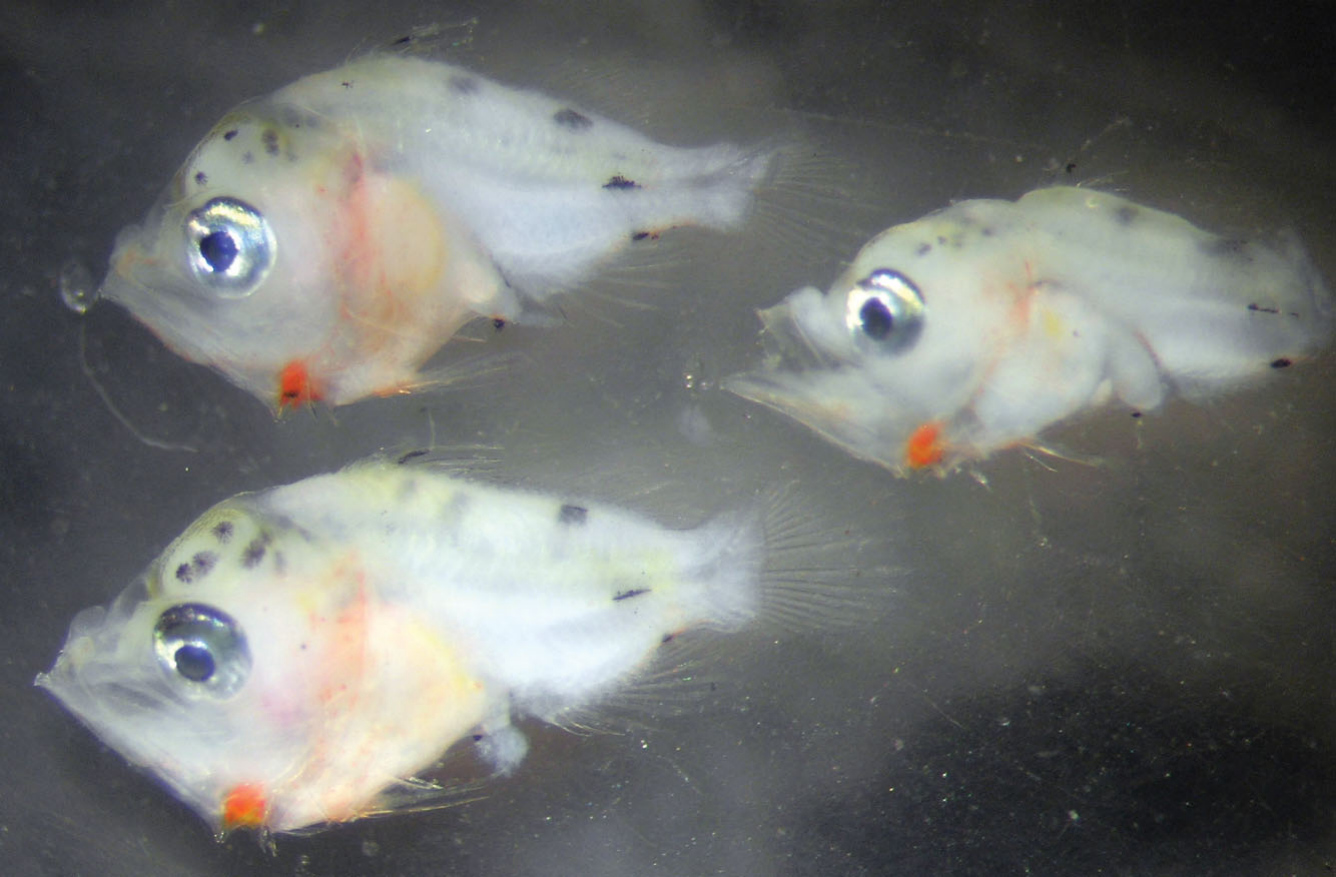
Percomorphacea (Scorpaeniformes). *Pseudanthias cooperi*, South Africa. Photo by Allan Connell.

Larvae of other epinepheline serranids examined – the Spanish flag *Gonioplectrus*, the grouper *Mycteroperca*, and the liopropomin basses *Bathyanthias* and *Liopropoma* (Figs 45, 46) – do not have enlarged pectoral fins. *Gonioplectrus* has orange pigment on and at the base of the elongate second dorsal-fin spine, in a large blotch over the opercular region and base of pectoral fin, and along the entire length of the elongate pelvic-fin spine (Fig. 45). The pelvic-fin spine is also orange in settlement-stage *Mycteroperca bonaci* larvae (Fig. 45). *Gonioplectrus* has only melanophores in a blotch on the caudal peduncle, whereas *Mycteroperca* has erythrophores in a similar arrangement and position. Larval *Bathyanthias* from the Florida Straits and reared *Liopropoma rubre* larvae have two extremely elongate dorsal-fin spines, the anterior of which has pigmented swellings along its length (Fig. 46). In *Bathyanthias*, the swellings are small and orange, whereas in *Liopropoma* they are very large, and each is encircled with a prominent band of yellow pigment. Larvae of both genera have a stripe of erythrophores from the snout to the eye and additional erythrophores on the tip of the lower jaw and on the head behind the eye. *Bathyanthias* has orange pigment on the second dorsal, caudal, anal, and pelvic fins, but this pigment is not evident in available photographs of *Liopropoma*. Larvae of one anthiine serranid, *Pseudanthias cooperi*, do not greatly resemble other serranids in colour pattern: they are mostly pale, with a bright orange blotch at the posterior end of the lower jaw, pale xanthophores on the head, and pale xanthophores and erythrophores on the trunk (Fig. 47).

#### Stromateiformes

**Figure 48:**
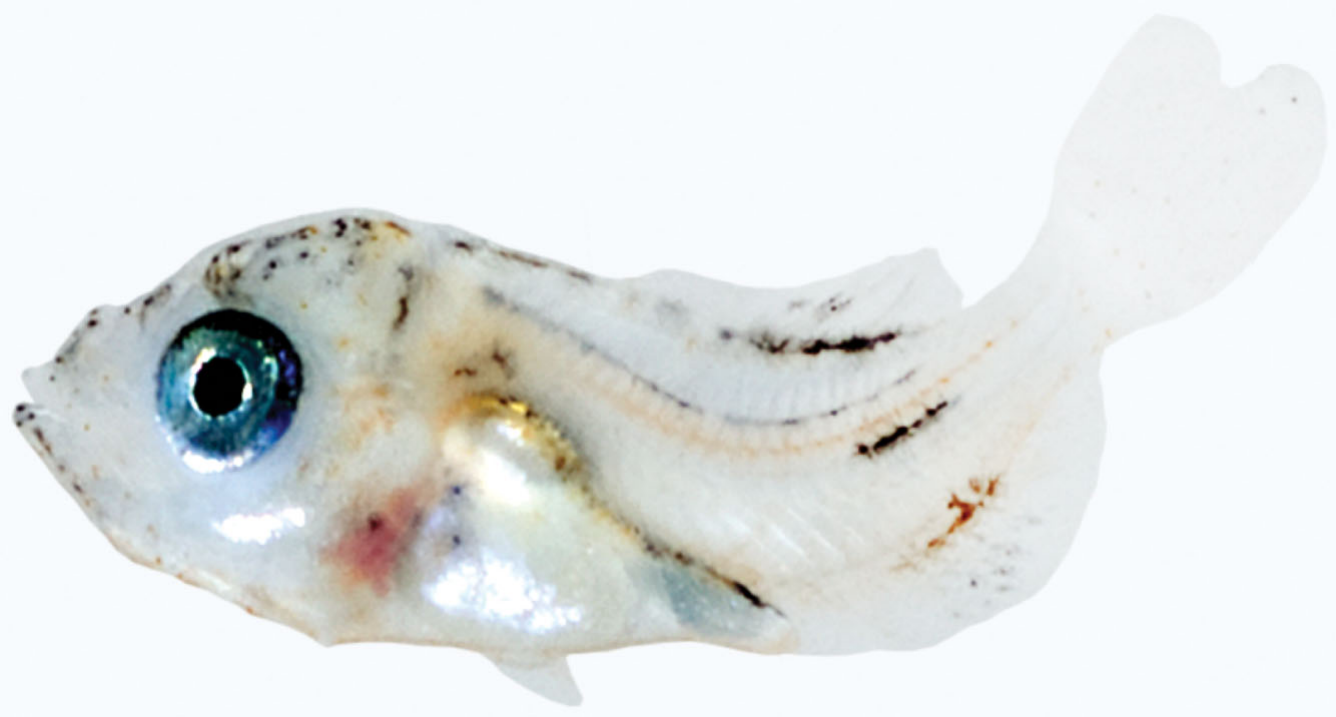
Percomorphacea (Stromateiformes). Nomeidae (*Cubiceps?*), 5.0 mm Standard Length, BLZ 6047. Photo by Lee Weigt and Carole Baldwin.

Stromateiformes are represented in the Belize material by one 5.0-mm SL unidentified specimen that resembles an illustration of a 5.1-mm SL larva of *Cubiceps pauciradiatus* (Lamkin, 2006) in pattern of melanophores. The DNA barcode for the Belize larva is approximately 3% different from that of *Cu. pauciradiatus*, which is greater than typical intraspecific divergence (Weigt *et al*., 2012). Further study is needed, but the DNA data clearly identify the larva as a nomeid. It is mostly pale, but has xanthophores mixed with chromatophores on the top of the head and in a series over the swimbladder and gut (Fig. 48). Two species of Stromateidae from South Africa (Connell, 2007) have xanthophores mixed with melanophores in preflexion stages, and one postflexion specimen has most of the head and trunk covered with xanthophores and large dark spots.

#### Tetraodontiformes

**Figure 49:**
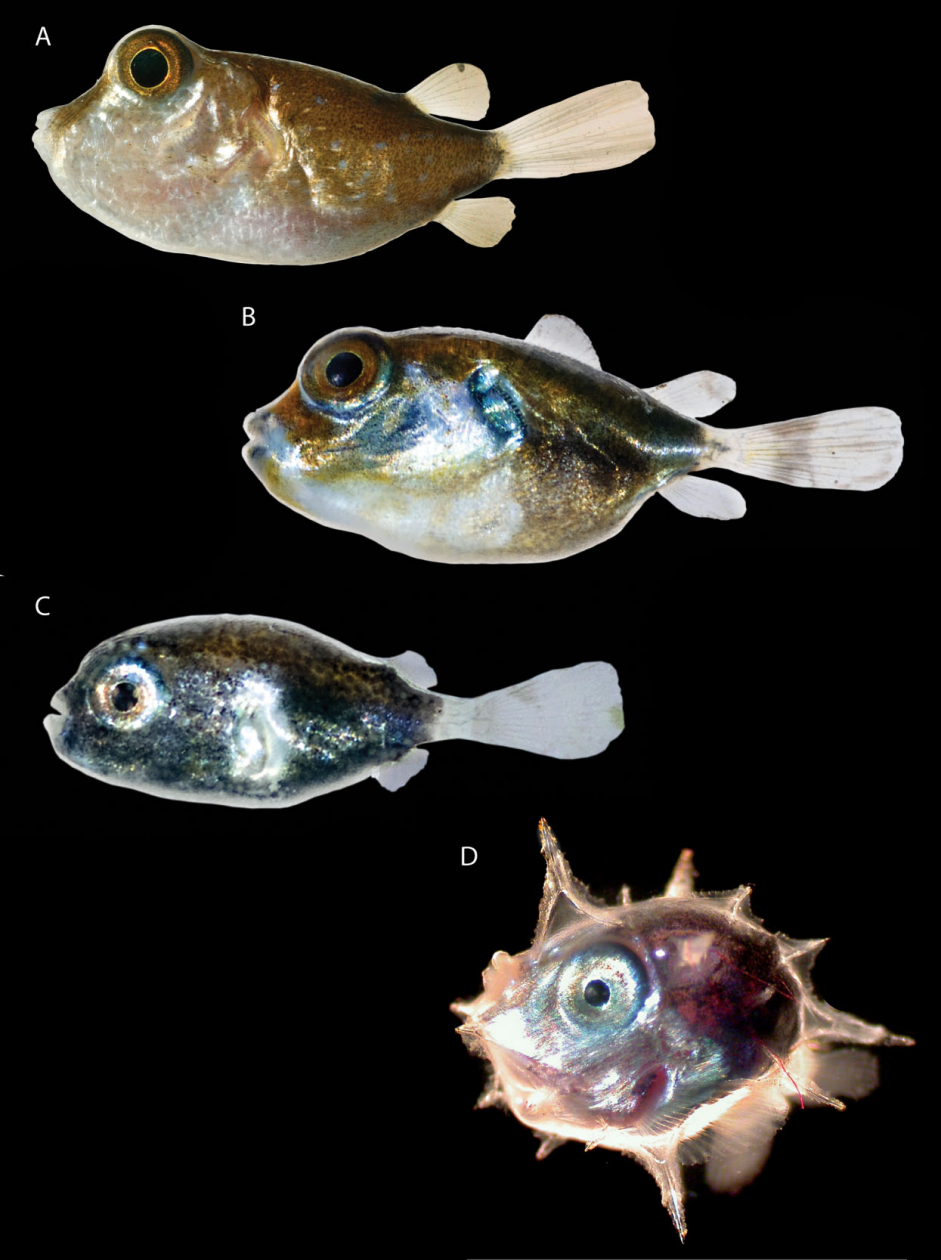
Percomorphacea (Tetraodontiformes). A, *Canthigaster rostrata*, 12.0 mm Standard Length (SL), Belize. B, *Sphoeroides spengleri*, 7.0 mm SL, BLZ 10114. C, *Sphoeroides testudineus*, 5 mm SL, BLZ 10147. D, *Ranzania laevis*, Florida Straits. Photo A by Julie Mounts and Carole Baldwin; B, C by Donald Griswold and Carole Baldwin; D by Cedric Guigand.

**Figure 50:**
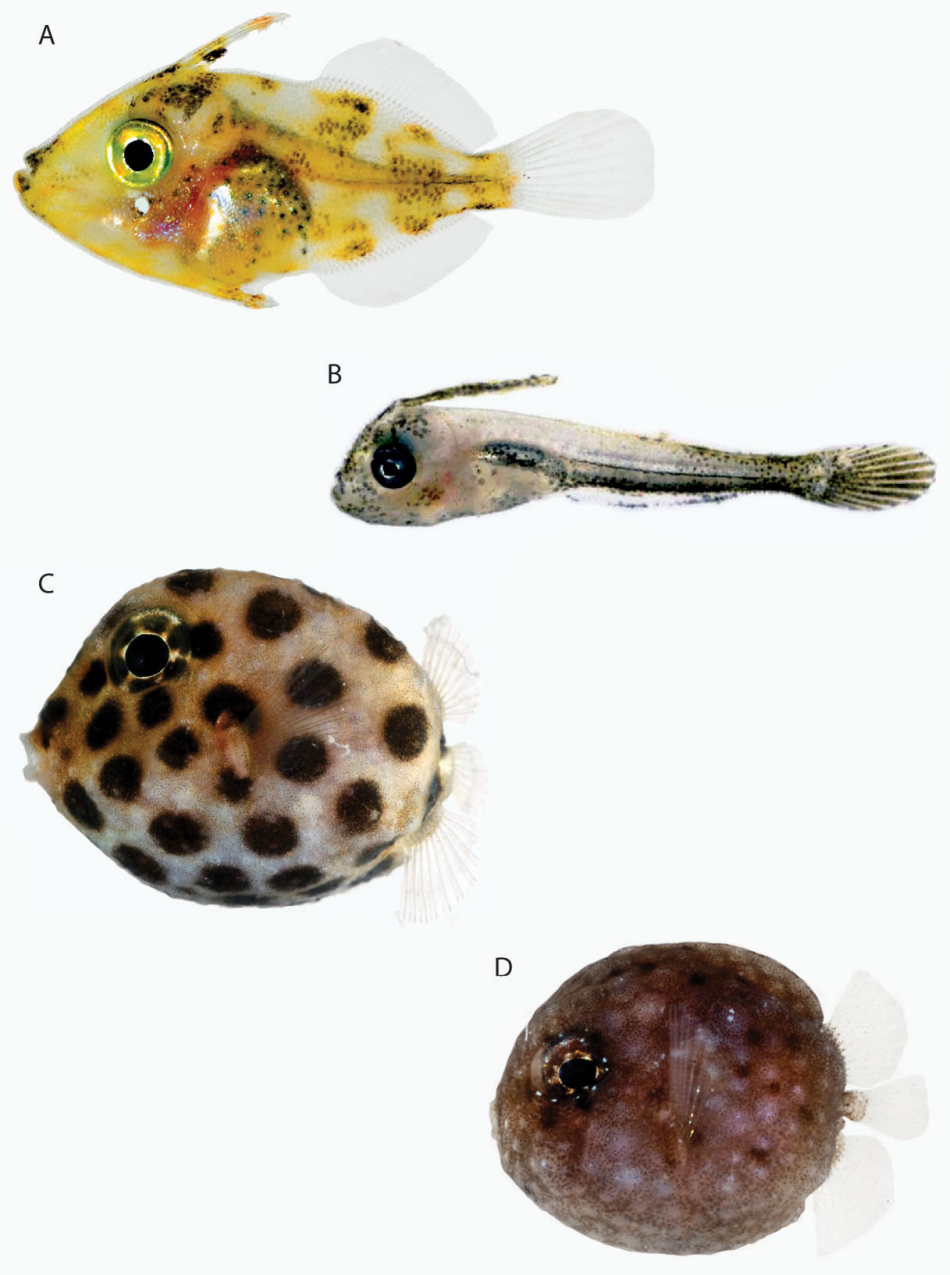
Percomorphacea (Tetraodontiformes). A, *Monacanthus ciliatus*, 8.0 mm Standard Length (SL), BLZ 10223. B, *Aluterus schoepfi*, 7.5 mm SL, Belize, USNM 353531. C, Ostraciidae, 10.0 mm SL, Belize. D, *Lactophrys trigonus*, 6.0 mm SL, BLZ 8446. Photo A by Donald Griswold and Carole Baldwin; B by David Smith; C by Julie Mounts and Carole Baldwin; D by Julie Mounts and David Smith.

Tetraodontiformes are represented in the material examined by larval monacanthids and ostraciids (Balistoidei), and tetraodontids and a molid (Tetraodontoidei). Larval *Canthigaster rostrata* and *Sphoeroides* (Tetraodontidae) have strikingly similar colour patterns in that most of the body is covered with iridophores that reflect bronze/gold/blue colours (Fig. 49A–C). A similar shimmering colour pattern is present in larval *Ranzania*, a molid (Fig. 49D). Colour pattern in larvae of these tetraodontoids, which is very different from that observed in larval ostraciids and monacanthids, may provide corroborative evidence for the monophyly of the Tetraodontoidei (*sensu* Holcroft, 2005) or a less inclusive clade within that assemblage. *Monocanthus ciliatus* larvae are mostly yellow, with xanthophores present on the head, trunk, and spinous dorsal and pelvic fins, and larval *Aluterus* is less colourful but has some yellow pigment on the head, trunk, and spinous dorsal and caudal fins (Fig. 50A, B). Larval ostraciids have a pale orange/yellow coloration overlain by dark dots or large dark spots (Fig. 50C, D). Relationships of tetraodontiforms to other acanthomorph fishes are unresolved, but taxa hypothesized to be close relatives include zeiforms, acanthuriforms, and lophiiforms (Wiley & Johnson, 2009, and references therein). There is little resemblance between tetraodontiform larvae examined and *Z. faber* (Connell, 2007; Appendix), and, as noted above (see Acanthuriformes), there is little similarity between acanthurid and tetraodontiform larvae. There is at least superficial resemblance between tetraodontid and chaetodontid/pomacentrid larvae in that the pigment is primarily in the form of iridophores (Figs. 30, 49).

**Figure 51:**
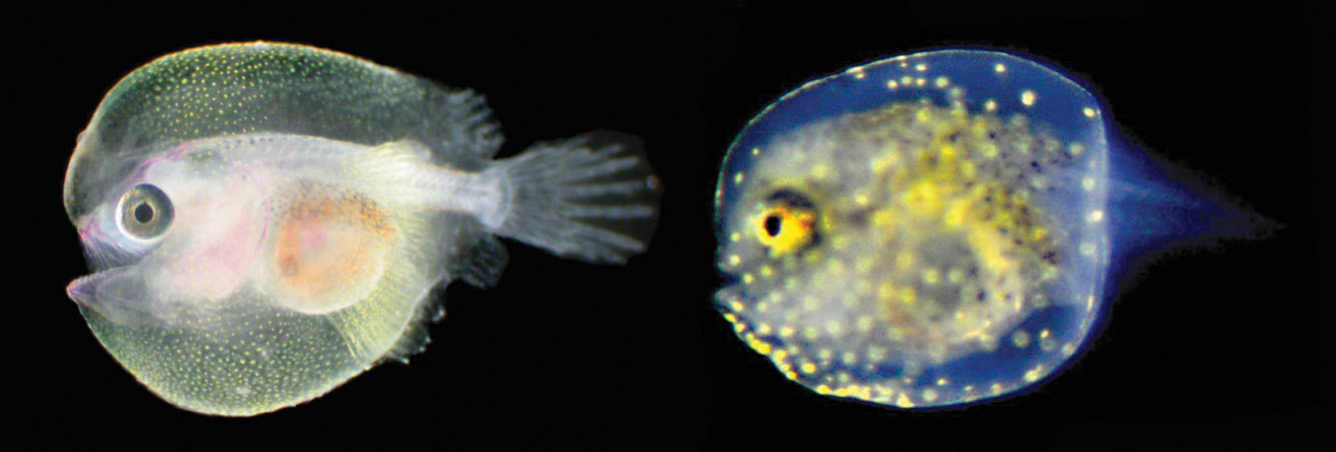
Percomorphacea (Tetraodontiformes). Left, unknown lophiiform, Florida Straits, photo by Cedric Guigand (previously published in Fogarty & Botsford [2007]). Right, Ostraciidae, South Africa, photo by Allan Connell.

The tetraodontiform/lophiiform hypothesis (Holcroft & Wiley, 2008; Miya *et al*., 2010) is intriguing in light of colour patterns and other aspects of larval morphology: compare a preflexion larval ostraciid from South Africa with an unidentified lophiiform (Fig. 51). In both, but in larvae of no other teleosts examined, the trunk is contained within an inflated, three-dimensional sac that is covered with xanthophores. Aboussouan & Leis (2004) noted the presence of a ‘dermal sac’ in tetraodontids, diodontids, molids, ostraciids, and balistids but not in triacanthodids, triacanthids, and monacanthids. Pietsch (1984: 320) described lophiid and other lophiiform larvae as having the epidermal layer of the head and body ‘greatly distended by transparent, gelatinous connective tissue’. The inflated condition in tetraodontiforms appears to be restricted to the preflexion stage, whereas that in lophiiforms persists after flexion (compare Figs 50C, D and 51; see also Connell, 2007).

#### Trachiniformes

Trachiniformes, which currently comprise the fishes traditionally included in the Trachinoidei (Chiasmodontidae, Champsodontidae, Pinguipedidae, Cheimarrhichthyidae, Trichodontidae, Creediidae, Percophididae, Leptoscopidae, Trachinidae, and Uranoscopidae) and Ammodytidae, were considered likely to be paraphyletic by Wiley & Johnson (2009). Among trachinoid larvae from off South Africa (Connell, 2007; Appendix), an early-stage larval uranoscopid bears little resemblance to an early-stage chiasmodontid and a champsodontid. The last two are similar in having only melanophores and lacking obvious nonmelanistic colour in the preflexion stage, whereas the larval uranoscopid has distinctive blotches of xanthophores. Mito (1962) described a reared series of *Champsodon snyderi* and noted that the species has only melanophores throughout larval development. Information on colour patterns in postflexion larvae of other champsodontids might be useful in commenting on the proposed relationship between champsodontids and scorpaeniforms (Mooi & Johnson, 1997). As noted (see ‘Scorpaeniformes’ above), the latter have distinctive patterns of erythrophores/xanthophores.

## Discussion

The first broad comparative survey of nonmelanistic colour in marine teleost larvae presented herein has revealed a striking array of colour patterns that may inform phylogeny at multiple taxonomic levels. The absence of xanthophores, erythrophores, and iridophores in pelagic larvae of most basal marine teleosts examined, and their presence in many basal neoteleosts, in all percomorph orders examined, and in nearly every percomorph species examined, suggest a phylogenetic trend (Fig. 1). It is premature to make specific hypotheses about the evolution of the three types of chromatophores in larvae of marine teleosts, but in the limited material examined, xanthophores are the only type of nonmelanistic chromatophore present in larval marine anguilliforms and stomiatiforms. Erythrophores appear at the level of Eurypterygia, and iridophores first appear in larval euacanthopterygians. The situation in Anguilliformes and basal neoteleosts, in which larvae of some members of an order exhibit nonmelanistic pigment but others do not, suggests that nonmelanistic chromatophores have arisen independently (or were lost) multiple times. Considerably more information on colour patterns in more larvae is needed to determine the taxonomic distribution of the various types of chromatophores among teleosts, as is investigation of the cellular basis of observed chromatophore types. In this study, presence or absence of chromatophores was assessed from visual examination of fresh specimens and colour photographs, but histological studies are needed to confirm that the presence or absence of a particular nonmelanistic pigment corresponds to the structural presence or absence of the vertebrate chromatophore unit that typically contains that pigment. Biological and physical factors that might affect the development or display of chromatophores also warrant study. For example, thyroid hormones are known to have a role in regulating larval-fish development, including pigment (e.g. Brown & Kim, 1995, and references therein). However, experimental manipulation of exogenous hormones suggests they may affect timing of pigment development and density/shape of pigment features but not the types of chromatophores that develop (Reddy & Lam, 1992; Brown & Kim, 1995; Clement, Lichtenbert & Kohler, 2001). It seems unlikely that physical factors related to habitat ecology have much bearing on the presence or absence of nonmelanistic chromatophores, as larvae of most marine teleosts inhabit a similar planktonic realm. In the material examined for this study, fish larvae such as those of clupeids, engraulids, elopiforms, and anguilliforms that lack nonmelanistic chromatophores were collected in the same plankton samples as larvae that have them.

Apomorphic characters of chromatophore patterns rather than the presence or absence of chromatophore types may have the greatest phylogenetic potential in teleosts. Pigment patterns of adults have been incorporated into phylogenetic studies of fishes at subgeneric, generic, and familial levels (e.g. Mabee, 1995; Ayache & Near, 2009; Layman & Mayden, 2012), but nonmelanistic colour patterns of marine fish larvae have not. Among the percomorphs examined, colour patterns in marine fish larvae may have phylogenetic significance at several taxonomic levels: interordinal – Mugiliformes/Beloniformes, Tetraodontiformes/Lophiiformes; interfamilial – Chaeotodontidae/Pomacentridae, Bothidae/Paralichthyidae, Chaenopsidae/Labrisomidae, Callionymidae/Gobiesocidae; familial – Apogonidae, Eleotrididae, Microdesmidae, Scaridae, Scorpaenidae, Tetraodontidae; intergeneric – *Abudefduf*/*Amblyglyphidodon*, *Anampses*/*Halichoeres*/*Thalassoma*, *Ctenogobius/Gnatholepis*/*Gobionellus*, *Nes*/*Psilotris*, *Lutjanus*/*Ocyurus*; and generic – *Bathygobius*, *Coryphopterus*, *Scorpaena*, *Rypticus*, *Xyrichtys*. Slight differences in generic colour patterns of larvae – including whether the pattern comprises xanthophores or erythrophores – often distinguish component species (e.g. those of *Xyrichtys*, *Pseudogramma*, *Scorpaena*).

Matsumoto (1965) noted that vesicles that contain yellow and red pigments are sometimes found in the same cell in freshwater *Xiphophorus*, suggesting that the distinction between xanthophores and erythrophores is artificial. The presence in some marine genera of species with nearly identical patterns of pigment except for the colour of the chromatophores (orange or yellow) suggests that xanthophores and erythrophores are distinct. Erythrophores in fishes reportedly are red/orange because of carotenoids ingested in the diet, whereas the yellow pigment of xanthophores (from pteridines) is metabolized endogenously (Grether *et al*., 2004, and references therein). Whether the origins of yellow and orange pigments in marine fish larvae also are endogenous and exogenous, respectively, does not appear to have been investigated. It seems remarkable that nearly identical patterns of pigment that differ only in colour (e.g. *Xyrichtys*, Fig. 26A, C; *Pseudogramma*, Fig. 44) would have different biochemical origins. Future studies that investigate the biochemistry of pigments in marine fish larvae might also compare the chemistry of pigments in basal teleosts such as eel leptocephali, basal neoteleosts such as whalefish, and percomorphs. Similarities or differences in the biochemistry of the pigments may shed light on the homology and evolutionary history of larval-fish pigments among marine teleosts.

**Figure 52:**
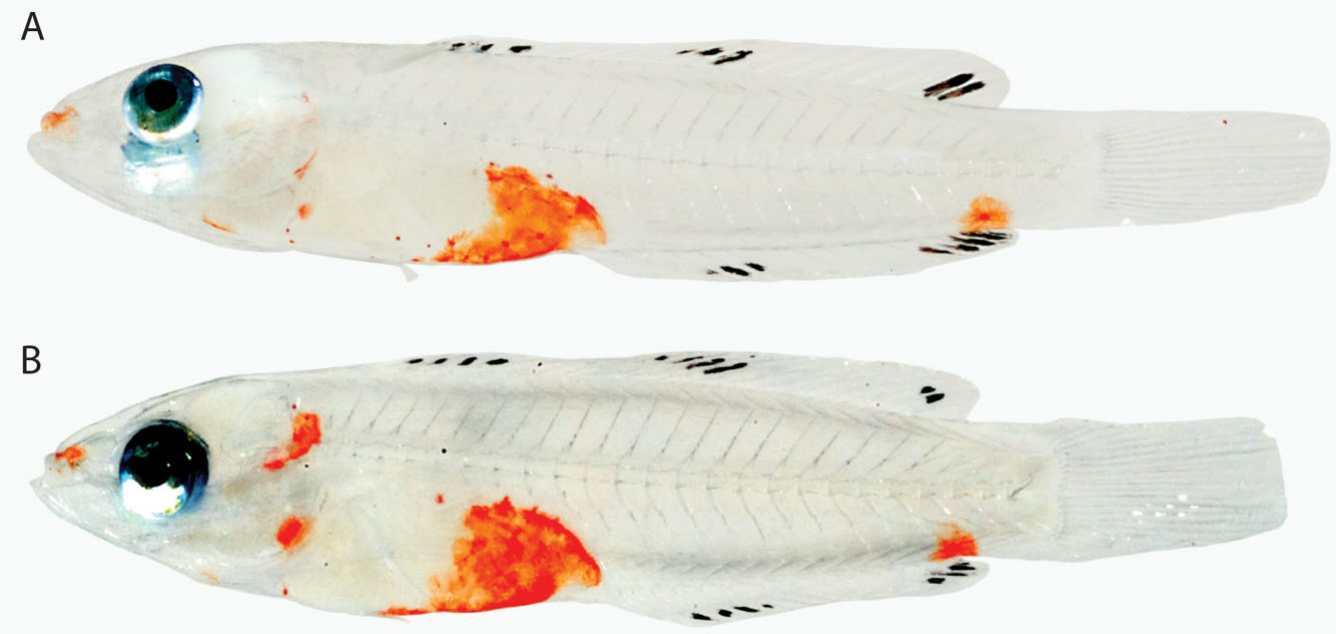
Intraspecific variation in pattern of erythrophores in *Halichoeres bivittatus*. A, 12.5 mm Standard Length (SL), BLZ 6426. B, 12.5 mm SL, BLZ 6424. Note that the pattern of orange pigment is nearly identical in the two specimens but that in BLZ 6426 the erythrophores in the vertical series behind the head appear to be contracted. Photos by Julie Mounts and David Smith.

Kendall *et al*. (1984) and Mabee (1995) noted that individual variation and convergent evolution of some patterns are considered obstacles for phylogenetic use of pigment characters because of difficulties in establishing hypotheses of homology. Numerous specimens of many species were examined in this study (Appendix), and individual variation in nonmelanistic chromatophore patterns is low. Differences frequently appear to reflect the expanded or contracted state of the chromatophores rather than their pattern (Fig. 52). Assessing homology of nonmelanistic chromatophores in larvae among major groups of marine teleosts through phylogeny is complicated by the absence of a comparable, transient, pigment phase in most freshwater fishes. Larvae of marine Clupeiformes examined, for example, lack nonmelanistic chromatophores, but most Otomorpha are freshwater inhabitants. The presence of xanthophores and iridophores in larval cypriniforms such as freshwater *Danio* is well documented (Johnson *et al*., 1995; Parichy *et al*., 2000; Parichy, 2003, 2006). What is the relationship between the pigment pattern of larval freshwater fishes and that of pelagic marine fish larvae? As shown in the ontogenetic series of numerous marine teleosts photographed by Connell (2007), the pigment pattern of fully developed marine fish larvae is preceded by a very different pigment phase in recently hatched larvae. For example, a reared myctophid series shows yellow pigment on the head and body in yolksac larvae, but all yellow pigment has disappeared by 4.3 mm NL. Acanthurid acronurus larvae examined have iridophores but no erythrophores or xanthophores, yet preflexion *Acanthurus mata* exhibit xanthophores (see ‘Acanthuriformes’ above). Studies on aquarium-reared callionymids revealed a colour transformation in larval *Syn. splendidus* from yellow in the preflexion stage to bright orange after flexion (Fig. 19; Wittenrich *et al*., 2010). Possibly the early pigment pattern in marine fish larvae is the ontogenetic counterpart of the larval pigment pattern in freshwater fishes. Assembling ontogenetic series of marine fishes that incorporate chromatophore patterns from recently hatched to adult stages, investigating the development of the pattern at each stage, and comparing the development of pigment among marine and freshwater fishes would shed light on the homology of pigment patterns among life-history stages and among related taxa. Such ontogenetic series also warrant further study as a potential source of phylogenetic characters. Mabee (1995) highlighted the phylogenetic significance of pigment-pattern development in the evolution of centrarchid sunfishes, which are freshwater fishes that exhibit direct development of the adult pigment pattern from the early larval stage. The ontogeny of pigment patterns in marine fishes may be an even riper source of phylogenetic information yet to be tapped. In addition to the colour transitions in myctophids, acanthurids, and callionymids mentioned above, the ontogenetic transition in mugilids and some beloniforms from a stage dominated by xanthophores, erythrophores, and melanophores to one dominated by iridophores is an example. This transition also suggests that the presence of iridophores may be homologous in the two groups because the condition was derived from a similar ontogenetic transformation. Incorporating developmental studies of colour patterns in future phylogenetic treatments of the topic could thus provide both crucial information on character homology and new phylogenetic characters.

Yasir & Qin (2007) described developmental changes in pigment patterns of the marine anemonefish *Amphiprion ocellaris* from embryo to larva and noted that a better understanding of the process of pigment formation is needed. Embryologically, neural crest cells, which give rise to all four types of chromatophores, migrate to the dermis, and the density and distribution of the chromatophores are integrated to produce colour patterns in vertebrates (Hawkes, 1974; Bagnara, 1987; Hall, 1999). Holt (2011) noted that the pigment patterns of fishes are formed during embryogenesis and are replaced by the adult pattern at metamorphosis. Studies of freshwater *Danio* indicate that the adult colour pattern results from incorporation of both embryonic chromatophores and differentiation of new chromatophores from stem cells at metamorphosis, and the ratio of contribution by the two may vary significantly among species (Parichy, 2003, 2006). The existence of an additional colour phase (between the recently hatched and adult phases) in pelagic larvae of marine fishes would appear to complicate this scenario. Furthermore, juveniles of many marine fishes exhibit yet another colour phase that is different from the pelagic larval and adult phases (e.g. Haemulidae, Labridae, Pomacentridae, Pomacanthidae). The ontogeny of chromatophore patterns has been studied in few marine fishes, but Nakamura *et al*. (2010 and references therein) have investigated development of the adult pigment pattern in Pleuronectiformes. Two types of melanophores and xanthophores/erythrophores are hypothesized to exist in pleuronectiforms – those that develop in the larval stage prior to metamorphosis and those that develop afterwards and form the adult pigment pattern. Flatfishes exhibit an unusual ontogeny in which larvae are bilaterally symmetrical, including the pigment pattern, but metamorphosis involves migration of one eye to the other side of the head resulting in a bilaterally asymmetrical juvenile and adult. There is typically little or no pigment on the ‘blind’ side but the ‘eyed’ side is well pigmented. Whether chromatophores of pelagic larvae in other marine fishes are succeeded ontogenetically by adult versions of those pigment cells is unknown, but this would be a reasonable hypothesis based on the different pigment patterns exhibited in larval and adult stages. Other features that are present in pelagic larvae of some marine fishes, such as elongate fin rays and head spines, disappear or transform upon metamorphosis to the juvenile stage. Studies in marine fishes that would explain the origin of adult pigment patterns (i.e. from existing neural crest lineages or newly differentiated stem cells) are lacking.

If the colour patterns exhibited by marine fish larvae are present only during the pelagic larval period, are they adaptations that have functional significance? Grether *et al*. (2004) discussed the potential significance of the different pigments in adult freshwater fishes, but only one of their hypotheses seems potentially relevant to pelagic marine fish larvae – ‘spectral fine-tuning’. Grether *et al*. (2004) noted that orange and yellow pigments have different absorptive properties that may enable spectral fine-tuning, which, in turn, may enhance the ability to blend into the background. Orange coloration is not restricted to fishes in a plankton sample: numerous invertebrates such as the larvae of shrimps and crabs look very similar. Indeed, the guts of numerous freshly caught marine fish larvae are orange because of diet (see Figs 27D, 39D, 41, 46). Carvalho, Zuanon & Sazima (2006) provided examples of transparency and similar colour patterns in freshwater fishes and crustaceans that travel as a group and suggested that these organisms may be utilizing a type of protective association known as numerical mimicry as a means of avoiding potential predators. Regarding the bright orange coloration in larvae of the mandarinfish, *Syn. splendidus*, Wittenrich *et al*. (2010) noted that Lindquist (2002) and Young & Bingham (1987) hypothesized that orange may be an aposematic warning colour in the larvae of some marine organisms.

In summary, colour patterns in marine teleost larvae emerge as an intriguing new source of potentially valuable phylogenetic information, but considerably more data are needed to fully assess their significance. An increased global focus on documenting colour patterns in larvae prior to preservation would facilitate incorporating information from larval colour patterns into more systematic studies, and developmental, histological, and biochemical studies that shed light on the ontogeny of colour patterns in early life history stages would improve our understanding of ontogenetic pigment phases, characters, character transformations, and character homology.

## Appendix

Teleost larvae for which images were examined for colour patterns. ‘N’ in the ‘Colour’ column indicates no xanthophores, erythrophores or iridophores; ‘Y’ indicates the presence of any of those types of chromatophores alone or in any combination. The table is colour-coded by teleost cohort: blue indicates Clupeomorpha, green Elopomorpha, purple Euteleosteomorpha.

**Table:**

Order	Family	Genus	Species	Colour	Image
Clupeiformes	Clupeidae	*Harengula*	*humeralis*	N	BLZ 5175
Clupeiformes	Clupeidae	*Harengula*	*clupeola*	N	Fig. 3
Clupeiformes	Clupeidae	*Jenkensia*	*lamprotaenia*	N	Fig. 3 and other BLZ images
Clupeiformes	Clupeidae	*Jenkensia*	sp.	N	BLZ 8418
Clupeiformes	Engraulidae	*Anchoa*	*cayorum*	N	BLZ 8468
Clupeiformes	Engraulidae	*Anchoa*	sp.	N	Fig. 3 and other BLZ images
Albuliformes	Albulidae	*Albula*	*vulpes*	N	Fig. 2 and other BLZ images
Anguilliformes	Chlopsidae	Chilorhinus	*seunsoni*	N	Fig. 2 and other BLZ images
Anguilliformes	Moringuidae	*Moringua*	*edwardsi*	N	Fig. 2
Anguilliformes	Muraenidae	*Gymnothorax*	*moringa*	N	Fig. 2
Anguilliformes	Muraenidae	*Strophidion*	*ui*	Y	Tawa *et al*. (2012)
Anguilliformes	Muraenidae	Unknown	Unknown	Y	Fig. 5 and Miller (2009)
Anguilliformes	Nettastomatidae	*Saurenchelys*	sp.	Y	Fig. 5 and Miller (2009)
Anguilliformes	Ophichthidae	*Aprognathodon*	*platyventris*	N	Fig. 2 and other BLZ images
Anguilliformes	Ophichthidae	*Myrichthys*	*breviceps*	Y	Fig. 4 and other BLZ images
Anguilliformes	Ophichthidae	*Myrophis*	*punctatus*	N	Fig. 2 and other BLZ images
Anguilliformes	Ophichthidae	*Myrophis*	*platyrhynchus*	N	Fig. 2 and other BLZ images
Anguilliformes	Ophichthidae	*Ahlia*	*egmontis*	N	Fig. 2 and other BLZ images
Anguilliformes	Ophichthidae	Unknown	Unknown	Y	Fig. 4 and Miller *et al*. (2010)
Anguilliformes	Ophichthidae	Unknown	Unknown	Y	Fig. 5 and Miller (2009)
Anguilliformes	Ophichthidae	*Neenchelys*	sp.	Y	Fig. 5 and Miller (2009)
Anguilliformes	Ophichthidae	Unknown	Unknown	Y	Fig. 5 and Miller (2009)
Elopiformes	Elopidae	*Elops*	*saurus*	N	Fig. 2
Elopiformes	Megalopidae	*Megalops*	*atlantica*	N	Fig. 2
Stomiatiformes	Melanostomiatidae	*Opostomias*	*micipnus*	Y	http://www.fisheggsandlarvae.com/FIA2%20Melanostomiidae.htm
Stomiatiformes	Phosichthyidae	*Vinciguerria*	*nimbaria*	N	http://www.fisheggsandlarvae.com/DIIIA1%20Phosichthyidae.htm
Aulopiformes	Synodontidae	*Saurida*	sp.	N	Fig. 6 and other BLZ images
Aulopiformes	Synodontidae	*Saurida*	sp.	N	Fig. 6 and other BLZ images
Aulopiformes	Synodontidae	*Synodus*	*foetens*	N	BLZ 6429
Aulopiformes	Synodontidae	*Synodus*	*synodus*	N	Fig. 6 and other BLZ images
Aulopiformes	Synodontidae	*Trachinocephalus*	*myops*	N	BLZ 7061
Aulopiformes	Bathypteroidae?	Unknown	Unknown	Y	Fig. 7
Aulopiformes	Giganturidae	*Gigantura*	sp.	N	Fig. 7
Myctophiformes	Myctophidae	Unknown	Unknown	Y	http://www.fisheggsandlarvae.com/CLIIA2%20Myctophidae.htm
Myctophiformes	Unknown	Unknown	Unknown	N	Fig. 7
Lampridiformes	Trachipteridae?	Unknown	Unknown	Y	Fig. 8
Lampridiformes	Lampridae	*Lampris*	*guttatus*	Y	Fig. 8
Gadiformes	Bregmacerotidae	*Bregmaceros*	sp.	N	Fig. 6 and other BLZ images
Gadiformes	Gadidae	Unknown	Unknown	Y	http://www.fisheggsandlarvae.com/LIIIF1%20Gadidae.htm
Beryciformes	Berycidae	*Centroberyx*	sp.	Y	http://www.fisheggsandlarvae.com/EIIA3%20Berycidae.htm
Beryciformes	Berycidae	Unknown	Unknown	Y	Fig. 9
Beryciformes	Holocentridae	*Myripristis*	*berndti*	Y	http://www.fisheggsandlarvae.com/EIIIB6A%20Myripristis.htm
Beryciformes	Holocentridae	Unknown (2)	Unknown (2)	Y	Fig. 9A, B
Beryciformes	Holocentridae	Unknown	Unknown	Y	Fig. 9
Stephanoberyciformes	Cetomimidae	Unknown	Unknown	Y	Fig. 10
Zeiformes	Zeidae	*Zeus*	*faber*	Y	http://www.fisheggsandlarvae.com/LIA3%20Zeus%20faber.htm
Acanthuriformes	Acanthuridae	*Acanthurus*	*chirurgus*	Y	Fig. 14 and other BLZ images
Acanthuriformes	Acanthuridae	*Acanthurus*	*bahianus*	Y	Fig. 14 and other BLZ images
Acanthuriformes	Acanthuridae	Unknown	Unknown	Y	Fig. 15
Acanthuriformes	Acanthuridae	*Acanthurus*	*mata*	Y	http://www.fisheggsandlarvae.com/LIIIE7%20Acanthuridae.htm
Atheriniformes	Atherinidae	*Atherinomorus*	*stipes*	Y	Fig. 13 and other BLZ images
Atheriniformes	Atherinidae	Unknown	Unknown	Y	Fig. 13
Beloniformes	Belonidae	Unknown	Unknown	Y	BLZ, 18 mm SL Photo 2-23-2004.tif
Beloniformes	Belonidae	*Platybelone*	*argalus*	Y	Fig. 12 and other BLZ images
Beloniformes	Exocoetidae	Unknown	Unknown	Y	BLZ 7036
Beloniformes	Exocoetidae	*Prognichthys*	*occidentalis*	Y	Fig. 11
Beloniformes	Hemirhamphidae	*Hemirhamphus*	*brasiliensis*	Y	Fig. 12
Beloniformes	Hemirhamphidae	*Oxyporhamphus*	*micropterus*	Y	http://www.fisheggsandlarvae.com/CHIA1%20Oxyporhamphus.htm
Blenniiformes	Chaenopsidae	*Acanthemblemaria*	*aspera*	Y	BLZ 6046, 8449 and others
Blenniiformes	Chaenopsidae	*Acanthemblemaria*	*greenfieldi*	Y	Fig. 16 and other BLZ images
Blenniiformes	Labrisomidae	*Labrisomus*	*cricota*	Y	Fig. 16 and other BLZ images
Blenniiformes	Labrisomidae	*Labrisomus*	*haitiensis*	Y	BLZ 4513, 4360 and others
Blenniiformes	Labrisomidae	*Labrisomus*	*gobio*	Y	BLZ 8447
Blenniiformes	Labrisomidae	*Labrisomus*	*bucciferus*	Y	Fig. 16 and other BLZ images
Blenniiformes	Labrisomidae	*Malacoctenus*	*macropus*	Y	BLZ 8416, 7021 and others
Blenniiformes	Labrisomidae	*Malacoctenus*	*triangulatus*	Y	Fig. 16 and other BLZ images
Blenniiformes	Labrisomidae	*Paraclinus*	*fasciatus*	Y	Fig. 16 and other BLZ images
Caproiformes	Caproidae	*Antigonia*	*rubescens*	Y	http://www.fisheggsandlarvae.com/LIIIE4%20Caproidae.htm
Carangiformes	Carangidae	*Trachinotus*	*falcatus*	Y	Fig. 17
Carangiformes	Carangidae	*Selene*	*vomer*	Y	Fig. 18
Carangiformes	Carangidae	*Seriola*	sp.	Y	http://www.fisheggsandlarvae.com/EIIA2%20Carangidae.htm
Carangiformes	Coryphaenidae	*Coryphaena*	*hippurus*	Y	Fig. 18
Gasterosteiformes	Aulostomidae	*Aulostomus*	*chinensis*	Y	http://www.fisheggsandlarvae.com/LIIA3%20Aulostomidae.htm
Gasterosteiformes	Fistularidae	*Fistularia*	sp.	Y	http://www.fisheggsandlarvae.com/HIA3%20Fistularia.htm
Gasterosteiformes	Centriscidae	*Aeoliscus*	*punctulatus*	Y	http://www.fisheggsandlarvae.com/LIIA5%20Centriscidae.htm
Gasterosteiformes	Syngnathidae	Unknown	Unknown	Y	BLZ 6408
Gasterosteiformes	Syngnathidae	*Cosmocampus*	*albirostris*	Y	Fig. 12 and other BLZ images
Gasterosteiformes	Syngnathidae	*Cosmocampus*	*elucens*	Y	BLZ 10222, 10160 and others
Gasterosteiformes	Syngnathidae	*Penetopteryx*	*nanus*	Y	Fig. 12 and other BLZ images
Gobiesociformes	Callionymidae	*Callionymus*	*bairdi*	Y	Fig. 19 and other BLZ images
Gobiesociformes	Callionymidae	*Callionymus*	*marleyi*	Y	Fig. 19
Gobiesociformes	Callionymidae	*Draculo*	*celetus*	Y	http://www.fisheggsandlarvae.com/CDIIIA1%20Callionymidae.htm
Gobiesociformes	Callionymidae	*Synchiropus*	*splendidus*	Y	Fig. 19 and Wittenrich *et al*. (2010)
Gobiesociformes	Gobiesocidae	*Acyrtops*	*beryllinus*	Y	Fig. 19
Gobiiformes	Eleotrididae	*Erotelis*	sp.	Y	Fig. 20 and other BLZ images
Gobiiformes	Gobiidae	*Bathygobius*	*lacertus*	Y	Fig. 22
Gobiiformes	Gobiidae	*Bathygobius*	*soporator*	Y	Fig. 22 and other BLZ images
Gobiiformes	Gobiidae	*Bathygobius*	*curacao*	Y	Fig. 22 and other BLZ images
Gobiiformes	Gobiidae	*Coryphopterus*	*personatus*	Y	Fig. 22 and other BLZ images
Gobiiformes	Gobiidae	*Coryphopterus*	*tortugae*	Y	Fig. 22 and other BLZ images
Gobiiformes	Gobiidae	*Coryphopterus*	*glaucofraenum*	Y	BLZ 5225
Gobiiformes	Gobiidae	*Coryphopterus*	*venezuelae*	Y	Fig. 22 and other BLZ images
Gobiiformes	Gobiidae	*Coryphopterus*	*eidolon*	Y	BLZ 5318
Gobiiformes	Gobiidae	*Coryphopterus*	*kuna*	Y	Fig. 22 and other BLZ images
Gobiiformes	Gobiidae	*Ctenogobius*	*saepepallens*	Y	Fig. 21 and other BLZ images
Gobiiformes	Gobiidae	*Gnatholepis*	*thompsoni*	Y	Fig. 21 and other BLZ images
Gobiiformes	Gobiidae	*Gobionellus*	*oceanicus*	Y	Fig. 21 and other BLZ images
Gobiiformes	Gobiidae	*Nes*	*longus*	Y	Fig. 21 and other BLZ images
Gobiiformes	Gobiidae	*Priolepis*	*hipoliti*	Y	Fig. 22 and other BLZ images
Gobiiformes	Gobiidae	*Psilotris*	sp.	Y	Fig. 21 and other BLZ images
Gobiiformes	Gobiidae	*Psilotris*	sp.	Y	BLZ 6015
Gobiiformes	Microdesmidae	*Cerdale*	*floridana*	Y	Fig. 20 and other BLZ images
Gobiiformes	Microdesmidae	*Microdesmus*	*carri*	Y	Fig. 20 and other BLZ images
Gobiiformes	Microdesmidae	*Microdesmus*	*bahianus*	Y	Fig. 20 and other BLZ images
Gobiiformes	Microdesmidae	*Ptereleotris*	*helenae*	Y	Fig. 20 and BLZ 5444
Labriformes	Labridae	*Anampses*	*lineatus*	Y	Connell pers. comm.
Labriformes	Labridae	*Doratonotus*	*megalepis*	Y	Fig. 26 and other BLZ images
Labriformes	Labridae	*Halichoeres*	*bivittatus*	Y	Fig. 25 and other BLZ images
Labriformes	Labridae	*Halichoeres*	*poeyi*	Y	Fig. 25 and other BLZ images
Labriformes	Labridae	*Halichoeres*	*garnoti*	Y	Fig. 25 and other BLZ images
Labriformes	Labridae	*Halichoeres*	*maculipinna*	Y	Fig. 25 and other BLZ images
Labriformes	Labridae	*Lachnolaimus*	*maximus*	Y	Fig. 26 and other BLZ images
Labriformes	Labridae	*Thalassoma*	*bifasciatum*	Y	Fig. 25 and other BLZ images
Labriformes'	Labridae	*Xyrichtys*	*martinicensis*	Y	Fig. 26 and other BLZ images
Labriformes	Labridae	*Xyrichtys*	*splendens*	Y	Fig. 26 and other BLZ images
Labriformes	Labridae	*Xyrichtys*	*novacula*	Y	Fig. 26 and other BLZ images
Labriformes	Pomacentridae	*Abudefduf*	*saxatilis*	Y	Fig. 24
Labriformes	Pomacentridae	*Chromis*	*cyanea*	Y	Fig. 24
Labriformes	Pomacentridae	*Stegastes*	*planifrons*	Y	Fig. 24 and other BLZ images
Labriformes	Pomacentridae	*Stegastes*	*variabilis*	Y	Fig. 24 and other BLZ images
Labriformes	Pomacentridae	*Stegastes*	*diencaeus*	Y	BLZ 8452, 8404
Labriformes	Pomacentridae	*Stegastes*	*adustus*	Y	BLZ 7046
Labriformes	Pomacentridae	*Stegastes*	*leucostictus*	Y	BLZ 4585, 8472 and others
Labriformes	Pomacentridae	*Stegastes*	*partitus*	Y	Fig. 24 and other BLZ images
Labriformes	Pomacentridae	*Amblyglyphidodon*	*ternatensis*	Y	Fig. 24
Labriformes	Scaridae	*Cryptotomus*	*roseus*	Y	Fig. 23 and other BLZ images
Labriformes	Scaridae	*Scarus*	*iseri*	Y	Fig. 23 and other BLZ images
Labriformes	Scaridae	*Sparisoma*	*radians*	Y	Fig. 23 and other BLZ images
Labriformes	Scaridae	*Sparisoma*	*chrysopterum*	Y	Fig. 23 and other BLZ images
Labriformes	Scaridae	*Sparisoma*	*atomarium*	Y	Fig. 23 and other BLZ images
Lophiiformes	Antennariidae	*Antennarius*	*pauciradiatus*	Y	Fig. 27
Lophiiformes	Unknown	Unknown	Unknown	Y	http://www.fisheggsandlarvae.com/DIIIA4%20Lophiiformes.htm
Lophiiformes	Unknown	Unknown	Unknown	Y	Fig. 27
Lophiiformes	Unknown	Unknown	Unknown	Y	Fig. 27
Lophiiformes	Unknown	Unknown	Unknown	Y	Fig. 27
Mugiliformes	Mugilidae	*Mugil*	sp.	Y	BLZ 4506,
Mugiliformes	Mugilidae	*Mugil*	sp.	Y	Fig. 11 and other BLZ images
Mugiliformes	Mugilidae	*Mugil*	*cephalus*	Y	Fig. 11
Mugiliformes	Mugilidae	*Liza*	*tricuspidens*	Y	http://www.fisheggsandlarvae.com/LIIB9%20Mugilidae.htm
Ophidiiformes	Carapidae	*Carapus*	*bermudensis*	Y	Fig. 28 and other BLZ images
Ophidiiformes	Ophidiidae	*Parophidion*	*schmidti*	Y	Fig. 28 and other BLZ images
Ophidiiformes	Ophidiidae	*Brotulataenia*	sp.	Y	Fig. 29
Ophidiiformes	Ophidiidae	*Lampogrammus?*	sp.	Y	Fig. 29
Perciformes	Apogonidae	*Apogon*	*aurolineatus*	Y	Fig. 2 and other BLZ images
Perciformes	Apogonidae	*Apogon*	*binotatus*	y	BLZ 6331 and others (Baldwin *et al*., 2011)
Perciformes	Apogonidae	*Apogon*	sp. 1	Y	BLZ 5260 and others (Baldwin *et al*., 2011)
Perciformes	Apogonidae	*Apogon*	*phenax*	Y	BLZ 6361 and other (Baldwin *et al*., 2011)
Perciformes	Apogonidae	*Apogon*	*townsendi*	Y	BLZ 6329 and others (Baldwin *et al*., 2011)
Perciformes	Apogonidae	*Apogon*	*maculatus*	Y	BLZ 7717 and others (Baldwin *et al*., 2011)
Perciformes	Apogonidae	*Apogon*	*mosavi*	Y	BLZ 7713 and others (Baldwin *et al*., 2011)
Perciformes	Apogonidae	*Astrapogon*	*puncticulatus*	Y	Fig. 32 and others (Baldwin *et al*., 2009a)
Perciformes	Apogonidae	*Astrapogon*	*alutus*	Y	BLZ 6040 and others (Baldwin *et al*., 2009b)
Perciformes	Apogonidae	*Astrapogon*	*stellatus*	Y	BLZ 6038 and others (Baldwin *et al*., 2009b)
Perciformes	Apogonidae	*Phaeoptyx*	*pigmentaria*	Y	BLZ 7013 and others (Baldwin *et al*., 2009b)
Perciformes	Apogonidae	*Phaeoptyx*	*conklini*	Y	BLZ 5039 and others (Baldwin *et al*., 2009b)
Perciformes	Apogonidae	*Phaeoptyx*	*xenus*	Y	Fig. 32 and others (Baldwin *et al*., 2009a)
Perciformes	Chaetodontidae	*Chaetodon*	*capistratus*	Y	Fig. 30 and other BLZ images
Perciformes	Chaetodontidae	*Chaetodon*	*marleyi*	Y	Fig. 31
Perciformes	Gerreidae	*Eucinostomus*	*jonesi*	Y	Fig. 33 and other BLZ images
Perciformes	Gerreidae	*Eucinostomus*	*harengulus*	Y	Fig. 33 and other BLZ images
Perciformes	Gerreidae	*Eucinostomus*	*gula*	Y	Fig. 33 and other BLZ images
Perciformes	Gerreidae	*Eucinostomus*	*melanopterus*	Y	Fig. 33 and other BLZ images
Perciformes	Haemulidae	*Haemulon*	*aurolineatum*	Y	BLZ 10013
Perciformes	Haemulidae	*Anisotremus*	*virginicus*	Y	Fig. 35 and other BLZ images
Perciformes	Haemulidae	*Haemulon*	*plumieri*	Y	Fig. 35 and other BLZ images
Perciformes	Haemulidae	*Haemulon*	*sciurus*	Y	Fig. 35 and other BLZ images
Perciformes	Haemulidae	*Haemulon*	*flavolineatum*	Y	BLZ 10221 and other BLZ images
Perciformes	Haemulidae	*Pomadasys*	*commersonnii*	Y	http://www.fisheggsandlarvae.com/EIIIB3%20Haemulidae.htm
Perciformes	Scorpididae	*Neoscorpis*	*lithophilus*	Y	http://www.fisheggsandlarvae.com/FIIB1%20Neoscorpis.htm
Perciformes	Lutjanidae	*Lutjanus*	*mahogoni*	Y	Fig. 34
Perciformes	Lutjanidae	*Lutjanus*	*analis*	Y	Fig. 34 and other BLZ images
Perciformes	Lutjanidae	*Lutjanus*	*synagris*	Y	Fig. 34 and other BLZ images
Perciformes	Lutjanidae	*Lutjanus*	*vivanus*	Y	Fig. 34
Perciformes	Lutjanidae	*Lutjanus*	*apodus*	Y	BLZ 8429 and others
Perciformes	Lutjanidae	*Lutjanus*	*griseus*	Y	Fig. 34 and other BLZ images
Perciformes	Lutjanidae	*Ocyurus*	*chrysurus*	Y	Fig. 34 and other BLZ images
Perciformes	Opistognathidae?	Unknown	Unknown	Y	Fig. 36 and other BLZ images
Perciformes	Oplegnathidae	*Oplegnathus*	*robinsoni*	Y	http://www.fisheggsandlarvae.com/EIIIA2%20Oplegnathidae.htm
Perciformes	Oplegnathidae	*Oplegnathus*	*conwayi*	Y	http://www.fisheggsandlarvae.com/FIIA7%20Oplegnathidae.htm
Perciformes	Pempheridae	*Pempheris*	*schwenkii*	Y	http://www.fisheggsandlarvae.com/LIIA2%20Pempheridae.htm
Perciformes	Pomacanthidae	*Holacanthus*	*ciliarus*	Y	Fig. 30 and other BLZ images
Perciformes	Pomacanthidae	*Pomacanthus*	*arcuatus*	Y	Fig. 30 and other BLZ images
Perciformes	Pomacanthidae	*Pomacanthus*	*paru*	Y	BLZ 10213
Perciformes	Pomacanthidae	*Pomacanthus*	*rhomboides*	Y	Fig. 31
Perciformes	Sciaenidae	*Atractoscion*	*aequidens*	Y	http://www.fisheggsandlarvae.com/LIIA6%20Atractoscion.htm
Perciformes	Sciaenidae	*Equetus*	*punctatus*	Y	BLZ 10121
Perciformes	Sciaenidae	*Odontoscion*	*dentex*	Y	Fig. 36
Perciformes	Scorpididae	*Neoscorpis*	*lithophilus*	Y	http://www.fisheggsandlarvae.com/FIIB1%20Neoscorpis.htm
Perciformes	Sparidae	*Calamus?*	sp.	Y	Fig. 35
Perciformes	Sparidae	*Calamus?*	sp.	Y	Fig. 35 and other BLZ images
Pleuronectiformes	Achiridae	*Trinectes*	sp.	Y	Fig. 37 and other BLZ images
Pleuronectiformes	Bothidae	*Bothus*	*maculiferus*	Y	Fig. 37 and other BLZ images
Pleuronectiformes	Bothidae	*Bothus*	*ocellatus*	Y	Fig. 37 and other BLZ images
Pleuronectiformes	Bothidae	Unknown	Unknown	Y	Fig. 38
Pleuronectiformes	Paralichthyidae	*Citharichthys?*	sp.	Y	Fig. 37 and other BLZ images
Pleuronectiformes	Paralichthyidae	*Syacium*	sp.	Y	Fig. 37
Pleuronectiformes	Paralichthyidae	*Syacium*	sp.	Y	Fig. 37 and other BLZ images
Pleuronectiformes	Paralichthyidae	*Syacium*	sp.	Y	Fig. 37 and other BLZ images
Pleuronectiformes	Paralichthyidae	Unknown	Unknown	Y	BLZ 7086
Pleuronectiformes	Cynoglossidae	*Symphurus*	sp.	Y	Fig. 37 and other BLZ images
Pleuronectiformes	Soleidae	*Solea*	*turbynei*	Y	http://www.fisheggsandlarvae.com/MIIIA5%20Solea%20bleekeri.htm
Scombriformes	Scombridae	*Auxis*	*rochei*	Y	http://www.fisheggsandlarvae.com/LIIIA11%20Auxis.htm
Scombriformes	Sphyraenidae?	Unknown	Unknown	Y	http://www.fisheggsandlarvae.com/EIIIA2A%20Sphyraenidae.htm
Scombriformes	Sphyraenidae	*Sphyraena*	*barracuda*	Y	Fig. 39 and other BLZ images
Scombriformes	Gempylidae	Unknown	Unknown	Y	Fig. 39
Scombriformes	Istiophoridae	Unknown	Unknown	Y	Fig. 39
Scombriformes	Scombridae	Unknown	Unknown	Y	Fig. 39
Scorpaeniformes	Peristediidae	*Peristedion*	sp.	Y	Fig. 41
Scorpaeniformes	Scorpaenidae	*Scorpaena*	*inermis*	Y	Fig. 40 and other BLZ images
Scorpaeniformes	Scorpaenidae	*Scorpaena*	*bergii*	Y	Fig. 40 and other BLZ image
Scorpaeniformes	Scorpaenidae	*Scorpaena*	*grandicornis*	Y	Fig. 40 and other BLZ images
Scorpaeniformes	Scorpaenidae	*Scorpaenodes*	*caribbaeus*	Y	Fig. 40
Scorpaeniformes	Unknown	*Dendrochirus*	*brachypterus*	Y	Fig. 40
Scorpaeniformes	Serranidae	*Diplectrum*	*bivittatum*	Y	Fig. 42 and other BLZ images
Scorpaeniformes	Serranidae	*Pseudanthias*	*cooperi*	Y	Connell pers. comm.
Scorpaeniformes	Serranidae	*Gonioplectrus*	*hispanus*	Y	Fig. 45
Scorpaeniformes	Serranidae	*Hypoplectrus*	sp.	Y	Fig. 42 and other BLZ images
Scorpaeniformes	Serranidae	*Mycteroperca*	*bonaci*	Y	Fig. 45 and other BLZ images
Scorpaeniformes	Serranidae	*Pseudogramma*	*gregoryi*	Y	Fig. 43, 44, and other BLZ images
Scorpaeniformes	Serranidae	*Pseudogramma*	*polyacanthum*	Y	Fig. 44
Scorpaeniformes	Serranidae	*Rypticus*	sp.	Y	Fig. 43 and other BLZ images
Scorpaeniformes	Serranidae	*Rypticus*	sp.	Y	Fig. 43
Scorpaeniformes	Serranidae	*Rypticus*	sp.	Y	Fig. 43
Scorpaeniformes	Serranidae	*Bathyanthias*	sp.	Y	Fig. 46
Scorpaeniformes	Serranidae	*Liopropoma*	*rubre*	Y	Fig. 46
Scorpaeniformes	Serranidae	*Serranus*	*tigrinus*	Y	BLZ 7730, 5352
Scorpaeniformes	Serranidae	*Serranus*	*baldwini*	Y	Fig. 42 and other BLZ images
Stromateiformes	Nomeidae	*Cubiceps*?	Unknown	Y	Fig. 48
Stromateiformes	Stromateidae	*Centrolophus*	*niger*	Y	http://www.fisheggsandlarvae.com/FIIA5%20Centrolophus%20niger.htm
Tetraodontiformes	Monacanthidae	*Monacanthus*	*ciliatus*	Y	Fig. 50 and other BLZ images
Tetraodontiformes	Monacanthidae	*Aluterus*	*schoepfi*	Y	Fig. 50
Tetraodontiformes	Ostraciidae	Unknown	Unknown	Y	Fig. 50 and other BLZ images
Tetraodontiformes	Ostraciidae	*Lactophrys*	*trigonus*	Y	Fig. 50 and other BLZ images
Tetraodontiformes	Ostraciidae	Unknown	Unknown	Y	Fig. 51
Tetraodontiformes	Tetraodontidae	*Canthigaster*	*rostrata*	Y	Fig. 49 and other BLZ images
Tetraodontiformes	Tetraodontidae	*Sphoeroides*	*testudineus*	Y	Fig. 49 and other BLZ images
Tetraodontiformes	Tetraodontidae	*Sphoeroides*	*spengleri*	Y	Fig. 49 and other BLZ images
Tetraodontiformes	Tetraodontidae	*Ranzania*	*laevis*	Y	Fig. 49
Trachiniformes	Champsodontidae	*Champsodon*	*capensis*	N	http://www.fisheggsandlarvae.com/LIIB1%20Champsodontidae.htm
Trachiniformes	Chiasmodontidae	*Chiasmodon*	*niger*	N	http://www.fisheggsandlarvae.com/LIIA8%20Chiasmodontidae.htm
Trachiniformes	Uranoscopidae	Unknown	Unknown	Y	http://www.fisheggsandlarvae.com/CMIA3%20Uranoscopidae.htm

BLZ, Belize. In cases in which it is followed by a four- or five-digit number, this represents the Smithsonian DNA number.
